# Construction of yeast with extremely high 2,3-butanediol tolerance by introducing point and structural mutations and partial elucidation of the mechanism of 2,3-butanediol tolerance

**DOI:** 10.1007/s00253-025-13626-8

**Published:** 2025-10-16

**Authors:** Kaito Nakamura, Ryosuke Yamada, Rumi Sakaguchi, Takuya Matsumoto, Hiroyasu Ogino

**Affiliations:** Department of Chemical Engineering, Osaka Metropolitan University, 1-1 Gakuen-Cho, Naka-Ku, Sakai, Osaka 599-8531 Japan

**Keywords:** 2,3-Butanediol, Mutagenesis, *Saccharomyces cerevisia*e, Stress tolerance, Transcriptome analysis, Yeast

## Abstract

**Abstract:**

Microbial production of valuable chemicals is a promising and sustainable approach, offering high energy efficiency and minimal waste generation. Production of 2,3-butanediol (2,3-BDO) by the safe industrial yeast *Saccharomyces cerevisiae* holds potential as a sustainable bioprocess. However, the low tolerance of 2,3-BDO in yeast remains a major challenge. In this study, we aimed to improve 2,3-BDO tolerance in *S. cerevisiae* by introducing DNA point and structural mutations using techniques developed in previous studies, thereby advancing the sustainable industrial production of 2,3-BDO. Through point and structural mutagenesis, we successfully obtained the mutant strain YPH499/Co58, which exhibited a 122-fold higher OD_600_ value than the parent strain after 96 h of cultivation in a medium containing 175 g/L 2,3-BDO. Transcriptome analysis of four mutants with particularly high 2,3-BDO tolerance suggested that the upregulation of genes related to the proteasome, peroxisome, TCA cycle, mitochondria, and transcriptional regulation was closely related to 2,3-BDO tolerance. The use of these mutant strains represents a major step toward realizing the sustainable industrial production of 2,3-BDO. Additionally, the insights gained in this study regarding 2,3-BDO tolerance may contribute to improving yeast tolerance to various stresses, including ethanol, heat, and low pH. The mutagenesis technique developed in this study holds promise for the construction of yeast strains with enhanced robustness for various applications.

**Key points:**

- *DNA point and structural mutations enhanced 2,3-BDO tolerance in yeast.*
- *Engineered yeast mutant showed 122-fold higher growth in 175 g/L 2,3-BDO.*
- *Transcriptome analysis revealed key factors for 2,3-BDO tolerance.*

**Supplementary Information:**

The online version contains supplementary material available at 10.1007/s00253-025-13626-8.

## Introduction

In the context of global warming and depletion of fossil resources, the production of chemicals from renewable plant biomass using microorganisms has garnered increasing attention (Tyo et al. 2007). Among these microorganisms, yeast is safe to use and can be easily cultivated on an industrial scale and genetically manipulated; therefore, it is expected to serve as a platform for the production of various chemicals from biomass resources (Ostergaard et al. 2000).

2,3-Butanediol (2,3-BDO), a chemical typically produced from petroleum, is a promising bulk chemical with a wide range of applications (Huo et al. 2022; Ji et al. 2011). However, 2,3-BDO has become expensive due to high petroleum prices and expensive chemical catalysts that facilitate the synthesis of the unique diol structure (Białkowska 2016). Therefore, there is an urgent requirement for the establishment of sustainable production technology that uses microorganisms to produce 2,3-BDO from biomass (Ji et al. 2011; Yang and Zhang 2019).

Recently, *Saccharomyces cerevisiae* has been studied as a promising microorganism for 2,3-BDO production (Mitsui et al. 2022). In previous studies, metabolic engineering of *S. cerevisiae* achieved the production of 2,3-BDO at a rate surpassing that of 2,3-BDO-producing pathogenic bacteria using batch culture (Ma et al. 2009; Yamada et al. 2017). Furthermore, very high 2,3-BDO production rates were achieved using continuous culture, which is important for industrial production (Yamada et al. 2018). However, due to the low tolerance of yeast to 2,3-BDO, the production of high concentrations of 2,3-BDO remains a challenge (Mitsui et al. 2022; Mizobata et al. 2021; Yamada et al. 2017). Consequently, it is important to improve the 2,3-BDO tolerance of yeast to achieve a more sustainable industrial production of 2,3-BDO.

In general, because a large number of non-specific genes are involved in the stress tolerance of yeast, it is difficult to improve specific stress tolerance by modifying a specific gene (Cakar et al. 2005; Shiwa et al. 2012). Therefore, it is necessary to comprehensively modify the expression of genes involved in the stress response to obtain stress-tolerant strains. Attempts have been made to introduce random mutations into yeast to improve its tolerance to various types of stress. Mutations introduced into microorganisms can be broadly classified as DNA point mutations and structural mutations. It has been shown that overexpression of mutant DNA polymerase δ, which lacks proofreading function, into yeast leads to point mutations due to errors in DNA replication (Abe et al. 2009; Shiwa et al. 2012). Furthermore, a technique has been developed to introduce DNA structural mutations by inducing double-strand breaks in repeat sequences, known as δ sequences, using clustered regularly interspaced short palindromic repeats (CRISPR) and CRISPR-associated (Cas) systems (Mitsui et al. 2019; Pâques and Haber 1999). Hundreds of δ sequences are distributed throughout the yeast genome (Dujon 1996). The CRISPR system can be utilized to create double-stranded breaks in these δ sequences, enabling the introduction of structural mutations during genome reconstruction. By combining these techniques to introduce point and structural mutations simultaneously, we have succeeded in developing mutant yeast strains that exhibit tolerance to organic acids (Mitsui et al. 2020) and organic solvents (Mizobata et al. 2021). In previous studies, the Cas9 protein of the CRISPR system was expressed using a galactose-inducible promoter to induce structural mutations (Mitsui et al. 2020; Mizobata et al. 2021). The use of this inducible system was one factor that reduced the efficiency of mutagenesis. Additionally, the use of a galactose-inducible promoter also limited the carbon source during mutation introduction.

In this study, we aimed to improve yeast tolerance to 2,3-BDO by introducing DNA point and structural mutations, thereby contributing to the sustainable industrial production of 2,3-BDO. First, to facilitate efficient mutagenesis, a new mutagenesis plasmid was constructed by replacing the galactose-inducible promoter of the plasmid developed in the previous study (Yamada et al. 2024a, b) with a constitutive expression promoter. Next, 2,3-BDO-tolerant mutant yeast strains were constructed using the engineered mutagenesis plasmid. Finally, the mechanisms underlying 2,3-BDO tolerance in the mutant yeast strains were investigated using transcriptome analysis.

## Material and methods

### Microorganisms and culture media

The strains and plasmids used in this study are listed in Table 1. *Escherichia coli* NEB5α (New England Biolabs Japan, Tokyo, Japan) was used as the host for recombinant DNA manipulation. The cells were cultured in Luria–Bertani broth (20 g/L LB broth powder [Nacalai Tesque, Kyoto, Japan]) supplemented with 100 µg/mL ampicillin sodium salt. *S. cerevisiae* YPH499 (NBRC 10505) was used as the host. Yeast was cultured in yeast/peptone/glucose (YPD) medium (10 g/L yeast extract [Formedium, Norfolk, UK], 20 g/L peptone [Formedium], 20 g/L glucose) or synthetic glucose (SD) medium (6.7 g/L yeast nitrogen base without amino acids [Formedium], 20 g/L glucose, appropriate amino acids, and nucleic acids). Predetermined amounts of agar, 2,3-BDO, or ethanol were added to the medium as needed. If necessary, the medium’s pH was adjusted to 2.5 with 50 mM phosphate before use. SD medium containing 0.05% 5-fluoroanthranilic acid was used to remove plasmids containing the *TRP1* selection marker.

**Table 1:** Strains and plasmids

Strains and plasmids	Relevant features	Reference
*Escherichia coli* strain		
*NEB5α*	*fhuA2Δ(argF-lacZ)U169 phoA glnV44 Φ80Δ(lacZ)M15 gyrA96 recA1 relA1 endA1 thi-1 hsdR17*	New England Biolabs Japan
*Saccharomyces cerevisiae* strain		
YPH499	*MAT*a *ura3-52 lys2-801_amber ade2-101_ochre trp1-*Δ*63 his3-*Δ*200 leu2-*Δ*1*	NBRC10505; NITE Biological Resource Center
YPH499/W	YPH499 transformed with pRS404	This study
YPH499/pMSM_Co	YPH499 transformed with pEWPMSM_Co	This study
YPH499/PMSM_CoX (X; 1–94)	Mutation-introduced YPH499/pMSM_Co	This study
YPH499/CoX (X: 36, 40, 53, 58)	Removal of plasmid pEWPMSM_Co from YPH499/PMSM_CoX (X: 36, 40, 53, 58)	This study
Plasmid		
pRS404	Empty plasmid containing the *TRP1* selection marker	Stratagene, CA, USA
pEWPMSM	Plasmid for expressing proofreading-deficient DNA polymerase δ by *POL3* promoter and StCas9 and δ-sequence-targeting gRNA by *GAL1* promoter with *TRP1* as selection marker	Yamada et al. 2024a
pEWPMSM_Co	Plasmid for expressing proofreading-deficient DNA polymerase δ by *POL3* promoter and StCas9 and δ-sequence-targeting gRNA by TEF*1* promoter with *TRP1* as selection marker	This study

### Culture method

Cell culture was carried out in 1 mL of YPD medium containing 150 g/L 2,3-BDO for 48 h in a 2-mL 96 deep-well plate equipped with a gas permeable seal and incubated on a rotary plate shaker set at 30 °C and 1500 rpm. The cultivation was initiated by inoculation (5% vol/vol) of a preculture grown in a microplate containing SD medium for 48 h at 30 °C and 1500 rpm.

Flask cultures were performed in baffled 500-mL flasks containing 100 mL of YPD medium in a rotary shaker set at 30 °C and 150 rpm. Cultivation was initiated by inoculation (initial OD_600_ = 0.05) of a preculture grown for 96 h at 30 °C in a test tube containing SD medium at 150 rpm.

Test tube cultures were performed in 5 mL of SD medium containing a predetermined amount of 2,3-BDO and incubated on a rotary shaker at 30 °C and 150 rpm. Cultivation was initiated by inoculation (initial OD_600_ = 0.3).

### Plasmid construction and transformation

The primers used in this study are listed in Table 2. The pTEF-pRPR(F)_Ass and pTEF-Cas(R)_Ass primers were used to amplify the *TEF1* promoter by PCR using genomic DNA of *S. cerevisiae* YPH499 strain as a template. Additionally, Cas-pTEF(F)_Ass and pRPR-pTEF(R)_Ass were used to amplify the *POL3-D321A/E323A* genes (encoding proofreading function-deficient DNA polymerase δ), StCas9 (encoding Cas9 protein from *Streptococcus thermophilus*), and a guide RNA for the *S. cerevisiae* δ sequence by PCR using plasmid pEWPMSM (Yamada et al. 2024a, b) as a template. The two DNA fragments were ligated using the NEBuilder HiFi DNA Assembly Master Mix (New England Biolabs). The constructed plasmid was named pEWPMSM_Co (Supplementary Fig. S1). The design and efficacy of gRNAs were verified in a previous publication (Mitsui et al. 2019).

**Table 2:** PCR primers

Primers	Sequence (5′−3′)
Cas-pTEF(F)_Ass	GCATTCTAATCTAAGTTTTAATTACAAAAAAAAACCTCTAGATGGCTGATAAGCCAGGTC
pRPR-pTEF(R)_Ass	AGCTATGGTGTGTGGTTVTAGACTCGAGGATCTGCCAATTG
pTEF-pRPR(F)_Ass	CAGATCCTCGAGTCTAGAACCACACACCATAGCTTCAAAATG
pTEF-Cas(R)_Ass	GGACCTGGCTTATCAGCCATCTAGAGGTTTTTTTTTGTAATTAAAACTTAGATTAG

### Construction of yeast strain

The constructed mutagenesis plasmid pEWPMSM_Co was introduced into the yeast using the lithium acetate method (Chen et al. 1992). Yeast transformants were cultivated in SD medium containing a predetermined amount of 2,3-BDO within test tubes to enrich for 2,3-BDO-tolerant mutant strains. After the enrichment cultures, 2,3-BDO-tolerant mutant strains were separated on SD agar medium.

### Analysis of proliferation

Optical density at 600 nm was measured using a UVmini-1240 spectrophotometer (Shimadzu, Kyoto, Japan) or a Multiskan GO microplate reader (Thermo Scientific, Rochester, NY, USA).

### Transcriptome analysis

Total RNA extraction and complementary DNA library construction for RNA sequencing were conducted according to a previously described method (Karitani et al. 2024). RNA sequencing was performed using DNBSEQ-G400 (MGI Tech, Shenzhen, China). The genome sequence of *S. cerevisiae* strain S288c was used as a reference for read mapping using Geneious (Tomy Digital Biology, Tokyo, Japan). Differential expression log2 ratios and *p*-values were calculated, and differentially expressed genes (DEGs) were identified using Geneious software. RNA sequencing data were deposited in the DDBJ nucleotide sequence database under the accession number PRJDB19977.

### Gene ontology enrichment analysis

DEGs were used for GO enrichment analysis using Metascape (Zhou et al. 2019). For GO enrichment analysis, molecular function, biological processes, and cellular component GO terms were selected.

## Results

### Construction and screening of 2,3-BDO-tolerant mutants

YPH499/PMSM_Co was constructed by introducing the mutagenesis plasmid pEWPMSM_Co into the parent strain YPH499. YPH499/PMSM_Co was cultured in SD medium containing 50 g/L 2,3-BDO, and OD_600_ was measured every 24 h. When the OD_600_ exceeded 1.0, the cells were inoculated into fresh SD medium containing an additional 10 g/L of 2,3-BDO (initial OD_600_ = 0.3). The same procedure was repeated with the 2,3-BDO concentration gradually increased from 50 to 210 g/L. Next, 94 mutants grown in SD medium containing 210 g/L 2,3-BDO were separated and labelled as YPH499/PMSM_CoX (X; 1–94). For comparison, the empty plasmid pRS404 containing *TRP1* was introduced into YPH499 to construct YPH499/W with complementary tryptophan auxotrophy.

**Fig. 1:**
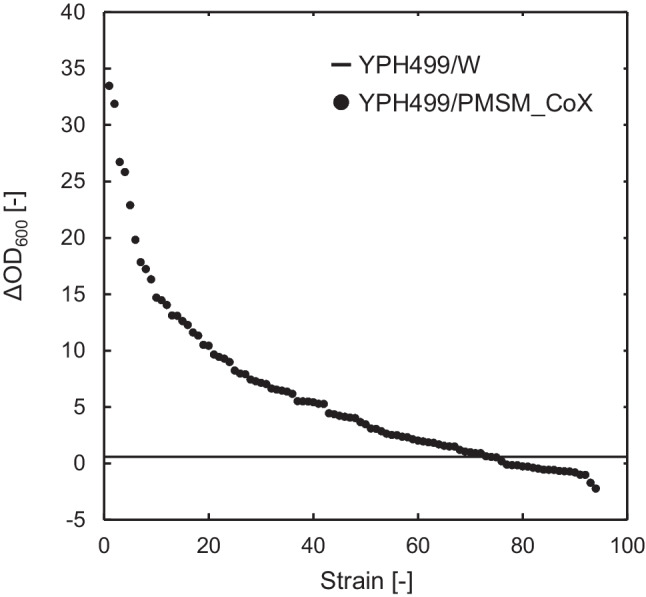
ΔOD_600_ of mutant strains YPH499/PMSM_CoX (X: 1–94) after 48 h of cultivation in YPD medium containing 150 g/L 2,3-BDO. Mutant strains are presented in descending order from left to right. The ΔOD_600_ value of the parental strain is represented by a horizontal line. Data is presented from a single experiment

YPH499/PMSM_CoX was cultured in microplates for 48 h. Subsequently, its ΔOD_600_ was measured after 48 h of cultivation in YPD medium containing 150 g/L 2,3-BDO (Fig. 1). A single high-throughput experiment was conducted to select candidate tolerant strains from numerous mutants. Of the 94 mutants, 75 showed higher ΔOD_600_ values than the parental strain YPH499/W. Next, to exclude the effect of improved growth in the absence of 2,3-BDO and to ensure the reproducibility of 2,3-BDO tolerance, the relative OD_600_ in the presence of 2,3-BDO compared to its absence was evaluated (Supplementary Fig. S2). Consequently, four mutants, YPH499/PMSM_CoX (X: 36, 40, 53, 58), which showed particularly high 2,3-BDO tolerance, were selected.

### Evaluation of 2,3-BDO stress tolerance in the selected mutants

The mutagenesis plasmid pEWPMSM_Co was removed from the selected mutant strain YPH499/PMSM_CoX (X: 36, 40, 53, 58) to obtain YPH499/CoX (X: 36, 40, 53, 58).

**Fig. 2:**
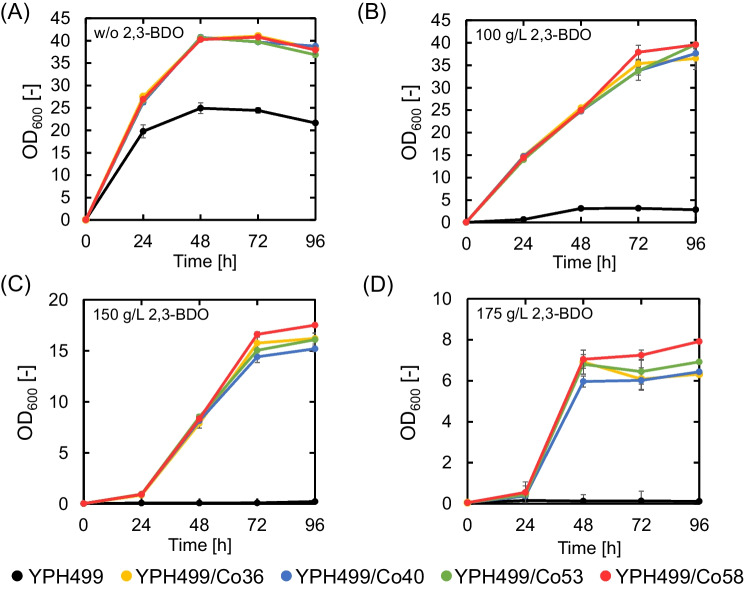
Growth curves of mutant strains YPH499/CoX (X: 36, 40, 53, 58) and its parent strain YPH499 in YPD medium containing **A** 0 g/L, **B** 100 g/L, **C** 150 g/L, and **D** 175 g/L of 2,3-BDO. Data are presented as the average of three independent experiments. Error bars represent standard deviation

YPH499 and YPH499/CoX (X: 36, 40, 53, 58) cells were cultured in flasks containing YPD liquid medium or YPD liquid medium containing 100, 150, or 175 g/L 2,3-BDO (Fig. 2). As shown in Fig. 2A, in the absence of 2,3-BDO, the OD_600_ values of the mutant YPH499/CoX (X: 36, 40, 53, 58) after 96 h of incubation were 38.2, 38.8, 36.8, and 37.9, respectively, which were approximately 1.8 times higher than the OD_600_ value of YPH499. As shown in Fig. 2B, C, and D, YPH499/CoX (X: 36, 40, 53, 58) grew significantly faster than YPH499 in the presence of 100, 150, and 175 g/L 2,3-BDO. Particularly, YPH499/Co58 showed 14-, 89-, and 122-fold higher OD_600_ values than YPH499 after 96 h of cultivation in a medium containing 100, 150, and 175 g/L 2,3-BDO, respectively.

### Evaluation of various stress tolerance of selected yeasts

To evaluate the tolerance of the parent YPH499 and mutant YPH499/CoX (X: 36, 40, 53, 58) strains to various stresses other than 2,3-BDO, these strains were cultured under ethanol, heat, and low pH stress conditions.

**Fig. 3:**
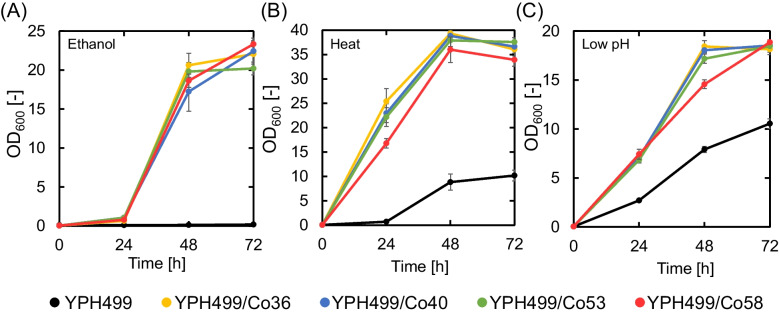
Growth curve of mutant strains YPH499/CoX (X: 36, 40, 53, 58) and the parent strain YPH499 under three conditions: ethanol stress (containing 70 g/L ethanol), heat stress (cultured at 37 °C), and low pH stress (buffered at pH 2.5). Data are presented as the average of three independent experiments. Error bars represent standard deviation

Flask cultures were subjected to three conditions: ethanol stress (70 g/L ethanol), heat stress (37 °C), and low pH stress (pH 2.5) (Fig. 3). The 2,3-BDO-tolerant mutant strain YPH499/CoX (X: 36, 40, 53, 58) showed higher growth capability than that of the parent strain YPH499 under ethanol, heat, and low pH stress conditions. After 72 h of cultivation of the mutant YPH499/Co58 under ethanol, heat, and low pH stress conditions, the OD_600_ values were 63, 1.8, and 3.3 times higher than those of YPH499, respectively. These results indicated that the selected mutants YPH499/CoX (X: 36, 40, 53, 58) acquired tolerance to 2,3-BDO, ethanol, heat, and low pH.

### Transcriptome analysis

As shown in Fig. 2, YPH499/CoX (X: 36, 40, 53, 58) showed higher growth capability than YPH499 in the presence of 2,3-BDO. Therefore, transcriptome analysis was performed to determine the reason for the high tolerance of the acquired mutant strains to 2,3-BDO.

**Fig. 4:**
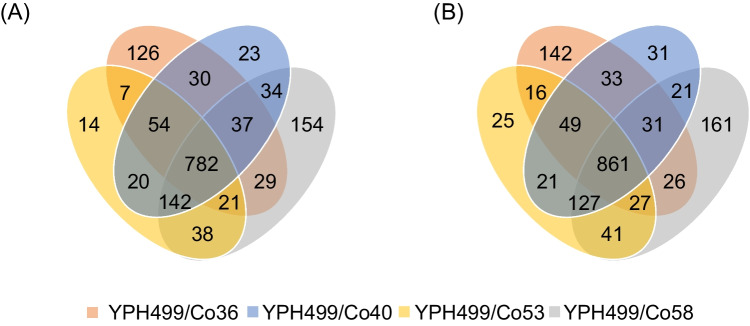
Venn diagram showing the number of genes with **A** increased expression and **B** decreased expression in selected mutant strains YPH499/CoX (X: 36, 40, 53, 58)

The parent strain YPH499 and mutant strain YPH499/CoX (X: 36, 40, 53, 58) were cultured in YPD medium containing 100 g/L 2,3-BDO for 48 h, and total RNA was extracted from the cells for sequencing. The log2 ratios (log2 fold changes) for each gene were calculated and plotted as volcano plots (Supplementary Fig. S3). Transcriptome analysis revealed increased expression of 1086, 1123, 1078, and 1237 genes and decreased expression of 1195, 1179, 1165, and 1283 genes in YPH499/CoX (X: 36, 40, 53, 58), respectively (Supplementary Tables S1–S8). Furthermore, 782 genes were identified as commonly upregulated and 861 genes were identified as commonly downregulated in all YPH499/CoX (X: 36, 40, 53, 58) mutants (Fig. 4 and Supplementary Table S9).

**Fig. 5:**
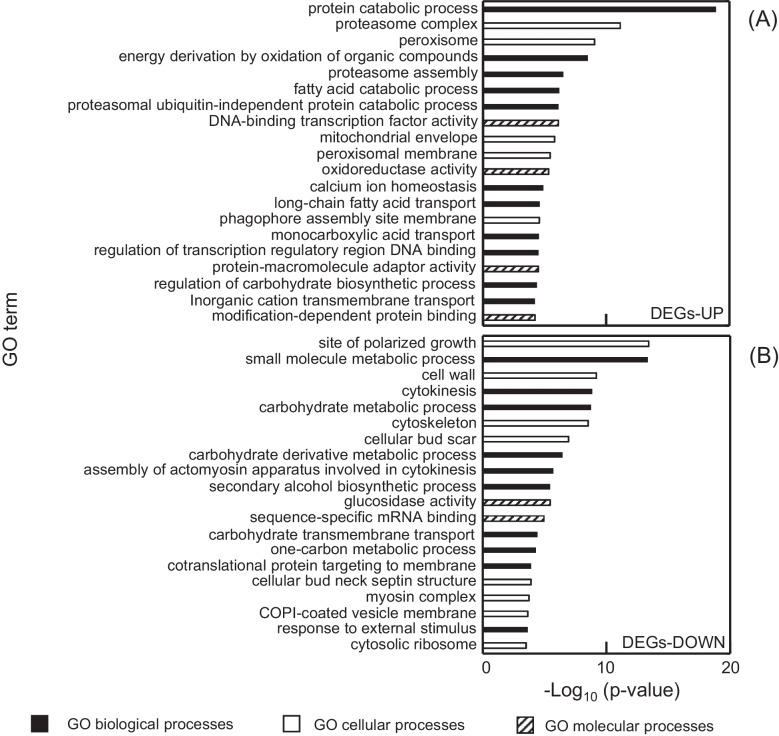
Gene ontology enrichment analysis of genes with common expression changes in selected mutants YPH499/CoX (X: 36, 40, 53, 58). **A** Upregulated genes, **B** downregulated genes. *P*-values were adjusted using the Benjamini–Hochberg procedure; the smallest -log10 *P*-value was 3.5

To understand the functional categories of the genes that were commonly altered among the four mutants, gene ontology (GO) enrichment analysis of the 782 commonly upregulated and 861 commonly downregulated genes was performed (Fig. 5). Figure 5A shows that the 2,3-BDO-tolerant mutant strains exhibited elevated expression levels of genes involved in protein catabolic processes, peroxisome complexes, and energy derivation by oxidation of organic compounds than the parent strain YPH499. In contrast, Fig. 5B shows that the 2,3-BDO-tolerant mutant strains showed decreased expression levels of genes involved in terms such as the site of polarized growth, small molecule metabolic process, and cell wall than the parent strain YPH499.

## Discussion

The mutant strain YPH499/PMSM_Co58 reached an OD_600_ value 122-fold higher than that of the parent strain when cultured for 96 h in a medium containing 175 g/L 2,3-BDO, demonstrating a considerably high 2,3-BDO tolerance (Fig. 2). Although there are limited studies on enhancing tolerance to 2,3-BDO in the yeast *S. cerevisiae*, a previous study reported the construction of a yeast mutant that grew to an OD_600_ value 4.9 times higher than that of the parent strain in a medium containing 175 g/L 2,3-BDO by simultaneously introducing DNA point and structural mutations that function under a galactose-inducible promoter (Mizobata et al. 2021). In contrast, in the present study, we constructed a mutant yeast by simultaneously introducing DNA point and structural mutations that function under a constitutive expression promoter. To the best of our knowledge, we succeeded in constructing a yeast mutant with a higher 2,3-BDO tolerance compared to that reported in previous studies. The 2,3-BDO concentration of 175 g/L was comparable to the highest reported levels of 2,3-BDO production by microorganisms, including pathogenic bacteria and yeasts (Mitsui et al. 2022). Unlike previous studies in which growth was strongly inhibited in the presence of high concentrations of 2,3-BDO (Mizobata et al. 2021), the 2,3-BDO-tolerant mutant yeast developed in our study grew vigorously even in the presence of 175 g/L 2,3-BDO, which is considered an achievement toward sustainable microbial industrial production of 2,3-BDO. In general, *S. cerevisiae* prefers glucose over galactose as a carbon source and grows faster in the presence of glucose (Lee et al. 2011). Therefore, by using glucose as a carbon source when constructing a mutant strain, it is possible to efficiently increase the number of generations and achieve a high mutagenesis efficiency. This may have contributed to the construction of a mutant strain with high 2,3-BDO tolerance in our study. The mutagenesis technique established in the present study allows for the introduction of point and structural mutations into yeast by simply introducing a plasmid into yeast and culturing it, thereby making it possible to easily and widely modify the yeast genome (Mitsui et al. 2019, 2020; Mizobata et al. 2021; Yamada et al. 2024a, 2024b); additionally, there are no restrictions on the carbon source used when introducing the mutations. Therefore, this mutagenesis technique can be used to construct various useful mutant yeasts in the future.

Genes commonly upregulated in the 2,3-BDO highly tolerant strain YPH499/CoX (X: 36, 40, 53, 58) included proteasome-related terms, such as protein catabolic process, proteasome complex, proteasome assembly, and proteasomal ubiquitin-independent protein catabolic process (Fig. 5A). Proteasomes degrade and remove unnecessary proteins, such as denatured proteins. Denatured proteins accumulate in cells under reactive oxygen species (ROS) stress, resulting in a significant decrease in growth, viability, and fermentation ability of yeasts (Tanahashi et al. 2024). Therefore, increased expression of genes controlling the proteasome may help alleviate inhibition of growth caused by denatured proteins. Furthermore, proteolysis by ubiquitin and proteasomes is thought to assist the DNA repair process by regulating the expression levels of proteins and enzymes involved in DNA repair and eliminating components that inhibit DNA repair (Spasskaya et al. 2020). In fact, genes whose expression was consistently elevated in all four mutants included *PRE1*, *RAD23*, and *RAD51*. The *PRE1* gene encodes a proteasome subunit, directly involved in protein degradation via the ubiquitin–proteasome system, and has been reported to contribute to various stress tolerances (Annan Robert et al. 2008; Heinemeyer et al. 1991). The *RAD23* gene, which encodes an ubiquitin protein, has been shown to be involved in DNA repair and may contribute to stress tolerance (Elder et al. 2002). Furthermore, the *RAD51* gene encodes a central protein in the homologous recombination repair pathway, playing a crucial role in DNA repair, and *RAD51* has also been reported to be involved in stress tolerance (Choi et al. 2018). These reports suggest that increased expression of genes involved in the proteasome may repair protein organization and DNA damage caused by 2,3-BDO stress and have a positive effect on the growth of yeast under 2,3-BDO stress.

Genes commonly upregulated in the 2,3-BDO highly tolerant strain YPH499/CoX (X: 36, 40, 53, 58) also included peroxisome-related terms such as peroxisome, fatty acid catabolic process, and peroxisomal membrane (Fig. 5A). Peroxisomes are cellular organelles that change in number in response to the environment and are involved in catabolic pathways such as fatty acid β-oxidation and the glyoxylate cycle (Mattiazzi et al. 2015) . Among these, fatty acid β-oxidation is one of the most important metabolic pathways that provide acetyl CoA for mitochondria during stress. Furthermore, an increase in the number of peroxisomes due to the activation of peroxisome biosynthesis is thought to activate mitochondrial respiration (Manzanares-Estreder et al. 2017). Thus, the activation of mitochondrial respiration by the activation of fatty acid β-oxidation occurring within peroxisomes and the increased peroxisome number likely contribute to 2,3-BDO tolerance.

In the highly 2,3-BDO-tolerant strain YPH499/CoX (X: 36, 40, 53, 58), upregulated genes were enriched in energy metabolism pathways, including the TCA cycle and mitochondria-related functions such as the mitochondrial envelope (Fig. 5A; Supplementary Table S9). Several TCA cycle genes (*CIT2*, *CIT3*, *MDH1*–*3*, *SDH2*, *SHH4*) showed increased expression, consistent with the importance of this pathway in ATP production (Flores et al. 2000). Mitochondria are intracellular organelles that play important roles in ATP production and cell death. Mitochondria produce ATP through redox reactions in the respiratory chain complex present on their inner membranes. Previous studies have shown that higher ATP levels promote protein aggregate dissociation and refolding, processes that enhance tolerance to various stresses (Święciło 2016). Notably, increased expression of *COX* genes involved in the mitochondrial respiratory chain and *AAC1* that transports ATP has been associated with improved acetic acid tolerance (Greetham et al. 2014). In addition, changes in the expression of the *ATP6* gene involved in ATP synthesis have been found to be related to ethanol stress (Phong et al. 2022). Therefore, the activation of the TCA cycle, *COX* genes, and ATP synthesis and transport likely contributes to sufficient ATP production, which in turn helps respond to the energy-intensive stress caused by 2,3-BDO.

Genes commonly upregulated in the 2,3-BDO highly tolerant strain YPH499/CoX (X: 36, 40, 53, 58) also included terms related to redox reactions, such as oxidoreductase activity (Fig. 5A). Previous studies have shown that when yeast is grown in media containing organic solvents, mitochondria are damaged by the organic solvents, causing mitochondrial fragmentation and the formation of ROS (Nishida-Aoki et al. 2015), which is known to cause programmed cell death (Jacobson 1996). Removal of the generated ROS requires antioxidant proteins such as catalase, peroxiredoxin, glutathione, and glutaredoxin (Lin et al. 2021), whose expression was elevated in this study (Supplementary Table S9). Deletion of the oxidative stress tolerance genes *ZTA1* and *UGA2* (Cao et al. 2013; Chen et al. 2019) has also been found to increase susceptibility to oxidative stress. Elimination of ROS by antioxidant proteins and increased tolerance to oxidative stress caused by ROS through increased expression of *ZTA1* and *UGA2* likely prevented programmed cell death and increased 2,3-BDO tolerance.

The four 2,3-BDO-tolerant mutant strains obtained in our study also showed high tolerance to ethanol, heat, and low pH (Fig. 3). Yeast acquires tolerance to various stresses by activating various transcription factors (Mira et al. 2010; Yamada et al. 2021). Therefore, we determined the transcription levels of 19 representative stress-related transcription factors (Xu et al. 2020) in the four 2,3-BDO-tolerant mutants obtained (Table 3). Of the 19 transcription factors, the expression levels of 17 transcription factors, excluding *HAP4* and *HAP5*, were increased in at least one mutant strain. The expression levels of eight transcription factors (*CUP2*,* GIS1*,* HAA1*,* HAP2*,* HOT1*,* HSF1*,* MSN2*, and* YAP1*) were significantly increased in all four mutants (Table 3). These transcription factors include those that respond to various organic solvents (*MSN2* and *YAP1*), heat stress (*HSF1*, *MSN2*, and *YAP1*), and oxidative stress (*HAA1*, *HSF1*, *MSN2*, and *YAP1*). Therefore, the increased expression of these transcription factors is one of the reasons why these 2,3-BDO-tolerant strains acquired tolerance to ethanol, heat, and low pH. In addition to the 19 representative transcription factors, at least 35 were found to be upregulated and 19 downregulated in all four mutants, respectively (Supplementary Table S9). Future studies focusing on these transcription factors may shed light on the tolerance mechanism specific to 2,3-BDO.

**Table 3:** Expression level of stress-related transcription factors

Gene	Log2 fold change relative to YPH499			
	**YPH499/Co36**	**YPH499/Co40**	**YPH499/Co53**	**YPH499/Co58**
*CUP2*	2.96	2.94	2.88	2.86
*GIS1*	2.72	2.59	2.55	2.75
*HAA1*	2.24	2.28	2.13	1.96
*HAP1*	0.72	0.42	0.28	0.38
*HAP2*	1.44	1.31	1.16	1.18
*HAP3*	0.58	0.39	0.15	0.40
*HAP4*	−0.19	−0.38	−0.63	−1.05
*HAP5*	−0.50	−0.99	−1.19	−1.52
*HOT1*	1.79	1.67	1.82	1.63
*HSF1*	1.94	1.80	1.63	1.54
*MSN2*	2.63	2.68	2.86	3.32
*MSN4*	−0.42	0.62	0.16	0.29
*NRG1*	0.72	0.29	0.28	0.42
*RPH1*	1.18	1.56	1.11	0.27
*RPN4*	0.62	0.66	0.63	0.62
*SFP1*	0.38	0.23	−0.05	−0.61
*SPT15*	1.63	1.52	1.48	0.66
*YAP1*	2.37	2.80	2.65	2.32
*YRR1*	−2.22	1.54	1.62	1.62

Expression levels were evaluated after 48 h of cultivation in YPD medium containing 100 g/L 2,3-BDO

The expression levels of heat shock protein (*HSP*) genes (*HSP10*, *HSP30*, *HSP60*, *HSP78*, *HSP150*) and protein folding-related genes (*SSA1*, *SSA2*, *PDI1*, *CPR6*, *SSE1*, *KAR2*) were reduced (Fig. 5B; Supplementary Table S9). These genes encode proteins that refold misfolded proteins into their native structures (Hasin et al. 2014). Increased expression of these genes has been found to improve tolerance to various stresses, such as heat stress and ethanol stress (Doğan et al. 2014; Hasin et al. 2014). However, in a previous study, ethanol-tolerant strains with decreased expression of *HSP* and *SSA1* genes were acquired along with increased expression of genes related to ubiquitin proteasomes (Xu et al. 2020). In this study, we also observed downregulated *HSP* and protein folding-related genes, along with upregulated ubiquitin–proteasome-related genes, in the 2,3-BDO-tolerant strain. This result supports the contribution of the proteasome to 2,3-BDO tolerance in *S. cerevisiae*.

In this study, we successfully constructed mutant yeast strains with extremely high 2,3-BDO tolerance by introducing DNA point and structural mutations. Furthermore, transcriptome analysis of the mutants revealed various factors associated with 2,3-BDO tolerance in yeast, such as proteasomes, peroxisomes, the TCA cycle, mitochondria, and transcription factors. While research on 2,3-BDO tolerance in yeast is limited, previous studies have reported that increased expression of numerous genes contributes to tolerance to ethanol and heat stress. These genes are associated with membranes and cell walls, amino acids, peroxisomes, HSPs, trehalose metabolism, redox balance, and transcription factors (Ma and Liu 2010; Mizobata et al. 2021). Interestingly, some of these gene categories showed increased expression in our study, while others decreased. Notably, several of the observed expression changes are consistent with previous reports on stress tolerance in yeast, which supports the reliability of our transcriptomic results. We anticipate that future research, including targeted quantitative PCR validation of selected genes, will more thoroughly elucidate the 2,3-BDO tolerance mechanism. The insights gained into 2,3-BDO tolerance here may also prove valuable for further enhancing tolerance to various other stresses. Additionally, this study utilized a 2,3-BDO non-producing strain with the objective of elucidating the fundamental 2,3-BDO tolerance mechanism and enhancing tolerance. Moving forward, it is anticipated that a dual approach, focusing on improving both 2,3-BDO production and tolerance, will enable the construction of engineered strains vital for sustainable industrial 2,3-BDO production. The mutagenesis technique developed in this study enables large-scale genome modifications in yeast and is expected to facilitate the development of diverse industrially valuable yeast strains in the future.

## Supplementary Information

Below is the link to the electronic supplementary material.

ESM1 (PDF.5.00 MB)

## Data Availability

All data generated or analyzed during this study are included in this published article and its supplementary information files.
