# Supplementary material for: Construction of yeast with extremely high 2,3-butanediol tolerance by introducing point and structural mutations and partial elucidation of the mechanism of 2,3-butanediol tolerance
Source: Appl Microbiol Biotechnol. 2025 Oct 16;109(1):227. doi: 10.1007/s00253-025-13626-8 (PMC12532680; doi:10.1007/s00253-025-13626-8)
Supplement: Supplementary file 1 — (PDF.5.00 MB) [file 253_2025_13626_MOESM1_ESM.pdf]

Supplementary information

Construction of yeast with extremely high 2,3-butanediol tolerance by introducing point and structural mutations and partial elucidation of the mechanism of 2,3-butanediol tolerance

Kaito Nakamura, Ryosuke Yamada, Rumi Sakaguchi, Takuya Matsumoto, and Hiroyasu Ogino

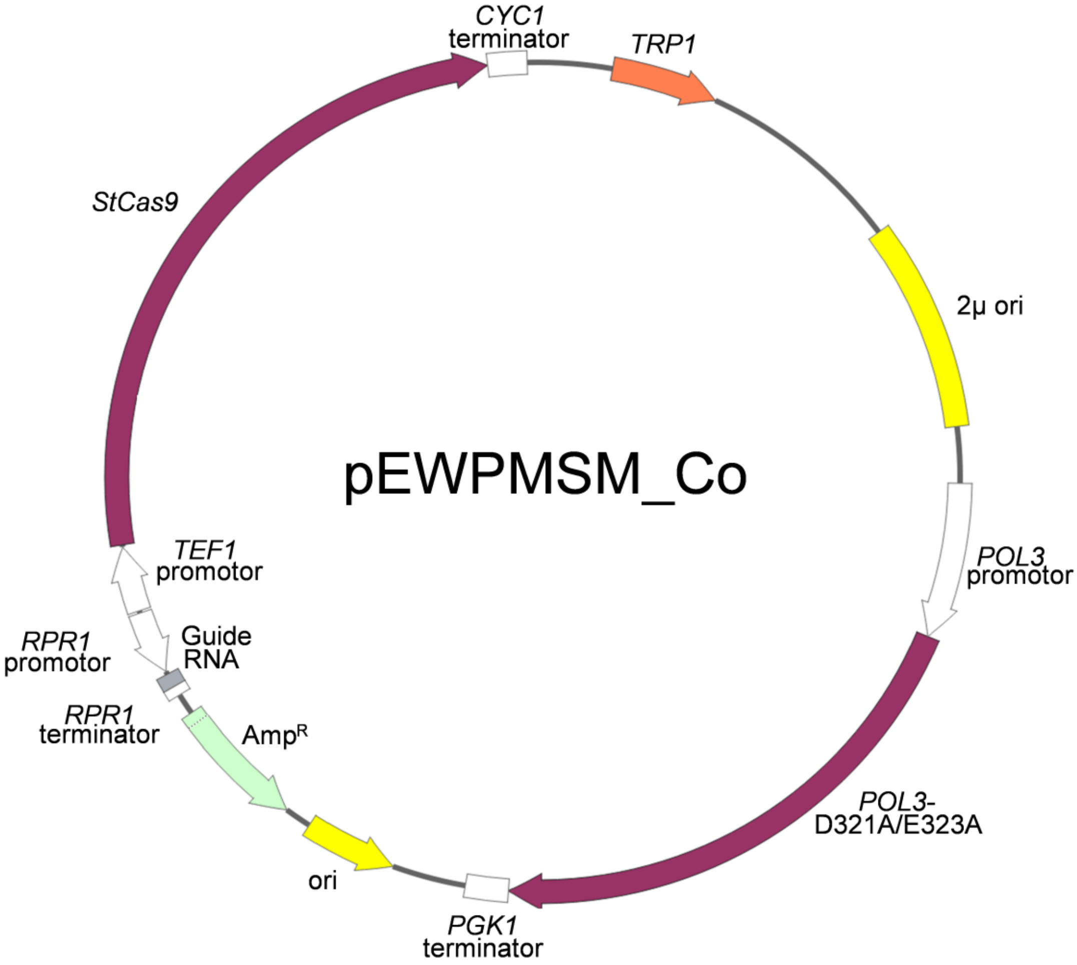

Fig. S1 Plasmid map for pEWPMSM_Co for the introduction of DNA point mutations and DNA structural mutations

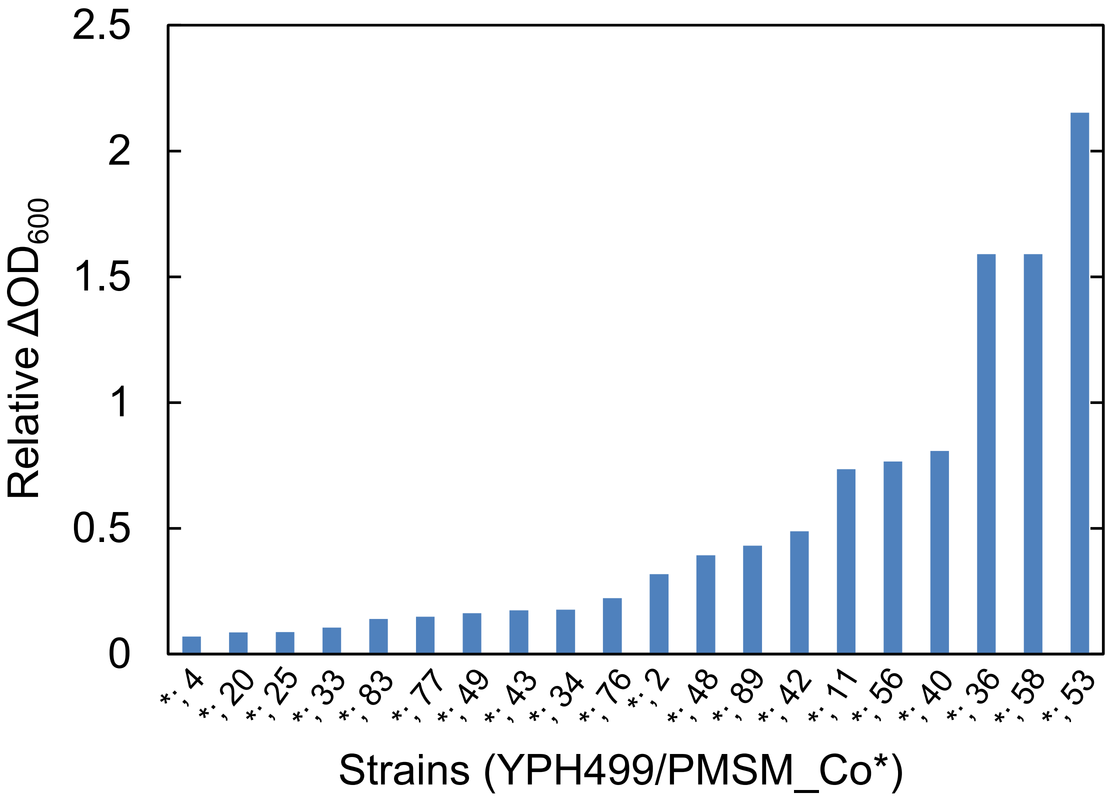

Fig. S2 Screening of 2,3-BDO-tolerant strains among 20 mutants

The Y-axis represents the relative ΔOD_{600}, which is the ΔOD_{600} in the presence of 2,3-BDO divided by the ΔOD_{600} in the absence of 2,3-BDO. The X-axis represents the top 20 mutants in ΔOD_{600} in Fig. 1. Data are presented as the average of two independent experiments. Based on the Y-axis values, the top four strains of YPH499/PMSM_CoX mutants (X: 36, 40, 53, 58) were selected.

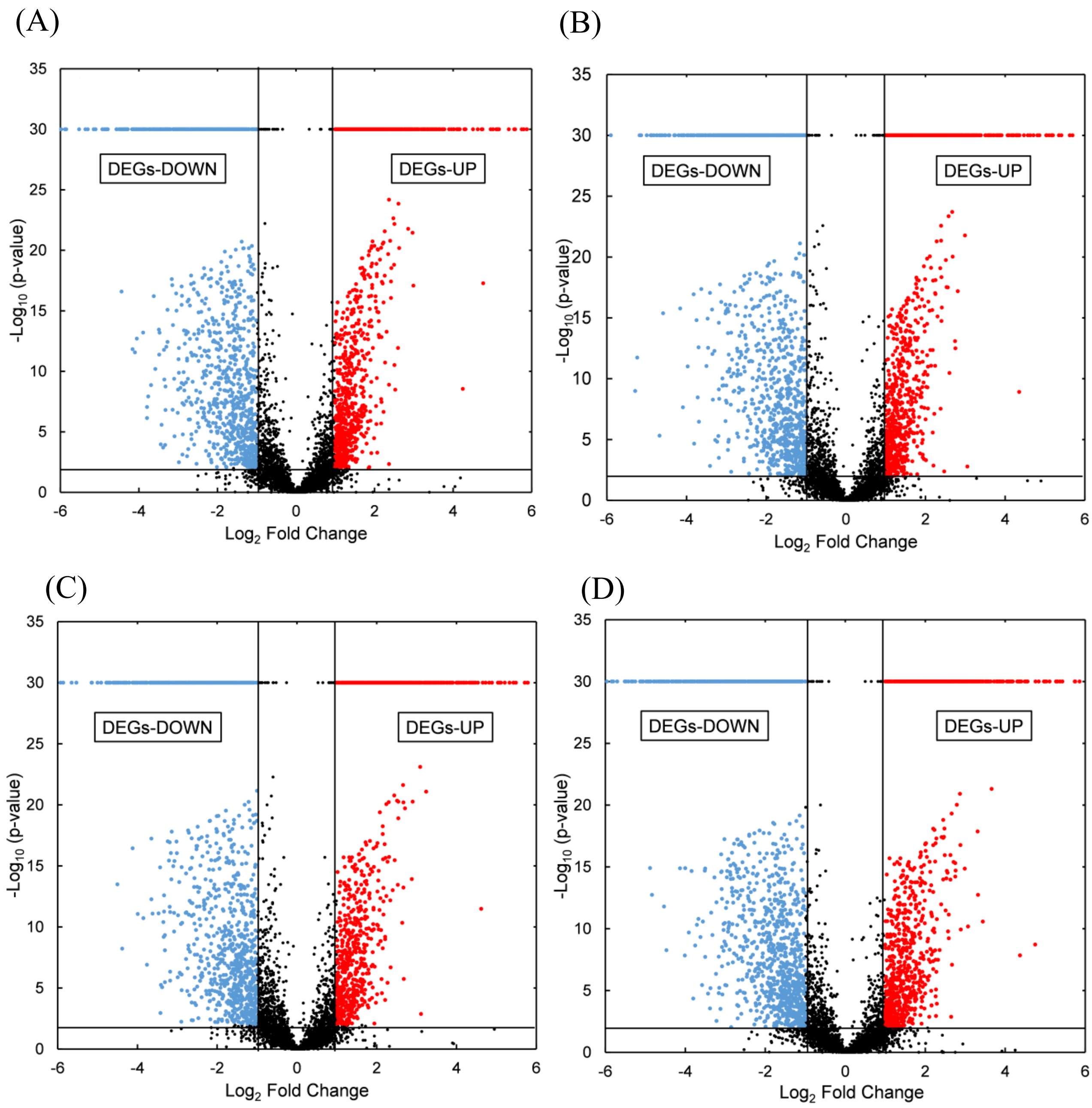

Fig. S3 Volcano plots for genes in YPH499/CoX (X: 36, 40, 53, 58).

The plot of the log2 fold change of all genes in (a) YPH499/PMSM_Co36, (b) YPH499/PMSM_Co40, (c) YPH499/PMSM_Co53, (d) YPH499/PMSM_Co58. The vertical axis indicates -log10 (p-value), and the horizontal axis indicates log2 fold change. Vertical lines represent fold changes of ±2.0 (log2 fold change = ±1.0). The horizontal line represents p-value = 0.05 (-log10 p-value is approximately 1.30). P-values were adjusted using the Benjamini–Hochberg procedure. Red and blue plot points represent DEGs and black plot points represent non-DEGs (i.e., genes whose transcription levels were not changed.)

Table S1. YPH499/Co36 gene with increased expression level due to mutation

Gene or locus tag	Log2 (fold change)	Log10 (p-value)[a)]
SPG1	11.87	30
SPS100	8.68	30
RGI2	8.17	30
SIP18	8.11	30
GRE1	7.34	30
ADY2	7.08	30
CTA1	6.78	30
FMP16	6.03	30
POT1	5.88	30
PUT1	5.80	30
CSM4	5.75	30
ACS1	5.58	30
NDE2	5.56	30
SPG4	5.42	30
ICY1	5.17	30
FOX2	5.13	30
DPC7	5.10	30
BOP2	5.02	30
HXT5	4.96	30
PAU19	4.77	17.27
GDH3	4.75	30
IZH4	4.73	30
PAI3	4.63	30
SRL4	4.51	30
MEP1	4.32	30
DAN4	4.30	30
ATP8	4.25	8.55
FAT3	4.17	30
HEF3	4.15	30
POX1	4.13	30
MSC1	4.12	30
DDR2	4.10	30
MLS1	4.02	30
PUT4	4.02	30
RTS3	4.01	30

FMP48	3.96	30
BDH2	3.90	30
ALD3	3.78	30
PCK1	3.78	30
UIP5	3.77	30
ARG82	3.76	30
HBT1	3.73	30
LSO1	3.72	30
GSC2	3.67	30
PDR10	3.66	30
JEN1	3.65	30
TLG1	3.65	30
MEP2	3.64	30
RIM4	3.63	30
PHM7	3.59	30
PRR2	3.58	30
GUT2	3.55	30
SHH4	3.54	30
VAB2	3.53	30
MND1	3.50	30
ADH2	3.48	30
PLB2	3.48	30
FMP45	3.45	30
PAL2	3.45	30
SOK2	3.44	30
MPC3	3.43	30
SFA1	3.40	30
FKS3	3.37	30
COX26	3.36	30
UMP1	3.36	30
PXA1	3.34	30
ALD4	3.33	30
CMI8	3.29	30
ARE2	3.27	30
CIS1	3.27	30
CIT2	3.26	30
CAT8	3.24	30
INO1	3.24	30

MDH2	3.24	30
ATG32	3.23	30
CRC1	3.20	30
CLD1	3.18	30
FAA4	3.13	30
RAD55	3.10	30
ENA5	3.09	30
OLE1	3.09	30
GPX1	3.08	30
SPO20	3.08	30
AGP1	3.07	30
GAP1	3.07	30
ATG1	3.04	30
ATO3	3.03	30
ATP6	3.01	30
FMP49	2.99	17.08
HVG1	2.97	21.45
ECM4	2.97	30
RAD4	2.97	30
CUP2	2.96	30
SPG3	2.96	30
DAN1	2.95	30
YIH1	2.94	30
PPQ1	2.93	30
STR3	2.93	30
DIP5	2.92	30
IRC15	2.92	30
COB	2.91	30
SHC1	2.91	30
SWI1	2.89	30
UBC6	2.89	30
ZUO1	2.89	30
STB3	2.88	30
PEX28	2.87	30
RSB1	2.87	30
SPG5	2.87	30
YAT2	2.86	21.77
GIP2	2.86	30

BLM10	2.85	30
ALT2	2.83	30
OM45	2.82	30
KHA1	2.80	30
CMC4	2.79	30
DDI1	2.79	30
RIM101	2.79	30
SEF1	2.78	30
SKS1	2.78	30
GRX6	2.77	30
ATG34	2.76	30
EAR1	2.75	30
GIS1	2.72	30
CBF1	2.70	30
ISF1	2.70	30
VHS3	2.70	30
ATG9	2.68	30
ENA2	2.68	30
RGS2	2.67	30
DOC1	2.65	30
ENA1	2.65	30
IRC13	2.65	30
YPT6	2.65	30
GTT1	2.64	30
PHO4	2.64	30
MSN2	2.63	20.18
ILT1	2.63	30
NFI1	2.63	30
NGL3	2.63	30
AST2	2.61	23.85
BYE1	2.61	30
SLZ1	2.61	30
FBP1	2.60	11.93
LGE1	2.60	30
UIP3	2.60	30
BSC2	2.59	30
MHO1	2.59	30
MCO8	2.58	30

MGA2	2.58	30
PSP1	2.57	30
JHD1	2.56	30
SPS19	2.56	30
IDP3	2.55	30
LCL1	2.55	30
PEX11	2.55	30
UBX6	2.55	30
AAC1	2.54	30
MMO1	2.53	8.48
ALD2	2.53	30
SUR1	2.53	30
APC11	2.52	30
ARO10	2.52	30
DCS2	2.52	30
ISU1	2.52	30
MEF2	2.52	30
MIT1	2.51	10.52
PTK1	2.51	22.17
EDC2	2.51	30
PTR2	2.51	30
FZO1	2.50	18.80
ECI1	2.50	30
GIS3	2.50	30
RAD51	2.50	30
SDH6	2.50	30
SNT309	2.49	30
UBX5	2.48	22.64
ICL1	2.48	30
YAP1801	2.46	30
NTH2	2.44	30
CDC27	2.43	18.53
ERV1	2.43	30
SDH2	2.43	30
DIN7	2.42	16.06
NGR1	2.42	17.42
NPR1	2.42	30
CPS1	2.41	30

KIC1	2.41	30
VPS28	2.41	30
AHC1	2.40	20.78
SPF1	2.40	30
SUF4	2.37	2.34
UGO1	2.37	8.89
CAF40	2.37	24.18
MGR3	2.37	30
RPT6	2.37	30
ATG11	2.36	30
ATO2	2.36	30
NBP35	2.36	30
ATG36	2.35	19.68
BSC4	2.35	30
DPA10	2.35	30
PET20	2.35	30
PFK26	2.35	30
CTF19	2.34	30
FMP40	2.34	30
MIX17	2.34	30
GPG1	2.33	30
HHO1	2.32	30
SUM1	2.32	30
YPT31	2.31	13.27
KNS1	2.31	30
HUA1	2.30	30
RCN2	2.30	30
SCS7	2.30	30
CCL1	2.29	30
SIP2	2.29	30
SND1	2.29	30
BI4	2.28	30
OLI1	2.28	30
PIL1	2.28	30
VHC1	2.27	10.72
YTH1	2.27	30
HAL9	2.26	17.90
TFC4	2.26	21.56

GDH2	2.26	30
UGA3	2.26	30
DBP1	2.25	30
HAA1	2.24	30
SFC1	2.23	6.65
SPC42	2.23	15.69
NUD1	2.23	20.64
ARO9	2.23	30
MUB1	2.23	30
PMP3	2.23	30
DAT1	2.22	30
OPY1	2.21	17.25
VID27	2.21	20.54
CCC1	2.21	30
HFD1	2.21	30
NDL1	2.21	30
RIB1	2.20	30
SSM4	2.20	30
OM14	2.19	17.39
SLM3	2.19	30
MIN10	2.18	7.46
RKM5	2.18	12.83
BRE4	2.18	30
MOH1	2.18	30
SSH4	2.18	30
CMR3	2.17	5.69
RNR3	2.17	7.70
WBP1	2.17	20.20
FMS1	2.17	30
GPT2	2.17	30
ICT1	2.17	30
MAG1	2.17	30
PRB1	2.17	30
WIP1	2.17	30
RTN2	2.16	30
OTU1	2.15	30
RPM1	2.15	30
SNC2	2.15	30

SKN7	2.14	19.22
AFG3	2.14	30
CRF1	2.14	30
TSC11	2.12	12.82
GPA2	2.12	30
PDR1	2.12	30
UBC4	2.12	30
VHT1	2.12	30
GIP1	2.11	13.01
PEX18	2.11	14.14
LPX1	2.11	30
YSH1	2.10	14.87
CPR1	2.10	30
TBS1	2.09	20.05
CCS1	2.09	30
CIT3	2.09	30
CSR2	2.09	30
REH1	2.09	30
SYG1	2.09	30
PRP31	2.08	20.09
DBP2	2.08	30
FMP52	2.08	30
GET3	2.08	30
HHF1	2.08	30
RRT7	2.07	3.28
TDA11	2.07	14.92
ATG5	2.07	30
BDF2	2.07	30
RTG1	2.07	30
PEX9	2.06	9.44
MNC1	2.06	17.84
MOT2	2.06	18.05
BCP1	2.06	30
GRX4	2.06	30
MET8	2.05	5.64
GPI10	2.05	18.88
ATH1	2.05	30
AVT3	2.05	30

FAA1	2.05	30
CIP1	2.04	7.13
CAF120	2.04	15.24
GFD1	2.04	30
SNQ2	2.04	30
PGD1	2.03	15.79
PEX22	2.03	16.20
AMS1	2.03	30
ATG39	2.03	30
RAD54	2.03	30
PEX15	2.02	30
SPT2	2.02	30
SUE1	2.02	30
URA2	2.02	30
DIA1	2.01	30
FUB1	2.01	30
MSC3	2.01	30
RPN3	2.01	30
NRD1	2.00	20.36
GLO1	2.00	30
IRC20	2.00	30
MPE1	2.00	30
PEX3	2.00	30
RPN8	2.00	30
TPI1	2.00	30
GTO3	1.99	4.47
CSC1	1.99	30
GYP7	1.99	30
ATG15	1.98	30
HUL5	1.98	30
RRT1	1.97	30
RRT15	1.97	30
SNX4	1.97	30
HYM1	1.96	11.93
MET30	1.96	30
RFA3	1.96	30
COX23	1.95	20.73
CDC31	1.95	30

HRT1	1.95	30
NAB3	1.95	30
INO2	1.94	16.88
DAS1	1.94	17.14
ATG4	1.94	18.91
HSF1	1.94	30
UNG1	1.94	30
PRP18	1.93	13.70
RAP1	1.93	20.34
GLN1	1.93	30
NBL1	1.93	30
LEE1	1.92	3.24
CTK3	1.92	9.12
RAD14	1.92	16.24
GNP1	1.92	18.67
MVP1	1.92	20.18
RTC6	1.92	30
SUR2	1.92	30
TOR1	1.92	30
RSA1	1.91	14.20
BPT1	1.91	30
FAS1	1.91	30
GAL83	1.91	30
RFA1	1.91	30
TGL4	1.91	30
FMP23	1.90	5.92
PIG2	1.90	7.92
RRG1	1.90	10.94
GPN2	1.90	30
MPO1	1.90	30
NAR1	1.90	30
RCF3	1.89	6.67
MDM34	1.89	17.78
HYR1	1.89	30
NUM1	1.89	30
RPT2	1.89	30
HMS1	1.88	15.15
PUT2	1.88	30

SCH9	1.88	30
UBC9	1.88	30
CWC23	1.87	2.08
RTS2	1.87	13.73
MPS3	1.87	14.08
CST6	1.87	19.93
KRE1	1.87	30
MRPL16	1.87	30
PFU1	1.87	30
PSH1	1.87	30
ALP1	1.86	16.18
ATG14	1.85	2.00
EMP65	1.85	11.51
NNK1	1.85	19.59
SNA3	1.85	30
SPO24	1.85	30
SSL2	1.85	30
TFS1	1.85	30
UME1	1.85	30
ISR1	1.84	5.25
SNF11	1.84	12.64
SHH3	1.84	17.98
COX7	1.84	30
CDC26	1.83	5.84
ATG8	1.83	30
BUD23	1.83	30
GRR1	1.83	30
BEM1	1.82	13.06
APE3	1.82	30
MPD2	1.82	30
TMH11	1.82	30
SWC5	1.81	11.42
DEF1	1.81	30
TEX1	1.80	4.02
SFL1	1.80	9.66
HXT4	1.80	30
MRPL28	1.80	30
SHE10	1.80	30

HOT1	1.79	4.92
PEX30	1.79	30
WHI4	1.78	6.75
DPB4	1.78	30
RFA2	1.78	30
SNA4	1.77	7.08
BXI1	1.77	30
SUT2	1.76	10.07
GLC3	1.76	30
PUT3	1.76	30
NNF2	1.75	11.05
KEL1	1.75	12.69
HIT1	1.75	13.87
MAF1	1.75	17.65
CAR2	1.75	18.35
ACC1	1.75	30
APJ1	1.75	30
PMC1	1.75	30
SKP1	1.75	30
SEN34	1.74	4.60
ARA1	1.74	17.89
CWC24	1.74	30
VAS1	1.73	6.35
VOA1	1.73	6.53
RTG3	1.73	6.90
YSA1	1.73	8.34
CDC23	1.73	9.37
PRE5	1.73	30
24-May	1.72	7.18
UTH1	1.72	7.90
MED7	1.72	9.33
RRT6	1.72	9.46
GSH1	1.72	17.89
TGL5	1.72	18.15
DDP1	1.72	30
PCP1	1.72	30
PRM3	1.71	4.70
FAA2	1.71	11.28

TUM1	1.71	13.16
COX1	1.71	30
TOF1	1.70	7.09
SAF1	1.70	8.64
ATG26	1.70	30
MAP1	1.70	30
PNT1	1.69	3.34
FHN1	1.69	6.23
NTO1	1.69	7.16
SLX8	1.69	18.56
RNA14	1.69	19.34
ASG1	1.68	8.16
GTO1	1.68	9.22
SLO1	1.68	11.70
RSN1	1.68	15.31
SRC1	1.68	18.06
CAB4	1.68	19.34
GID8	1.68	30
GLT1	1.68	30
MRX8	1.67	6.65
GAT2	1.67	6.97
SEN2	1.67	10.13
SDP1	1.67	12.95
LAP3	1.67	30
ZTA1	1.67	30
SNC1	1.66	4.83
BSC1	1.66	5.41
SPP381	1.66	5.84
FYV6	1.66	16.62
SYC1	1.65	6.36
ADY4	1.65	8.71
WWM1	1.65	13.42
STE24	1.65	14.91
HUR1	1.65	30
RPT1	1.65	30
ECM10	1.64	6.29
FZF1	1.64	16.19
AIM9	1.64	18.52

MPC1	1.64	30
PCT1	1.64	30
SHP1	1.64	30
TFA1	1.64	30
VPS3	1.63	16.22
AFT2	1.63	30
AI5_ALPHA	1.63	30
SPT15	1.63	30
CDC53	1.62	30
GAC1	1.62	30
KGD2	1.62	30
PDH1	1.62	30
GPI2	1.61	6.79
MCM1	1.61	10.84
ARG80	1.61	30
GLC8	1.61	30
PHB2	1.61	30
RPS27A	1.61	30
PHO92	1.60	5.21
ROY1	1.60	10.58
CFD1	1.60	14.23
MLO1	1.60	15.28
ASK1	1.60	30
ISA1	1.60	30
SPP41	1.59	5.29
DAL80	1.59	5.75
MSG5	1.59	5.99
SAN1	1.59	13.22
RAD16	1.59	13.92
AI4	1.59	30
CCT3	1.59	30
HST1	1.59	30
TPK2	1.59	30
CHK1	1.58	5.54
YET3	1.58	11.49
FAP1	1.58	16.09
COX3	1.58	30
LYS20	1.58	30

WTM2	1.58	30
GOS1	1.57	10.20
VPS60	1.57	15.99
CDC34	1.57	30
GLO4	1.57	30
ISY1	1.57	30
MDH3	1.57	30
MIN8	1.57	30
PEP4	1.57	30
YNK1	1.57	30
GZF3	1.56	2.74
SUF2	1.56	4.39
RBL2	1.56	5.17
TIM17	1.56	6.49
PEX5	1.56	11.66
DNF1	1.56	30
ORM2	1.56	30
HEL1	1.55	5.93
QDR1	1.55	6.25
ECM2	1.55	10.28
BRE1	1.55	15.71
15S_RRNA	1.55	16.67
IMP2'	1.55	30
MET4	1.55	30
OAZ1	1.55	30
PGA2	1.55	30
RPT4	1.55	30
PDC6	1.54	3.73
SEN54	1.54	5.86
RRD1	1.54	12.52
VID28	1.54	13.94
CRP1	1.54	30
PRE10	1.54	30
STE11	1.54	30
UFD2	1.54	30
ASG7	1.53	2.51
RCI50	1.53	7.99
HTZ1	1.53	11.50

THI7	1.53	16.20
ECM21	1.53	17.21
ESA1	1.53	30
TMA108	1.53	30
HOT13	1.52	2.54
MCO32	1.52	3.62
MNT4	1.52	3.79
PTI1	1.52	4.17
PDR8	1.52	7.33
RSF1	1.52	8.84
SDH8	1.52	10.16
CCT8	1.52	30
TPS3	1.52	30
HIR2	1.51	2.78
LUG1	1.51	4.42
ARA2	1.51	4.97
YUH1	1.51	5.35
PDS1	1.51	6.05
NCB2	1.51	11.77
RRT8	1.51	12.29
NUP2	1.51	30
PMR1	1.51	30
CDC123	1.50	4.49
ELA1	1.50	6.22
SHR5	1.50	7.35
ELP4	1.50	12.86
BOI2	1.50	13.23
UBC7	1.50	15.92
HUB1	1.50	16.75
FET5	1.50	30
MNN5	1.50	30
TPA1	1.50	30
ROG1	1.49	6.02
SGT1	1.49	9.87
AI3	1.49	30
AIM46	1.49	30
DLD3	1.49	30
GIM4	1.49	30

AZF1	1.48	5.96
VPS73	1.48	6.59
PHO2	1.48	8.31
AIM19	1.48	8.49
NFU1	1.48	8.60
RRN3	1.48	10.12
PLB3	1.48	11.38
GSM1	1.48	12.53
AI2	1.48	30
FMP46	1.48	30
LSP1	1.48	30
MPM1	1.48	30
RVS161	1.48	30
SEM1	1.48	30
RAD30	1.47	5.17
FEX2	1.47	5.33
RMD5	1.47	13.73
RSC2	1.47	14.87
MKK2	1.47	15.13
RAD23	1.47	30
TYR1	1.47	30
MSA1	1.46	4.11
INP54	1.45	3.45
IZH1	1.45	5.52
ATG21	1.45	6.44
PBA1	1.45	6.46
TFB1	1.45	16.70
LDB7	1.45	30
OPI1	1.45	30
SOL2	1.45	30
SSU72	1.44	2.68
HAP2	1.44	3.51
MTG2	1.44	4.96
CDC16	1.44	5.60
ICL2	1.44	6.95
CDH1	1.44	7.07
VHS1	1.44	8.11
COX16	1.44	9.83

NAS6	1.44	11.21
SMB1	1.44	11.92
CCT5	1.44	30
CCT6	1.44	30
ROX3	1.44	30
ECM23	1.43	2.99
OAF1	1.43	4.61
MNN14	1.43	7.24
PCL8	1.43	7.37
PXP1	1.43	8.59
SWT21	1.43	9.66
MAM3	1.43	12.80
QNS1	1.43	13.71
PET100	1.43	14.78
DSC2	1.43	15.07
BI2	1.43	30
SWF1	1.42	5.59
DPC25	1.42	5.92
ASR1	1.42	10.38
MCH1	1.42	12.78
TVP23	1.42	14.72
ARB1	1.42	30
FMP10	1.42	30
SAP155	1.42	30
SCEI	1.42	30
SLG1	1.42	30
XDJ1	1.41	3.41
HXT9	1.41	5.57
MDV1	1.41	5.60
NAM9	1.41	9.75
ORM1	1.41	10.61
CDD1	1.41	11.50
ESL2	1.41	12.04
MSP1	1.41	14.72
RCR1	1.41	14.79
CUZ1	1.41	30
SPO19	1.40	2.82
ABM1	1.40	3.39

CRZ1	1.40	3.84
HPA2	1.40	4.45
PXA2	1.40	5.16
RMD1	1.40	5.39
PTC1	1.40	10.56
COX2	1.40	30
QCR6	1.40	30
UBP1	1.40	30
SEA4	1.39	2.98
CWC21	1.39	3.42
VMA1	1.39	4.82
GON7	1.39	8.10
MXR1	1.39	8.99
COQ4	1.39	10.63
ATG20	1.39	10.67
PKH2	1.39	11.21
NQM1	1.39	30
HTA2	1.38	5.68
YSC83	1.38	6.46
TAF13	1.38	7.05
RPC31	1.38	9.25
ERC1	1.38	15.69
MIC26	1.38	30
SPT5	1.38	30
UBR2	1.38	30
IBA57	1.37	2.85
RIT1	1.37	4.10
GPI19	1.37	4.37
UBX2	1.37	4.87
PBI2	1.37	16.52
ADR1	1.37	30
RNH70	1.37	30
IRA1	1.36	4.61
ECM27	1.36	4.74
RRD2	1.36	7.80
VTC2	1.36	8.30
RQC1	1.36	10.01
INO4	1.36	10.75

RAD52	1.36	11.01
PEX19	1.36	13.14
HSV2	1.36	16.50
CDC37	1.36	30
NPC2	1.36	30
PGA3	1.36	30
PYC1	1.36	30
HXT11	1.35	5.13
NUT2	1.35	6.40
YPI1	1.35	6.67
RAD1	1.35	9.81
IES6	1.35	30
PRC1	1.35	30
RPN13	1.35	30
YRA2	1.35	30
CBF2	1.34	2.12
SNG1	1.34	6.25
SPR6	1.34	6.25
TEM1	1.34	6.95
FHL1	1.34	7.04
BI3	1.34	10.32
COA4	1.33	3.96
CAB2	1.33	4.68
FYV8	1.33	9.07
MRS6	1.33	30
RPN1	1.33	30
SKN1	1.33	30
BIG1	1.32	2.68
SHU2	1.32	3.47
PET130	1.32	4.04
EAF3	1.32	8.09
IOC4	1.32	8.57
SNF3	1.32	11.03
PDR12	1.32	16.19
RPT3	1.32	30
YTA6	1.32	30
RGD1	1.31	3.24
PAH1	1.31	5.61

COQ21	1.31	6.66
VIK1	1.31	6.95
YIP4	1.31	8.36
MFB1	1.31	9.25
VPS36	1.31	10.62
FLC2	1.31	13.10
SCS2	1.31	14.30
NYV1	1.31	14.42
COA6	1.31	15.14
OPY2	1.31	30
SSD1	1.31	30
SAW1	1.30	2.84
AEP3	1.30	2.99
RAS1	1.30	3.25
PAN6	1.30	6.55
HTL1	1.30	6.57
ATG19	1.30	6.80
APP1	1.30	9.63
MIN7	1.30	10.68
URK1	1.30	12.45
MUM2	1.29	2.96
ARK1	1.29	3.52
LDO16	1.29	4.09
IBD2	1.29	4.34
OSW7	1.29	6.51
NPL4	1.29	7.25
UCC1	1.29	7.26
NUP60	1.28	2.21
LCD1	1.28	2.48
SLD2	1.28	2.81
RTR1	1.28	3.20
PIB2	1.28	3.87
TFB4	1.28	3.92
PEX31	1.28	4.19
TFB5	1.28	4.94
CSS3	1.28	6.91
LPL1	1.28	30
PSO2	1.27	2.42

KRE5	1.27	2.67
RPI1	1.27	2.97
ASF2	1.27	3.10
STE7	1.27	3.53
SNF1	1.27	3.62
BAG7	1.27	5.28
MRK1	1.27	5.82
DIF1	1.27	5.97
YVC1	1.27	6.92
RSC4	1.27	7.42
COX11	1.27	7.44
VPS25	1.27	7.61
TAF14	1.27	7.88
TAF4	1.27	8.16
TOA2	1.27	9.84
INH1	1.27	30
RPS30B	1.27	30
SCD5	1.26	4.21
UBR1	1.26	5.67
PCL10	1.26	7.60
CDC40	1.26	8.64
SAS5	1.26	10.26
SPT16	1.26	11.30
TAF1	1.26	11.42
IRA2	1.26	11.63
SDD3	1.26	13.16
PRE2	1.26	30
PUP2	1.26	30
JID1	1.25	4.02
ATP25	1.25	4.69
RPL18B	1.25	6.43
MTC6	1.25	6.83
DBP8	1.25	7.42
RSM10	1.25	10.87
YPS3	1.25	11.98
TAF12	1.25	13.66
CHZ1	1.25	30
COX15	1.25	30

RTK1	1.25	30
PDR3	1.24	2.11
GET2	1.24	2.69
HSM3	1.24	2.85
IAI11	1.24	3.25
ZRT1	1.24	4.52
VPS63	1.24	10.35
GDH1	1.24	30
HPF1	1.24	30
COS9	1.23	3.03
MND2	1.23	3.67
UBX4	1.23	4.39
NHX1	1.23	5.74
ASK10	1.23	8.49
TOM71	1.23	9.26
IWR1	1.23	10.34
DSK2	1.23	30
KTR1	1.23	30
PRE3	1.23	30
ISC10	1.22	2.16
RAM1	1.22	2.47
STP4	1.22	2.84
RTT105	1.22	3.01
SDS23	1.22	3.95
INA17	1.22	6.13
UBP7	1.22	13.00
INP1	1.21	2.37
EKI1	1.21	2.43
MCO76	1.21	2.51
FKH2	1.21	2.59
POG1	1.21	3.08
SPT10	1.21	3.44
KEL2	1.21	3.86
SCC4	1.21	4.61
PSF2	1.21	4.99
TFB3	1.21	5.36
RSM22	1.21	7.36
RPO31	1.21	8.22

MRPL10	1.21	9.50
CTR2	1.21	11.08
SEC17	1.21	11.39
BDF1	1.21	14.85
BAP2	1.21	30
SND3	1.21	30
GEP5	1.20	2.17
YMD8	1.20	2.29
DSL1	1.20	3.12
RPD3	1.20	5.31
YFH7	1.20	6.51
VBA2	1.20	6.85
FIS1	1.20	30
KTR4	1.20	30
TIF6	1.20	30
FUN19	1.19	2.15
TIM13	1.19	2.31
LEU3	1.19	2.85
ALG14	1.19	3.04
AQY1	1.19	3.14
ATG40	1.19	4.82
MRX9	1.19	4.93
SWR1	1.19	7.06
CKB2	1.19	8.40
RUB1	1.19	11.18
CCT2	1.19	30
DPL1	1.19	30
NFS1	1.19	30
PRE1	1.19	30
DFR1	1.18	2.49
MRP2	1.18	3.89
ACK1	1.18	4.16
RPH1	1.18	5.88
SPT6	1.18	9.77
FPK1	1.18	12.69
STP3	1.18	14.38
AI1	1.18	30
SDD4	1.17	3.05

PCC1	1.17	3.60
TNA1	1.17	5.21
YAP6	1.17	8.06
MEH1	1.17	8.64
UFD1	1.17	13.42
BMH1	1.17	30
TCP1	1.17	30
CPT1	1.16	4.33
EFM4	1.16	4.83
RER1	1.16	6.24
SRP54	1.16	13.42
ASN2	1.16	30
RPN7	1.16	30
NCA3	1.15	2.06
MOD5	1.15	2.26
INP52	1.15	3.12
SLF1	1.15	3.59
UME6	1.15	4.05
DUG3	1.15	4.32
GPI8	1.15	5.20
LRE1	1.15	5.70
HRT3	1.15	5.83
FYV10	1.15	7.65
MSN5	1.15	12.64
SNP1	1.14	2.03
TAD2	1.14	2.72
DPB11	1.14	2.94
VHS2	1.14	3.67
STP1	1.14	3.91
SLY1	1.14	4.40
MSN1	1.14	6.71
AI5_BETA	1.14	30
MIA40	1.14	30
NUR1	1.13	3.91
HRQ1	1.13	4.57
THP1	1.13	4.82
YPT35	1.13	5.12
HEM2	1.13	5.15

NIS1	1.13	5.24
SSK2	1.13	6.37
SNA2	1.13	10.65
MIM1	1.13	12.98
COA3	1.13	30
EMP46	1.12	3.24
CEP3	1.12	3.48
CSS2	1.12	3.76
PRY2	1.12	4.32
MGM1	1.12	4.62
MIH1	1.12	5.47
SEG1	1.12	5.91
UGA2	1.12	5.92
APL1	1.12	5.94
STH1	1.12	6.04
RDS2	1.12	6.81
MRPL51	1.12	7.30
MAG2	1.12	8.04
FRQ1	1.12	9.82
UTR2	1.12	10.12
PUP1	1.12	11.11
MCO10	1.12	12.24
LPP1	1.11	2.24
PPH3	1.11	2.54
RAD50	1.11	2.74
PLC1	1.11	3.31
TFC8	1.11	3.71
BUD16	1.11	4.01
HIR1	1.11	4.06
SPP1	1.11	4.10
SDS3	1.11	4.43
MATALPHA2	1.11	4.64
MHP1	1.11	4.97
GCR2	1.11	5.99
VPS71	1.11	7.25
PRP4	1.10	2.91
RNQ1	1.10	4.05
MLH3	1.10	4.85

RRN10	1.10	4.86
PCS60	1.10	6.11
SLM1	1.10	6.60
UBP14	1.10	6.99
MDH1	1.10	30
PRE9	1.10	30
RPS27B	1.10	30
PCI8	1.09	2.95
CAT2	1.09	3.39
SWM2	1.09	3.46
MSR1	1.09	4.05
SLM4	1.09	4.62
SOM1	1.09	6.37
PAN2	1.09	8.02
NCR1	1.09	8.84
PRE4	1.09	30
VAC14	1.08	2.29
REF2	1.08	2.51
VID30	1.08	2.78
NDC80	1.08	3.22
MET28	1.08	4.04
PEX13	1.08	4.27
RAD5	1.08	4.30
LEA1	1.08	4.82
MSC6	1.08	4.98
TMT1	1.08	5.05
YAP5	1.08	5.54
PSK1	1.08	8.51
MCT1	1.07	2.33
NSL1	1.07	3.17
ASE1	1.07	3.28
RAD26	1.07	3.32
PEX10	1.07	3.99
PCL7	1.07	5.04
CTK1	1.07	5.92
PAR32	1.07	6.82
NST1	1.07	6.97
GRE2	1.07	8.36

CSG2	1.07	9.86
FPS1	1.07	12.80
RRN5	1.06	2.21
DPI35	1.06	3.15
UGA1	1.06	6.35
FTH1	1.06	9.08
RNR2	1.06	15.70
21S_RRNA	1.06	30
TIP41	1.05	2.15
MRX10	1.05	2.45
MNE1	1.05	2.66
RAD57	1.05	3.63
MKK1	1.05	5.22
RRP46	1.05	5.25
NEO1	1.05	5.47
LAC1	1.05	7.06
NHP6B	1.05	7.64
MRPS18	1.05	10.11
MDY2	1.04	2.18
MNN1	1.04	4.14
DIC1	1.04	4.81
ITT1	1.04	5.30
KTI11	1.04	5.93
ACO1	1.04	12.71
PRE7	1.04	13.11
STF1	1.04	14.92
FAS2	1.04	30
MED2	1.03	2.77
PPG1	1.03	3.70
MFG1	1.03	4.07
BUR6	1.03	4.30
YIP1	1.03	4.43
CEG1	1.03	4.69
LSM5	1.03	5.16
SGV1	1.03	6.21
RUP1	1.03	6.33
RPB4	1.03	11.13
VPS24	1.03	14.74

RHO1	1.03	30
RPN6	1.03	30
GRX3	1.02	5.20
ARP8	1.02	6.04
INO80	1.02	13.05
TDA5	1.01	2.38
HFL1	1.01	2.90
RAD3	1.01	2.97
PSY2	1.01	3.98
ERV2	1.01	4.04
AVT6	1.01	5.36
CRM1	1.01	14.13
PUP3	1.01	30
RPB2	1.01	30
SNU56	1.00	2.14
NRT1	1.00	3.41
SWC4	1.00	3.69
UFO1	1.00	3.70
PIB1	1.00	4.24
RCI37	1.00	6.17
POB3	1.00	7.39
SSU1	1.00	7.62
TIM22	1.00	8.37
SUA7	1.00	12.36
ARF2	1.00	30

a) -Log10 (p-value) greater than 30 is indicated as 30.

Table S2. YPH499/Co36 gene with decreased expression level due to mutation

Gene or locus tag	Log2(fold change)	Log10(p-value)[a)]
RGI1	-8.28	30
CUP1-1	-8.24	30
CUP1-2	-8.12	30
HSP30	-7.23	30
AAD3	-6.82	30
HXT3	-6.43	30
PDC1	-6.39	30
ULP1	-6.09	30
BRP1	-5.98	30
YPS7	-5.88	30
OPI10	-5.85	30
IRC7	-5.52	30
ARI1	-5.36	30
HSP150	-5.30	30
SAP4	-5.15	30
REE1	-5.10	30
ENO2	-4.93	30
THI13	-4.88	30
AIM17	-4.85	30
MRX15	-4.82	30
CDC3	-4.80	30
COS5	-4.79	30
ALT1	-4.57	30
HXK2	-4.56	30
SSA4	-4.50	30
CYB5	-4.46	30
SUR7	-4.46	30
COS2	-4.45	30
DCD1	-4.44	16.59
FIT2	-4.41	30
MVD1	-4.41	30
SUC2	-4.38	30
FAR1	-4.37	30
DSF1	-4.36	30
COS3	-4.35	30
CIR1	-4.33	30

EXG1	-4.32	30
RDS1	-4.32	30
MLO50	-4.31	30
TKL2	-4.31	30
UBP12	-4.30	30
RAX2	-4.28	30
TEN1	-4.17	30
DUT1	-4.16	11.83
CDC10	-4.16	30
XPT1	-4.16	30
KEL3	-4.13	30
BFR1	-4.11	30
KIN3	-4.10	11.56
HEM25	-4.08	30
CIS3	-4.07	30
HXK1	-4.07	30
CYS3	-4.06	30
CLB2	-4.05	12.70
CRN1	-4.03	30
PPZ1	-4.02	30
PNP1	-4.01	30
CYK3	-3.97	30
EGO4	-3.95	30
ERG4	-3.91	30
HBN1	-3.89	13.20
MKC7	-3.89	30
TOK1	-3.89	30
CPR5	-3.87	30
EXG2	-3.87	30
PIR3	-3.86	30
ARO7	-3.85	30
MME1	-3.85	30
LYS9	-3.84	30
RBH2	-3.80	30
SWI5	-3.79	6.14
AAC3	-3.79	7.05
GAT4	-3.76	7.92
GOR1	-3.75	30

MIC60	-3.75	30
DSE4	-3.74	30
MAN2	-3.73	30
DAL1	-3.70	30
ALK1	-3.69	8.86
ABP1	-3.69	30
MAL32	-3.68	30
SAM1	-3.64	30
BTN2	-3.63	30
DIA2	-3.62	16.21
HXT13	-3.62	30
AFR1	-3.61	30
MAL12	-3.61	30
NCW1	-3.60	30
MYO4	-3.59	30
CLB1	-3.58	10.19
YRF1-8	-3.58	30
DOG2	-3.55	30
TPM2	-3.55	30
COQ2	-3.54	30
FRS1	-3.52	30
SPO74	-3.51	30
GRX8	-3.49	13.44
CLN2	-3.48	11.38
NTG1	-3.47	13.19
IMD2	-3.47	30
SCW4	-3.47	30
SUN4	-3.47	30
TAL1	-3.47	30
MIS1	-3.46	30
NUP188	-3.45	30
TIP20	-3.45	30
PFS1	-3.44	3.96
AGC1	-3.43	30
HXT14	-3.42	5.58
MSF1	-3.42	30
SOP4	-3.40	30
PAU15	-3.38	3.70

GCV1	-3.37	30
PER33	-3.37	30
KAR1	-3.36	11.77
AMN1	-3.36	30
FET4	-3.36	30
MST1	-3.35	9.32
RAI1	-3.35	30
YPK3	-3.34	30
THI12	-3.33	3.91
IMA2	-3.33	30
RME1	-3.33	30
VTH2	-3.33	30
SPO77	-3.32	6.80
MCM16	-3.32	8.74
YRF1-7	-3.32	30
AHP1	-3.31	30
FRE3	-3.30	12.02
PHR1	-3.30	30
SRX1	-3.30	30
VTI1	-3.29	30
QDR2	-3.28	30
YRF1-6	-3.28	30
PAU23	-3.27	12.43
BUD4	-3.27	15.08
VMS1	-3.27	30
CTR1	-3.25	30
SIA1	-3.25	30
ERG26	-3.24	30
SSA2	-3.24	30
TUB4	-3.24	30
KAE1	-3.23	30
SAM4	-3.22	30
FKS1	-3.20	30
PAN5	-3.20	30
BDH1	-3.19	30
YRF1-4	-3.16	17.62
YRF1-3	-3.15	17.12
SAE3	-3.14	6.82

YET1	-3.13	13.82
SKM1	-3.13	30
FPR4	-3.12	30
ANB1	-3.11	30
RTC3	-3.11	30
NUC1	-3.10	9.38
ACP1	-3.10	30
ARO8	-3.10	30
ERD2	-3.10	30
LDS1	-3.09	4.63
BNA3	-3.09	30
PSD2	-3.09	30
RTC4	-3.09	30
SER2	-3.08	30
SKT5	-3.08	30
SIL1	-3.07	30
SSA1	-3.06	30
UTR4	-3.06	30
YRM1	-3.06	30
GFD2	-3.05	16.43
STE4	-3.05	30
RRI2	-3.04	15.01
YME1	-3.03	30
SIM1	-3.02	30
SMC2	-3.02	30
TGL2	-3.02	30
SKG6	-3.01	6.57
ERG6	-3.01	30
RRG7	-3.01	30
SRD1	-3.01	30
YIA6	-3.00	8.49
YRF1-2	-2.99	11.00
ADH7	-2.98	2.48
WSC4	-2.98	9.88
SRL1	-2.98	11.93
GRE3	-2.98	30
HOM3	-2.98	30
PRO1	-2.98	30

RPS17B	-2.97	30
YPS1	-2.96	30
VMA3	-2.94	8.63
LYS1	-2.94	30
SCP160	-2.94	30
PRM5	-2.93	30
GDB1	-2.92	30
GPH1	-2.92	30
CTF13	-2.91	9.04
OSH6	-2.91	15.49
PET8	-2.91	17.01
ERG20	-2.91	30
SRM1	-2.91	30
SPH1	-2.90	16.24
GSH2	-2.90	30
MDM10	-2.89	3.12
EGH1	-2.89	7.37
ARC40	-2.89	30
YRF1-5	-2.89	30
MEI4	-2.88	4.78
PWP1	-2.88	30
STE5	-2.88	30
TTI2	-2.86	8.92
EGT2	-2.86	9.93
SAC1	-2.86	30
CLN1	-2.85	7.23
MPH3	-2.85	13.38
ATX2	-2.85	30
RED1	-2.85	30
APC5	-2.84	8.42
DAS2	-2.83	12.28
EHT1	-2.83	30
ERG27	-2.83	30
IMD4	-2.83	30
TPT1	-2.83	30
ELO3	-2.81	30
IMA5	-2.80	9.08
GNA1	-2.80	13.05

AXL2	-2.80	13.34
MST27	-2.79	7.54
OYE2	-2.79	30
RPS22A	-2.79	30
RPL18A	-2.78	30
GAT3	-2.77	2.98
SOL3	-2.77	10.88
CSI2	-2.77	11.05
ADH1	-2.77	30
BIO5	-2.77	30
MRS3	-2.77	30
SPT14	-2.77	30
SCO2	-2.76	15.77
SES1	-2.76	30
SKG1	-2.76	30
LOT6	-2.75	9.38
RPN14	-2.75	13.23
HIS2	-2.75	30
LYS4	-2.75	30
SPO13	-2.74	3.57
CRG1	-2.74	30
GCN1	-2.74	30
BAR1	-2.73	6.01
MCD1	-2.73	7.83
GAS3	-2.73	15.92
ECM31	-2.70	6.02
MYO1	-2.70	12.68
URM1	-2.70	15.43
ADA2	-2.70	30
CHS3	-2.70	30
EGD2	-2.70	30
EMP24	-2.70	30
ERG11	-2.69	30
TDH2	-2.69	30
UTP10	-2.69	30
PBI1	-2.68	7.42
RPP0	-2.68	30
FSH3	-2.67	11.53

RBD2	-2.67	17.21
MYO5	-2.67	17.91
THI6	-2.66	4.39
PHO91	-2.66	4.79
ATC1	-2.66	30
SMF1	-2.66	30
MPH2	-2.65	7.23
KDX1	-2.65	30
REX2	-2.65	30
MRPL32	-2.64	11.29
DED81	-2.64	30
DOT5	-2.64	30
EXP1	-2.64	30
YPS6	-2.63	9.13
RPS18B	-2.63	30
ALG3	-2.62	30
COS8	-2.61	30
DAL7	-2.60	16.78
COQ3	-2.60	17.31
DCG1	-2.59	3.94
MID2	-2.59	11.36
PUS4	-2.59	13.06
CUR1	-2.59	30
RGA1	-2.59	30
EMI2	-2.58	30
GRS1	-2.58	30
HTS1	-2.57	30
NCP1	-2.55	30
PSA1	-2.55	30
SHE3	-2.54	5.03
DOT1	-2.54	7.43
TFG1	-2.54	8.41
AXL1	-2.54	11.29
ERG5	-2.54	30
MCD4	-2.54	30
TOS6	-2.53	2.36
GCV3	-2.53	30
GUS1	-2.53	30

SEC11	-2.53	30
SMI1	-2.53	30
MRX1	-2.52	12.67
APE1	-2.52	30
POP1	-2.52	30
QCR2	-2.52	30
PER1	-2.51	16.50
COS7	-2.50	5.72
THO1	-2.50	6.62
DDI2	-2.50	7.71
POM33	-2.50	30
RNH203	-2.50	30
YSP3	-2.50	30
AQY3	-2.49	30
ATP2	-2.49	30
BNA1	-2.49	30
RPL11A	-2.49	30
RPP2A	-2.49	30
PML39	-2.48	10.85
HGH1	-2.48	13.13
SNZ3	-2.48	13.43
HAM1	-2.48	14.86
FRM2	-2.48	16.31
ARR3	-2.47	5.38
FMP33	-2.47	30
RPL15A	-2.46	30
SEC61	-2.46	30
MRS1	-2.45	11.94
RUD3	-2.45	30
SRO9	-2.44	8.25
LSC2	-2.43	30
GEA2	-2.42	30
GND1	-2.42	30
RPS20	-2.42	30
BBP1	-2.41	6.97
EFR3	-2.41	14.91
CBT1	-2.41	30
HXT7	-2.41	30

KTR2	-2.41	30
LDB17	-2.40	11.26
BUD7	-2.40	30
THR4	-2.40	30
YVH1	-2.39	7.51
IQG1	-2.39	15.82
CRH1	-2.39	30
MPC2	-2.39	30
GIN4	-2.38	9.47
TAF11	-2.38	13.31
SHS1	-2.38	17.72
DAD4	-2.38	30
RRT12	-2.37	6.18
OSW1	-2.37	13.73
APM4	-2.37	30
RPL2B	-2.37	30
TSA2	-2.37	30
ACO2	-2.36	30
RFC5	-2.35	6.62
NPY1	-2.35	9.97
ERG1	-2.35	30
EPT1	-2.34	6.52
ATF2	-2.34	6.94
URA1	-2.34	18.52
PAL1	-2.34	30
CLP1	-2.33	6.24
PHS1	-2.33	9.72
MYO3	-2.33	11.71
SNZ2	-2.32	9.00
CUP9	-2.32	15.59
DOP1	-2.32	17.96
NUP145	-2.32	30
SNO3	-2.31	9.72
BUD14	-2.31	30
PHO13	-2.31	30
BSC5	-2.30	11.64
DUG1	-2.30	30
SPS22	-2.29	3.42

KIP2	-2.29	5.96
RBS1	-2.29	10.05
SRB2	-2.29	14.15
GPM3	-2.29	16.01
ACA1	-2.29	30
ACT1	-2.29	30
GAS5	-2.29	30
MDL1	-2.29	30
NSA1	-2.29	30
SER3	-2.29	30
RPC25	-2.28	9.64
CAR1	-2.27	30
RPL22A	-2.27	30
RPS28B	-2.27	30
URA6	-2.27	30
BDS1	-2.26	11.27
ARP2	-2.26	30
EMC1	-2.26	30
HSP10	-2.25	30
KCH1	-2.25	30
HXT16	-2.24	5.54
MIN4	-2.24	10.47
AAP1	-2.24	30
GAL80	-2.24	30
HIP1	-2.24	30
KAR2	-2.24	30
HXT8	-2.23	13.93
PRO3	-2.23	30
SEC31	-2.23	30
SHE1	-2.22	3.02
DIT2	-2.22	5.78
TIR4	-2.22	6.63
CDC5	-2.22	11.71
CRS1	-2.22	17.63
ACS2	-2.22	30
YPF1	-2.22	30
YRR1	-2.22	30
HOF1	-2.21	3.15

TAN1	-2.21	7.35
BIK1	-2.21	11.15
ERR1	-2.21	13.33
STD1	-2.21	15.13
ADD37	-2.21	30
GFA1	-2.21	30
MDL2	-2.21	30
SEC28	-2.21	30
URA3	-2.21	30
EDS1	-2.20	4.15
ARC19	-2.20	30
FTR1	-2.20	30
HAT2	-2.20	30
NUG1	-2.20	30
TOS2	-2.20	30
SFH5	-2.19	5.46
SET4	-2.19	7.16
ERG8	-2.19	12.93
SSK22	-2.19	13.20
RFC1	-2.18	9.90
ADE6	-2.18	30
ERG13	-2.18	30
PMT2	-2.18	30
RPL23B	-2.18	30
TYW1	-2.18	30
FAL1	-2.17	6.88
SCW10	-2.17	17.02
YSF3	-2.17	30
RCE1	-2.16	2.69
CDA1	-2.16	5.03
SOR1	-2.16	17.70
RSR1	-2.16	18.39
ASN1	-2.16	30
ODC2	-2.16	30
DSF2	-2.15	2.47
COP1	-2.15	30
HDA1	-2.15	30
SNZ1	-2.15	30

SPT4	-2.15	30
URB2	-2.14	8.63
RPS11B	-2.14	10.50
ADH6	-2.14	30
GTT2	-2.14	30
MYO2	-2.14	30
RPL42B	-2.14	30
KTR5	-2.13	14.40
GSP1	-2.13	30
TCD2	-2.13	30
GUA1	-2.12	30
LYS12	-2.12	30
CGI121	-2.11	5.73
FRD1	-2.11	12.45
SSO2	-2.11	14.22
ADE3	-2.11	30
PAU4	-2.10	3.73
POF1	-2.10	6.54
RKM2	-2.10	7.26
HXT1	-2.10	30
ECL1	-2.09	3.99
RCK1	-2.09	7.48
ADE1	-2.09	12.55
MGL2	-2.09	13.89
CYC1	-2.09	30
MAS2	-2.09	30
MRPL7	-2.08	30
SIP3	-2.07	3.66
GEP3	-2.07	7.70
CDC14	-2.07	8.97
MRX4	-2.07	13.22
DOA1	-2.07	30
RIM21	-2.07	30
RPL11B	-2.07	30
RPS8B	-2.07	30
MAK10	-2.06	7.57
SED4	-2.06	9.03
ATP5	-2.06	30

PFY1	-2.06	30
RPL2A	-2.06	30
AAD4	-2.05	7.33
FRE7	-2.05	9.05
SMP1	-2.05	12.42
TPC1	-2.05	30
PUL3	-2.04	5.72
SPE4	-2.04	10.32
YMC1	-2.03	5.40
SDD1	-2.03	7.16
TCB2	-2.03	14.16
RPS12	-2.03	30
TDA8	-2.02	7.26
ERR2	-2.02	11.84
HER2	-2.02	13.15
COX5B	-2.02	30
CPR6	-2.02	30
RPL20B	-2.02	30
URA7	-2.02	30
SUT1	-2.01	2.09
ARP1	-2.01	5.03
SNO2	-2.01	7.71
ARP5	-2.01	9.05
TVP18	-2.01	17.35
ARL1	-2.00	4.90
ALG1	-2.00	6.22
HXT15	-2.00	7.03
TEA1	-2.00	7.32
MRP51	-2.00	30
PHO3	-1.99	3.17
NDT80	-1.99	3.96
HCS1	-1.99	5.04
SOR2	-1.99	17.26
RPS21B	-1.99	30
IFA38	-1.98	8.12
MPD1	-1.98	12.33
CTH1	-1.98	30
TDH3	-1.98	30

BCH1	-1.97	8.90
MMR1	-1.97	9.45
RPP1	-1.97	10.42
PRS4	-1.97	11.16
BNI4	-1.97	11.76
PPA2	-1.97	13.87
ECM33	-1.97	30
PMA1	-1.97	30
SNF8	-1.97	30
MSW1	-1.96	9.03
NUT1	-1.96	10.39
MET10	-1.95	6.62
MLC1	-1.95	30
REV7	-1.94	5.33
AIM14	-1.94	10.10
UTP11	-1.94	13.20
CWH43	-1.94	30
RAD59	-1.93	6.30
IPK1	-1.93	8.49
RPS5	-1.93	30
DRS2	-1.92	6.07
PRM10	-1.92	6.61
ELO1	-1.92	9.47
ALG6	-1.92	10.85
KTI12	-1.92	12.08
GCV2	-1.92	17.63
CHS7	-1.92	30
GAR1	-1.92	30
PMU1	-1.92	30
RRT5	-1.91	2.78
CSH1	-1.91	3.59
NUP84	-1.91	11.92
LAP2	-1.91	12.67
CHC1	-1.91	30
RPL34A	-1.91	30
SSB1	-1.91	30
TUB2	-1.91	30
YFH1	-1.91	30

GUP2	-1.90	2.82
SLD3	-1.90	7.41
ARH1	-1.90	9.42
RCF1	-1.90	18.06
LTP1	-1.90	19.04
MIC19	-1.89	12.58
EMA19	-1.89	16.52
RAM2	-1.89	18.13
RET3	-1.89	30
AYT1	-1.88	2.72
APL3	-1.88	4.92
OST4	-1.88	16.98
GSY2	-1.88	30
LSB1	-1.88	30
SAH1	-1.88	30
HTC1	-1.87	16.93
SSE1	-1.87	30
FRE1	-1.86	9.79
RIA1	-1.86	14.34
EGD1	-1.86	30
GDE1	-1.86	30
GUK1	-1.86	30
AVT5	-1.85	5.69
SEC10	-1.85	7.34
MCM5	-1.85	14.71
SMF2	-1.85	30
CPD1	-1.84	10.01
CHA1	-1.84	30
KAP114	-1.83	2.38
AIM44	-1.83	3.74
Unknown	-1.83	4.80
POL5	-1.83	15.88
RRT13	-1.82	7.03
SEC59	-1.82	8.43
SWE1	-1.82	10.84
NAT1	-1.82	15.61
SEC63	-1.82	30
CLB4	-1.81	4.52

GPA1	-1.81	5.53
ACF2	-1.81	7.28
TEL1	-1.81	15.82
TIF5	-1.81	19.89
RPS23A	-1.81	30
LMO1	-1.80	2.94
PLP2	-1.80	9.98
KRS1	-1.80	30
RPL9A	-1.80	30
MAD1	-1.79	7.62
YSR3	-1.79	8.40
KTR3	-1.79	9.45
SEC21	-1.79	30
GAL10	-1.78	2.12
MOB2	-1.78	3.21
DPP1	-1.78	11.71
ACE2	-1.77	2.07
IRC4	-1.77	3.48
PUS6	-1.77	3.97
ROD1	-1.77	9.10
PRM8	-1.77	9.13
CCW12	-1.77	30
PDI1	-1.77	30
RPL12A	-1.77	30
UGX2	-1.77	30
YBP1	-1.76	3.36
AIM24	-1.76	5.05
NMD4	-1.76	5.08
PEP3	-1.76	6.17
NAT5	-1.76	9.63
MTQ1	-1.76	9.68
SKI2	-1.76	10.13
RPC82	-1.76	16.21
ROM1	-1.76	18.65
ARG7	-1.76	30
ARO4	-1.76	30
HXT6	-1.76	30
RPL16B	-1.76	30

STV1	-1.76	30
DAL4	-1.75	3.86
CDC45	-1.75	4.54
DBP6	-1.75	5.02
NTE1	-1.75	7.44
PHD1	-1.75	16.71
SEC27	-1.75	30
ASH1	-1.74	3.20
TTI1	-1.74	4.45
AVT1	-1.74	13.92
SRP101	-1.74	14.10
RPS1B	-1.74	30
STU2	-1.73	8.35
INP53	-1.73	13.19
PTM1	-1.73	15.74
BNA6	-1.73	18.24
SEC62	-1.73	30
MED1	-1.72	5.69
CCA1	-1.72	9.46
ENT1	-1.72	10.27
PMT5	-1.72	10.34
FPR1	-1.72	30
RPL12B	-1.72	30
RPS0A	-1.72	30
MTO1	-1.71	10.29
MES1	-1.71	30
RPS31	-1.71	30
TPO3	-1.71	30
ZRT2	-1.71	30
PGU1	-1.70	2.74
TGA1	-1.70	9.17
STE23	-1.70	13.70
ADE17	-1.70	30
KRE6	-1.70	30
PCM1	-1.70	30
SEC23	-1.70	30
AIM10	-1.69	4.21
SPC29	-1.69	4.31

GEF1	-1.69	6.42
LTV1	-1.69	12.15
RRP3	-1.69	17.37
GGC1	-1.69	30
NCW2	-1.69	30
EMA17	-1.68	5.87
RKM4	-1.68	7.96
HSP78	-1.68	30
URC2	-1.67	4.80
ERP4	-1.67	10.68
ERG12	-1.67	11.23
PST1	-1.67	30
SVS1	-1.66	3.32
PHO12	-1.66	4.87
COG8	-1.66	5.61
MON2	-1.66	8.49
NIT3	-1.66	16.12
AGX1	-1.66	19.46
BIT61	-1.65	2.33
ILM1	-1.65	7.49
AIM7	-1.65	11.11
SGA1	-1.65	13.22
TOM5	-1.65	30
BOL3	-1.64	4.67
HIM1	-1.64	5.46
VAM7	-1.64	5.54
VPS38	-1.64	9.05
EMC5	-1.64	10.14
STT3	-1.64	30
AFB1	-1.63	2.69
POL1	-1.63	3.12
YCT1	-1.63	4.92
MMT1	-1.63	5.34
CTS2	-1.63	5.49
HIS6	-1.63	7.97
CKI1	-1.63	11.25
GIM3	-1.63	16.12
DPH2	-1.63	17.09

SNO1	-1.63	30
UBP2	-1.62	4.61
MCO14	-1.62	5.46
STE50	-1.62	6.56
RPL31B	-1.62	17.29
CYC7	-1.62	30
FUM1	-1.62	30
MCH5	-1.62	30
CBP2	-1.61	4.40
MVB12	-1.61	8.23
MUK1	-1.61	13.81
PUS1	-1.61	16.91
MRT4	-1.61	30
SPO75	-1.60	5.02
FMP30	-1.60	6.29
ABZ2	-1.60	7.13
SWI6	-1.60	7.32
RPL6A	-1.60	14.79
NOP58	-1.60	30
OLA1	-1.60	30
RPL35A	-1.60	30
TMA20	-1.60	30
KAP122	-1.59	5.45
ECM18	-1.59	6.86
NUP85	-1.59	8.90
SYM1	-1.59	11.94
SAM2	-1.59	17.36
RPS26B	-1.59	30
TOS4	-1.58	4.41
PRP42	-1.58	5.14
THI3	-1.58	5.81
BET5	-1.58	6.03
MAK11	-1.58	6.16
ERP2	-1.58	7.64
LOA1	-1.58	7.67
CAB5	-1.58	12.03
DPB2	-1.58	13.39
SDH4	-1.58	30

ICS2	-1.57	3.28
PPS1	-1.57	9.50
MCM2	-1.57	11.02
MET17	-1.57	12.52
DNF2	-1.57	14.32
HOL1	-1.57	30
RPL39	-1.57	30
RPL42A	-1.57	30
PHO11	-1.56	2.35
VPS45	-1.56	4.44
PUF4	-1.56	7.27
PKR1	-1.56	9.76
SAP185	-1.56	11.27
CDC12	-1.56	11.75
SFB3	-1.56	13.54
WSC2	-1.56	17.42
ADK1	-1.56	30
COX17	-1.56	30
RPS24B	-1.56	30
AZR1	-1.55	2.70
PIF1	-1.55	6.10
SHE2	-1.55	10.56
NOP2	-1.55	10.96
NMT1	-1.55	12.65
CCW14	-1.55	15.00
BUD9	-1.54	4.01
MMP1	-1.54	5.30
MAD2	-1.54	11.35
EMG1	-1.54	16.58
PFK27	-1.54	17.17
BGL2	-1.54	30
FCY1	-1.54	30
SPC24	-1.53	2.71
IRC8	-1.53	3.47
SIN4	-1.53	6.24
BUD3	-1.53	6.34
DEG1	-1.53	6.42
PIR1	-1.53	14.58

MIC10	-1.53	15.47
ALD5	-1.53	20.05
PGK1	-1.53	30
RPL8B	-1.53	30
VMA2	-1.53	30
IPL1	-1.52	3.31
MON1	-1.52	3.99
ELM1	-1.52	5.31
DBF2	-1.52	7.88
MRPL25	-1.52	10.93
AIM45	-1.52	15.27
VPS1	-1.51	3.69
PSD1	-1.51	6.98
RCM1	-1.51	11.82
PAU18	-1.50	2.37
FYV7	-1.50	11.92
TMA22	-1.50	13.20
RRB1	-1.50	16.17
FDO1	-1.49	2.88
SND2	-1.49	7.38
YEH2	-1.49	9.83
NCL1	-1.49	9.93
EBP2	-1.49	13.83
GAS1	-1.49	30
RPL13A	-1.49	30
MRX12	-1.48	4.80
PRR1	-1.48	5.19
MNT2	-1.48	7.77
PIN2	-1.48	10.08
PIC2	-1.48	30
RGT1	-1.48	30
RPL8A	-1.48	30
RPS24A	-1.48	30
TOA1	-1.48	30
ATM1	-1.47	3.91
SWI4	-1.47	4.12
PET117	-1.47	4.13
STE6	-1.47	4.97

PTC5	-1.47	7.14
NUP170	-1.47	8.97
VPS15	-1.47	13.52
FDC1	-1.46	4.64
PXL1	-1.46	5.53
RPS7B	-1.46	8.29
SPB1	-1.46	9.89
MRH1	-1.46	16.38
RPL26A	-1.46	18.69
RPL31A	-1.46	30
SED1	-1.46	30
XKS1	-1.46	30
UBC13	-1.45	2.55
DAL5	-1.45	4.11
END3	-1.45	13.57
SRL3	-1.45	15.67
LSC1	-1.45	16.81
HOM2	-1.45	30
RPS16A	-1.45	30
JLP2	-1.44	2.71
BAP3	-1.44	9.35
IRC22	-1.44	12.89
RPP1B	-1.44	30
RRP5	-1.44	30
PAU6	-1.43	3.77
GEP4	-1.43	6.95
OTU2	-1.43	7.39
SPP2	-1.43	10.11
CAF16	-1.43	11.71
ARP3	-1.43	16.34
ADO1	-1.43	30
ASC1	-1.43	30
MEX67	-1.43	30
RPS7A	-1.43	30
SAC6	-1.43	30
ORC1	-1.42	7.50
CYB2	-1.42	7.54
CCH1	-1.42	8.65

TPO1	-1.42	15.68
BOL2	-1.42	30
IRC10	-1.41	3.53
MRC1	-1.41	3.78
IRC6	-1.41	4.51
SEC3	-1.41	9.70
ANS1	-1.41	12.06
RPA49	-1.41	12.61
TAT2	-1.41	17.49
PST2	-1.41	30
AQY2	-1.40	3.70
ALG5	-1.40	4.16
MMM1	-1.40	5.40
TAF2	-1.40	5.97
MIN9	-1.40	6.44
NUP133	-1.40	8.23
RPL27B	-1.40	13.54
RPP2B	-1.40	30
SIR2	-1.39	2.83
ELO2	-1.39	3.21
DPH6	-1.39	5.27
MSY1	-1.39	6.24
RDH54	-1.39	7.88
MPH1	-1.39	7.97
LAT1	-1.39	17.14
IDI1	-1.39	30
PIG1	-1.38	2.75
RPS9B	-1.38	20.71
HRI1	-1.38	30
RPL27A	-1.38	30
RPS6A	-1.38	30
MNL2	-1.37	4.83
SNU13	-1.37	7.91
UTP22	-1.37	8.86
VID22	-1.36	5.01
ARG2	-1.36	5.28
CYC2	-1.36	6.93
HCH1	-1.36	30

SLT2	-1.36	30
PET112	-1.35	2.36
ALK2	-1.35	2.69
KAR3	-1.35	3.59
COG2	-1.35	4.85
VPS17	-1.35	4.90
SLD7	-1.35	6.55
UTP18	-1.35	11.13
ATP1	-1.35	30
CDC19	-1.35	30
RPS4A	-1.35	30
STU1	-1.34	3.82
ARF3	-1.34	4.24
LRO1	-1.34	7.62
RFS1	-1.34	12.62
HIS5	-1.34	20.15
SUP35	-1.34	30
AFT1	-1.33	2.65
RFM1	-1.33	2.78
CMR2	-1.33	2.79
FLO5	-1.33	3.13
GRC3	-1.33	4.68
RRP1	-1.33	5.21
CDC28	-1.33	7.27
DRS1	-1.33	11.25
ADE8	-1.33	20.16
CBF5	-1.33	30
RPL3	-1.33	30
RPS26A	-1.33	30
SAL1	-1.32	3.56
RAX1	-1.32	3.72
RSF2	-1.32	4.75
SET5	-1.32	7.05
TCB1	-1.32	10.23
GCR1	-1.32	13.52
AQR1	-1.32	14.17
GLR1	-1.32	16.65
RTC1	-1.31	3.23

RRM3	-1.31	3.81
PES4	-1.31	3.92
CBC2	-1.31	7.59
RPS19B	-1.31	30
SHM2	-1.31	30
AIM20	-1.30	2.25
RCY1	-1.30	2.68
VPS64	-1.30	3.10
RMP1	-1.30	3.81
SRV2	-1.30	5.03
SKI8	-1.30	5.14
FPR2	-1.30	6.34
SEC7	-1.30	7.95
RPS6B	-1.30	30
TMN3	-1.29	2.59
TUP1	-1.29	6.30
UTP14	-1.29	7.65
PUS7	-1.29	8.52
TUB3	-1.29	15.31
PSY3	-1.28	2.87
MCM21	-1.28	3.30
TAE1	-1.28	4.46
RAD53	-1.28	4.99
RPS8A	-1.28	30
ERG29	-1.27	2.61
HED1	-1.27	2.88
SEC1	-1.27	3.83
LNP1	-1.27	5.52
RHO2	-1.27	6.73
RPO26	-1.27	10.14
HSL1	-1.27	10.47
THS1	-1.27	10.79
COS1	-1.27	30
TEF4	-1.27	30
GIC1	-1.26	2.34
FUR4	-1.26	3.06
YUR1	-1.26	3.12
FYV5	-1.26	4.51

SCP1	-1.26	4.93
MSH2	-1.26	5.27
ATX1	-1.26	7.66
DPC29	-1.26	10.58
SME1	-1.26	10.74
BCH2	-1.25	2.42
COG3	-1.25	2.56
SEC16	-1.25	5.60
NOT5	-1.25	9.75
IDP1	-1.25	30
YEH1	-1.25	30
CYC8	-1.24	2.12
GIM5	-1.24	2.68
NMA1	-1.24	2.84
RSC3	-1.24	3.44
ORT1	-1.24	5.75
GGA2	-1.24	8.01
ALR2	-1.24	9.67
EAF7	-1.23	4.34
ADF1	-1.23	4.55
RPG1	-1.23	6.22
DPM1	-1.23	9.43
ECM16	-1.23	9.91
FET3	-1.23	11.00
TDA6	-1.22	2.12
RLP24	-1.22	12.09
RPL13B	-1.22	18.26
RPS13	-1.22	30
PRP28	-1.21	2.54
AST1	-1.21	3.75
YCH1	-1.21	4.58
SPR28	-1.21	5.27
NDJ1	-1.21	5.78
PPN2	-1.21	5.88
FCY21	-1.21	7.81
GLN4	-1.21	14.22
SUP45	-1.21	14.33
RPL21A	-1.21	16.11

TIF2	-1.21	30
CUE2	-1.20	3.29
TSC13	-1.20	3.57
AAD10	-1.20	8.13
YAT1	-1.20	13.32
ZWF1	-1.20	20.17
PDE1	-1.20	30
TCM62	-1.19	2.75
SDO1	-1.19	5.95
GPI16	-1.19	8.12
ATG41	-1.19	9.05
RFT1	-1.19	9.77
RPS29A	-1.19	19.53
RPL43B	-1.19	30
SSS1	-1.19	30
DDI3	-1.18	2.38
OGG1	-1.18	3.20
APL6	-1.18	6.46
PYC2	-1.18	6.61
VAR1	-1.18	6.85
FUR1	-1.18	9.14
SPC3	-1.17	2.05
CTF18	-1.17	2.39
UBP9	-1.17	4.41
REA1	-1.17	8.16
YSC84	-1.17	9.16
RPL1A	-1.17	19.05
PAU2	-1.16	2.13
CLA4	-1.16	2.98
COX10	-1.16	4.90
CTL1	-1.16	5.32
ERG28	-1.16	9.55
COM2	-1.16	11.52
ARO1	-1.16	19.27
PAC1	-1.15	2.22
YEL1	-1.15	2.66
CWP2	-1.15	3.86
VAC7	-1.15	4.45

COG4	-1.15	4.86
AYR1	-1.15	10.20
ARC1	-1.15	14.26
NOP56	-1.15	14.74
RPL7A	-1.15	14.89
RPS19A	-1.15	18.07
HXT17	-1.14	2.23
MDE1	-1.14	3.74
ERP3	-1.14	3.89
EMP70	-1.14	4.43
NGL2	-1.14	4.46
COS4	-1.14	4.59
SPC25	-1.14	4.84
NDC1	-1.14	5.74
ZRT3	-1.14	6.68
CDC11	-1.14	6.85
ATP19	-1.14	16.16
KRE9	-1.14	18.65
CNE1	-1.13	2.13
MSE1	-1.13	2.57
YPT7	-1.13	3.23
SRB6	-1.13	3.28
EPO1	-1.13	5.14
RRP45	-1.13	6.11
PRP19	-1.13	6.92
TDA10	-1.13	8.23
PAM18	-1.13	9.66
SOL4	-1.13	15.83
RPL30	-1.13	30
ISM1	-1.12	3.13
UIP4	-1.12	3.50
CEX1	-1.12	3.55
PTC4	-1.12	3.80
CHL1	-1.12	4.30
SHM1	-1.12	7.45
EMC4	-1.12	8.52
OCA4	-1.12	8.71
MTM1	-1.12	20.36

ERV25	-1.12	30
EMC2	-1.11	3.91
CTP1	-1.11	5.05
KIN4	-1.11	5.40
SEC65	-1.11	5.82
DIP2	-1.11	7.63
URA10	-1.11	8.67
ADE12	-1.11	14.97
PMT1	-1.11	17.99
RPL1B	-1.11	30
RPS28A	-1.11	30
MET18	-1.10	2.10
BIO3	-1.10	2.32
GWT1	-1.10	3.34
MRPL11	-1.10	5.34
SRY1	-1.10	11.52
PET9	-1.10	11.68
PCL5	-1.10	30
PEX6	-1.09	3.05
GDA1	-1.09	4.61
CTO1	-1.09	6.40
GCN20	-1.09	10.10
RPL16A	-1.09	11.29
APT1	-1.09	12.31
ERG25	-1.09	14.39
NNR2	-1.09	18.82
PRP5	-1.08	2.87
SMD3	-1.08	4.55
OMS1	-1.08	5.33
NEM1	-1.08	6.43
IPP1	-1.08	8.51
ERV29	-1.08	13.78
RPS11A	-1.08	15.86
GIS2	-1.08	30
RPL37A	-1.08	30
YEA6	-1.07	2.44
RRF1	-1.07	2.74
EMA35	-1.07	2.77

PTC2	-1.07	5.54
LCB5	-1.07	8.71
CYT2	-1.07	11.53
HSP60	-1.07	30
FAB1	-1.06	2.55
SGO1	-1.06	3.44
PUF6	-1.06	3.59
IXR1	-1.06	3.80
COQ6	-1.06	4.50
RIO2	-1.06	4.86
PTP2	-1.06	9.45
MRM1	-1.06	12.01
SSZ1	-1.06	18.43
CWP1	-1.05	2.36
SRP72	-1.05	3.02
SAE2	-1.05	3.70
NOP53	-1.05	5.39
CMP2	-1.05	5.61
SDH3	-1.05	9.61
NCA2	-1.04	2.05
SAD1	-1.04	2.22
HUG1	-1.04	2.32
PGM1	-1.04	2.39
YAP7	-1.04	2.42
EBS1	-1.04	4.82
RRN7	-1.03	2.20
TLG2	-1.03	2.29
DTD1	-1.03	2.30
UTP9	-1.03	3.01
FRS2	-1.03	5.74
PAM1	-1.03	6.47
ENO1	-1.03	30
NUP120	-1.02	2.48
FIN1	-1.02	3.06
PHO90	-1.02	3.37
DOG1	-1.02	5.77
RET1	-1.02	8.28
COS6	-1.02	30

RPL36B	-1.02	30
RPL41B	-1.02	30
GEA1	-1.01	2.43
TAF8	-1.01	2.76
PRP11	-1.01	3.14
SAM50	-1.01	3.48
STE14	-1.01	3.66
VPS8	-1.01	3.80
MRP4	-1.01	4.30
ANY1	-1.01	10.05
FSH1	-1.01	14.21
RPS2	-1.01	30
TEF2	-1.01	30
ZIM17	-1.01	30
PUS2	-1.00	2.22
DUN1	-1.00	4.19
BNA5	-1.00	4.27
RKI1	-1.00	4.67
RPS1A	-1.00	30

a) -Log10 (p-value) greater than 30 is indicated as 30.

Table S3. YPH499/Co40 gene with increased expression level due to mutation

Gene or locus tag	Log2 (fold change)	Log10 (p-value)[a)]
SPG1	11.98	30
SIP18	9.10	30
SPS100	9.10	30
GRE1	8.64	30
DPC7	8.28	30
RGI2	8.19	30
ADY2	7.11	30
CTA1	7.03	30
FMP16	6.36	30
POT1	6.34	30
ACS1	5.69	30
NDE2	5.62	30
HXT5	5.41	30
FOX2	5.35	30
SPG4	5.35	30
BOP2	5.20	30
PAU19	5.18	30
CSM4	5.17	30
ICY1	5.16	30
PUT1	5.10	30
PAI3	5.04	30
GDH3	5.03	30
TKL2	4.86	30
SRL4	4.81	30
HBT1	4.71	30
ALD3	4.63	30
DAN4	4.57	30
HEF3	4.47	30
MSC1	4.36	30
ATP8	4.35	8.91
IZH4	4.34	30
SHH4	4.33	30
MLS1	4.31	30
FMP48	4.29	30
UIP4	4.29	30
POX1	4.27	30

MEP1	4.21	30
PDR10	4.19	30
BDH2	4.16	30
DDR2	4.16	30
FAT3	4.10	30
FMP45	4.09	30
ATP6	4.06	30
ADH2	3.91	30
ARG82	3.88	30
GSC2	3.88	30
LSO1	3.86	30
PHM7	3.86	30
COX26	3.85	30
RTS3	3.82	30
RIM4	3.81	30
ECM4	3.78	30
JEN1	3.78	30
MEP2	3.72	30
SFA1	3.70	30
UTR5	3.65	30
INO1	3.62	30
PAL2	3.62	30
PUT4	3.62	30
CIS1	3.61	30
GTT1	3.61	30
PCK1	3.61	30
ATG32	3.59	30
GUT2	3.59	30
PLB2	3.57	30
PRR2	3.56	30
ALD4	3.51	30
MPC3	3.50	30
PXA1	3.39	30
YGP1	3.36	30
DBP1	3.34	30
FKS3	3.30	30
OM45	3.30	30
BLM10	3.29	30

SHC1	3.29	30
GPX1	3.27	30
DAN1	3.26	30
DCS2	3.26	30
MDH2	3.24	30
MND1	3.23	30
CLD1	3.21	30
SPG3	3.21	30
ATG1	3.20	30
CIT2	3.20	30
CMI8	3.18	30
FAA4	3.17	30
SOK2	3.16	30
ENA5	3.13	30
CAT8	3.11	30
ARE2	3.09	30
ATG34	3.07	30
CRC1	3.07	30
MHO1	3.07	30
YAT1	3.07	30
IRC15	3.06	30
PAU9	3.05	2.78
GIP2	3.05	30
STR3	3.04	30
ALD2	3.03	30
SPG5	3.03	30
ZTA1	3.02	30
DDI1	3.01	30
RAD4	3.01	30
OLE1	3.00	30
UME6	2.99	21.77
RSB1	2.99	30
YIG1	2.99	30
RTN2	2.95	30
UBC5	2.95	30
YPT53	2.95	30
CUP2	2.94	30
DIP5	2.94	30

VHS2	2.94	30
CMC4	2.93	30
SPO20	2.93	30
SDH2	2.92	30
ALT2	2.91	30
STB3	2.91	30
AAC1	2.90	30
PEX28	2.89	30
RIM101	2.87	30
GRX6	2.86	30
ATO3	2.85	30
FMP40	2.85	30
IDP3	2.85	30
ATG9	2.83	30
HFD1	2.82	30
SWI1	2.82	30
HVG1	2.81	17.19
PSP1	2.81	30
YAP1	2.80	30
SEF1	2.79	30
CPR1	2.77	30
PPQ1	2.77	30
SNT309	2.77	30
GAP1	2.76	30
FMP49	2.75	12.50
ENA2	2.75	30
KHA1	2.75	30
RAD55	2.75	30
SLZ1	2.75	30
FBP1	2.74	13.08
BYE1	2.73	30
DPA10	2.73	30
EAR1	2.72	30
COB	2.71	30
GPG1	2.71	30
ISF1	2.71	30
NGL3	2.70	30
YOR1	2.70	30

RRT1	2.69	30
MSN2	2.68	20.03
UGX2	2.68	30
AST2	2.67	23.70
BSC4	2.67	30
ENA1	2.67	30
SKS1	2.67	30
UBX5	2.67	30
YAT2	2.67	30
AMS1	2.66	30
ISU1	2.65	30
BSC2	2.63	30
CCC1	2.62	30
PEX11	2.62	30
SHH3	2.62	30
SPS19	2.62	30
SFC1	2.60	10.47
AGP1	2.60	30
CIT3	2.59	30
ECI1	2.59	30
GIS1	2.59	30
NUD1	2.59	30
RGS2	2.58	23.35
DIN7	2.56	17.50
CRF1	2.56	30
EDC2	2.54	30
MGR3	2.54	30
PHO4	2.54	30
SDH6	2.54	30
FZO1	2.53	17.52
APC11	2.53	30
MOH1	2.53	30
PFK26	2.53	30
ICL1	2.52	30
MEF2	2.52	30
SUM1	2.52	30
TRX2	2.52	30
URA10	2.52	30

IRC13	2.51	19.75
CTF19	2.51	30
JHD1	2.50	30
NFI1	2.49	30
MGA2	2.48	30
SUF4	2.47	2.38
DOC1	2.47	30
ILT1	2.47	30
NTH2	2.47	30
OTU1	2.47	30
RPT6	2.47	30
RAD51	2.46	30
HHF1	2.45	30
PIL1	2.45	30
MCO8	2.44	30
PTR2	2.43	30
FMP52	2.42	30
TPH3	2.42	30
CDC27	2.41	15.85
GLO1	2.41	30
NGR1	2.40	14.74
ATG36	2.40	18.35
ECM10	2.39	21.34
TFC3	2.39	22.57
ARO10	2.39	30
ATG11	2.39	30
RCN2	2.39	30
GTO3	2.38	7.53
AFG3	2.38	30
PET20	2.38	30
OPY1	2.37	19.08
GTO1	2.36	30
LGE1	2.36	30
MIX17	2.36	30
PRB1	2.36	30
GDH2	2.35	30
HXT4	2.34	30
IRC20	2.34	30

NPR1	2.34	30
ERV1	2.33	30
KIC1	2.33	30
VPS27	2.33	30
MAG1	2.32	30
YTA7	2.32	30
GIP1	2.30	15.11
YAK1	2.30	30
LPX1	2.29	30
MUB1	2.29	30
LCL1	2.28	21.28
HAA1	2.28	30
NBP35	2.28	30
SSH4	2.28	30
PMC1	2.27	30
SYG1	2.27	30
FAS1	2.26	30
KNS1	2.26	30
OM14	2.25	16.95
ARA2	2.25	18.10
COX1	2.25	30
MPO1	2.25	30
SPF1	2.25	30
YCP4	2.25	30
PEX9	2.24	11.06
TDA11	2.24	17.03
CCL1	2.24	30
FMS1	2.23	30
HYR1	2.23	30
SNQ2	2.23	30
SUR1	2.23	30
RNR3	2.22	7.11
PMP3	2.22	30
RIB1	2.22	30
SCS7	2.22	30
CPS1	2.21	30
SIP2	2.21	30
CWC23	2.20	2.86

FUB1	2.20	30
SND1	2.20	30
WAR1	2.19	18.06
CSR2	2.19	30
NDL1	2.19	30
SSM4	2.19	30
YME2	2.19	30
MIT1	2.18	5.57
TSC10	2.18	12.35
BI4	2.18	30
GPA2	2.18	30
SUE1	2.18	30
USV1	2.17	15.64
ATG39	2.17	30
BDF2	2.17	30
GYP7	2.17	30
HUA1	2.17	30
MSC3	2.17	30
RPN3	2.17	30
AVT3	2.16	30
GRX4	2.16	30
REH1	2.16	30
CBF1	2.14	11.07
PRP31	2.14	19.11
CDC31	2.14	30
MDH3	2.14	30
RAP1	2.14	30
SNC2	2.13	30
TPK1	2.13	30
HAL9	2.12	12.35
HMS1	2.11	20.05
PEX22	2.10	15.58
ATG4	2.10	30
GLC3	2.10	30
VHS3	2.10	30
BRE4	2.09	30
ICT1	2.09	30
UBC8	2.09	30

ACC1	2.08	30
APE3	2.08	30
CCS1	2.08	30
NAB3	2.08	30
PEX15	2.08	30
GPI10	2.07	16.33
CSC1	2.07	30
GET3	2.07	30
RAD54	2.07	30
SNA3	2.07	30
UGA2	2.07	30
ZWF1	2.07	30
PRM3	2.06	7.55
PEX18	2.06	10.99
ATH1	2.06	30
NAR1	2.06	30
PTK1	2.05	9.28
FHN1	2.05	10.31
ARA1	2.05	30
NNK1	2.05	30
PDR1	2.05	30
CAF40	2.04	11.93
SAF1	2.04	14.51
GNP1	2.04	19.86
MET30	2.04	30
RPT2	2.04	30
RPN8	2.03	30
UBX4	2.02	9.32
KGD2	2.02	30
MPE1	2.02	30
OLI1	2.02	30
MMO1	2.01	3.30
LEE1	2.01	3.31
FAA1	2.01	30
MPM1	2.01	30
YPT11	2.00	6.66
BPT1	2.00	30
GLT1	2.00	30

HUR1	2.00	30
MPD2	2.00	30
YSF3	1.99	10.57
WHI5	1.99	18.17
ATO2	1.99	30
COX16	1.99	30
GPN2	1.99	30
HUL5	1.99	30
TBS1	1.98	13.88
ATG8	1.98	30
DEF1	1.98	30
HHO1	1.98	30
SSL2	1.98	30
RKM5	1.97	7.60
GIS3	1.97	14.35
FAA2	1.97	15.96
CMR3	1.96	3.40
GPT2	1.96	19.24
CDC53	1.96	30
MPT5	1.95	9.69
RRG1	1.95	10.34
NUM1	1.95	30
AI5_ALPHA	1.94	30
GFD1	1.94	30
SNX4	1.94	30
UBC13	1.94	30
UGA4	1.93	3.53
MET8	1.93	3.76
VFA1	1.93	4.97
FMP23	1.93	5.47
GPI2	1.93	11.39
UMP1	1.93	30
TOD6	1.92	9.31
RSA1	1.92	12.20
BXI1	1.92	30
DAT1	1.92	30
GAD1	1.92	30
HRT1	1.92	30

PRE5	1.92	30
ULS1	1.92	30
YET2	1.92	30
CIP1	1.91	4.71
COX23	1.91	16.51
ROY1	1.91	17.33
PEP4	1.91	30
PEX30	1.91	30
TMC1	1.91	30
AHC1	1.90	7.67
LUG1	1.90	8.48
MRX8	1.90	8.84
ALP1	1.90	14.96
SLM3	1.90	30
TFG2	1.90	30
AI4	1.89	30
GSH1	1.89	30
PEX3	1.89	30
RRT8	1.89	30
SHE10	1.89	30
RCF3	1.88	5.67
ATG5	1.88	30
DDP1	1.88	30
RTG1	1.88	30
SSD1	1.88	30
WTM1	1.88	30
CDC123	1.87	8.49
HYM1	1.87	8.64
TGL4	1.87	30
TES1	1.86	3.86
GAL83	1.86	30
PMR1	1.86	30
RFA1	1.86	30
VID24	1.85	9.12
RTS2	1.85	11.31
AIM19	1.85	15.92
AIM9	1.85	30
DIA1	1.85	30

GLC8	1.85	30
GRR1	1.85	30
NQM1	1.85	30
PCP1	1.85	30
INP54	1.84	6.83
BUD23	1.84	30
PHB2	1.84	30
RRT6	1.83	9.84
TGL3	1.83	18.07
RNA14	1.83	30
SPT2	1.83	30
TRS31	1.82	30
HIT1	1.81	13.36
CAT2	1.81	14.59
PSH1	1.81	16.73
YPR1	1.81	30
SPC42	1.80	6.10
CDC23	1.80	9.21
NRD1	1.80	11.73
COX7	1.80	30
HSF1	1.80	30
GMC2	1.79	2.14
CTR3	1.79	4.43
PIG2	1.79	5.54
RRD1	1.79	18.12
PUT2	1.79	30
VID27	1.79	30
PRP18	1.78	8.69
KEL1	1.78	11.34
MVP1	1.78	13.16
PFU1	1.78	16.98
BCP1	1.78	17.45
LAP3	1.78	30
TPS2	1.78	30
DAS1	1.77	10.20
ICL2	1.77	12.31
VPS29	1.77	18.31
LSP1	1.77	30

SKN7	1.76	8.11
RRN3	1.76	15.75
PLB3	1.76	17.79
AI3	1.76	30
RAD14	1.75	9.96
CST6	1.75	13.19
MAF1	1.75	14.79
CAR2	1.75	15.77
PUT3	1.75	30
RPM1	1.75	30
AI2	1.74	30
ATG15	1.74	30
MRPL16	1.74	30
SKN1	1.74	30
TOP3	1.74	30
MOT2	1.73	8.39
WTM2	1.73	13.37
ACO1	1.73	30
TMA10	1.73	30
SNA4	1.72	5.52
SLX8	1.72	17.04
SNC1	1.71	4.81
CTK3	1.71	4.92
CAF120	1.71	6.83
ATG21	1.71	9.97
NTO1	1.70	6.47
HPA2	1.70	7.34
CDC34	1.70	30
GID8	1.70	30
GRE2	1.70	30
PRE10	1.70	30
RPT1	1.70	30
STE11	1.70	30
RAD30	1.69	7.04
TIM13	1.69	7.06
INO2	1.69	8.81
SPR6	1.69	11.99
APJ1	1.69	30

DBP2	1.69	30
IDP2	1.68	5.52
SWC5	1.68	7.44
PDR8	1.68	8.91
CAB2	1.68	8.93
ATG19	1.68	13.78
NAS6	1.68	16.42
ATG26	1.68	30
GLN1	1.68	30
GLO4	1.68	30
PRC1	1.68	30
SUR2	1.68	30
HOT1	1.67	3.21
IRA1	1.67	8.06
CCT3	1.67	30
DLD3	1.67	30
CHK1	1.66	5.60
MIN6	1.66	8.45
SGT1	1.66	12.23
AI5_BETA	1.66	30
PCT1	1.66	30
THI6	1.66	30
RTC6	1.65	30
MDV1	1.64	7.86
HXT9	1.64	8.27
CRP1	1.64	30
ISY1	1.64	30
MIN8	1.64	30
SCH9	1.64	30
PAU3	1.63	2.12
SCD5	1.63	8.51
HUB1	1.63	30
MNC1	1.62	6.34
SNF11	1.62	6.64
RAD16	1.62	12.77
DPB4	1.62	13.16
NBL1	1.62	14.32
CDC37	1.62	30

PGA2	1.62	30
SKP1	1.62	30
HEL1	1.61	5.84
YFT2	1.61	13.01
MLO1	1.61	13.32
CWC24	1.61	13.90
IMP2'	1.61	30
SHP1	1.61	30
SEN2	1.60	7.41
RSF1	1.60	9.01
MNN14	1.60	9.20
RSN1	1.60	11.08
BI2	1.60	30
CCT6	1.60	30
SAP155	1.60	30
UBI4	1.60	30
PGD1	1.59	5.55
YSA1	1.59	8.94
SAN1	1.59	11.20
UBX7	1.59	12.66
RPC31	1.59	12.68
HST1	1.59	30
YTP1	1.58	5.24
HSM3	1.58	5.27
COQ4	1.58	14.38
MKK2	1.58	16.49
ADY4	1.57	6.20
ARO9	1.57	10.90
SRC1	1.57	12.33
ESA1	1.57	30
PBI2	1.57	30
PDC6	1.56	3.29
OAF1	1.56	5.37
BEM1	1.56	6.43
VPS72	1.56	6.85
TUL1	1.56	8.07
SDP1	1.56	8.70
RPH1	1.56	12.31

RMD5	1.56	14.40
PDH1	1.56	16.63
MPC1	1.56	30
MXR1	1.55	11.28
MRPL28	1.55	11.33
GND2	1.55	30
SOL2	1.55	30
VNX1	1.54	3.68
YRR1	1.54	4.63
STE24	1.54	9.80
FAP1	1.54	12.79
CCT5	1.54	30
ISA1	1.54	30
IWR1	1.54	30
OAZ1	1.54	30
QCR6	1.54	30
RFA2	1.54	30
RNR2	1.54	30
YTA12	1.54	30
HOT13	1.53	2.23
unknown	1.53	4.23
MRX9	1.53	9.13
PTC1	1.53	12.44
TVP18	1.53	16.15
CCT8	1.53	30
HPF1	1.53	30
VAR1	1.52	3.57
MDM34	1.52	7.13
TRR2	1.52	9.76
NCB2	1.52	10.23
UBC6	1.52	13.98
DSC2	1.52	15.57
DNF1	1.52	16.41
FMP46	1.52	30
INH1	1.52	30
IRA2	1.52	30
RAD23	1.52	30
SPT15	1.52	30

CDC16	1.51	5.63
FZF1	1.51	10.32
NUP2	1.51	15.45
MNN5	1.51	30
RPN1	1.51	30
MCO76	1.50	4.06
DPC25	1.50	6.01
RAD3	1.50	7.37
CSS3	1.50	10.33
BRE1	1.50	12.28
MKK1	1.50	12.77
BI3	1.50	12.92
MAP1	1.50	13.88
UBP13	1.50	14.97
MCO10	1.50	30
PRE2	1.50	30
PRE3	1.50	30
RPT4	1.50	30
SPR1	1.49	3.44
ASG1	1.49	4.58
ELA1	1.49	5.00
PEX5	1.49	8.66
RVS161	1.49	30
SPT5	1.49	30
PTI1	1.48	3.18
EMP65	1.48	4.40
QDR1	1.48	4.58
NNF2	1.48	5.36
QNS1	1.48	13.29
AI1	1.48	30
PYC1	1.48	30
RPS27A	1.48	30
UBP6	1.48	30
FEX2	1.47	4.56
SNF1	1.47	5.03
RFA3	1.47	7.22
MET4	1.47	13.17
ORM2	1.47	30

SLG1	1.47	30
SEN34	1.46	2.16
SSU72	1.46	2.48
MNT4	1.46	2.75
ECM27	1.46	5.16
LDO16	1.46	5.38
PCI8	1.46	5.97
GOS1	1.46	6.82
VPS35	1.46	13.08
NYV1	1.46	30
RPT3	1.46	30
MCO32	1.45	2.59
MUM2	1.45	3.57
VAB2	1.45	4.47
PXA2	1.45	5.04
ECM2	1.45	6.99
ESL2	1.45	11.43
ARG80	1.45	13.13
KRE1	1.45	15.96
ADR1	1.45	30
NPC2	1.45	30
TYE7	1.45	30
UFD1	1.45	30
XYL2	1.44	2.01
ROG1	1.44	4.49
VPS55	1.44	10.27
ATG20	1.44	10.32
UGA1	1.44	10.55
INO4	1.44	11.52
ECM21	1.44	12.35
ZRT3	1.44	14.67
HSV2	1.44	30
PUP2	1.44	30
PBA1	1.43	5.33
BOI2	1.43	9.84
SNF3	1.43	12.55
TOS8	1.43	13.40
GAC1	1.43	30

LDB7	1.43	30
WHI3	1.42	2.67
RTG3	1.42	3.11
SPP41	1.42	3.18
COS9	1.42	4.14
TAD2	1.42	4.19
CPT1	1.42	6.78
CFD1	1.42	8.01
MIH1	1.42	9.35
IOC4	1.42	9.52
YNG2	1.42	11.69
AIM46	1.42	30
MDH1	1.42	30
MIA40	1.42	30
RPN13	1.42	30
STF1	1.42	30
TCP1	1.42	30
IBA57	1.41	2.72
FDH1	1.41	4.22
RRD2	1.41	7.53
NHP6B	1.41	15.17
RTK1	1.41	30
TAF12	1.41	30
BTT1	1.40	2.41
GAT2	1.40	3.31
RCI50	1.40	5.09
SHR5	1.40	5.13
FYV6	1.40	8.06
SMB1	1.40	9.49
PKH2	1.40	9.96
FET5	1.40	12.95
SPT6	1.40	14.71
RAM1	1.39	3.39
RPI1	1.39	3.50
MED2	1.39	5.36
VID30	1.39	7.39
RPO31	1.39	11.08
RNH70	1.39	15.71

COX15	1.39	30
GDI1	1.39	30
MSN5	1.39	30
YPF1	1.38	6.20
FHL1	1.38	6.86
ELP4	1.38	8.57
RSC2	1.38	10.36
SPT16	1.38	12.99
ERC1	1.38	13.87
SCS2	1.38	14.92
COX3	1.38	30
MRS6	1.38	30
STP4	1.37	3.65
HXT11	1.37	4.67
SNG1	1.37	5.96
TOM70	1.37	11.48
TFA2	1.37	12.03
OYE3	1.37	14.11
SDD3	1.37	15.22
RPS30B	1.37	30
BIG1	1.36	2.65
PET130	1.36	3.87
MPS3	1.36	4.12
CDH1	1.36	5.34
PAH1	1.36	5.50
PCL8	1.36	5.51
NFU1	1.36	5.79
NAM9	1.36	7.88
CAP1	1.36	8.95
RAD1	1.36	9.07
TRS23	1.36	11.36
PRE1	1.36	30
UBR1	1.36	30
HTA2	1.35	4.60
MCM1	1.35	5.27
ETR1	1.35	5.29
PIB1	1.35	8.48
FYV8	1.35	8.55

AFT2	1.35	9.01
UTR1	1.35	15.40
ARB1	1.35	30
BAP2	1.35	30
MIC26	1.35	30
OPI1	1.35	30
STE7	1.34	3.69
PEX31	1.34	4.48
MET28	1.34	6.31
TRM12	1.34	6.74
CAB4	1.34	7.97
FYV10	1.34	10.63
RCR1	1.34	11.32
MNR2	1.34	30
RDL1	1.34	30
NCA3	1.33	2.89
CDC8	1.33	4.56
BAG7	1.33	5.35
PEX19	1.33	10.94
EMP46	1.32	4.39
15S_RRNA	1.32	8.73
VPS62	1.32	11.13
BDF1	1.32	30
MTR2	1.32	30
HAP2	1.31	2.23
SEN54	1.31	3.06
SHE9	1.31	3.21
AQY1	1.31	3.70
AZF1	1.31	3.73
SDH8	1.31	5.57
HTL1	1.31	5.97
NIS1	1.31	6.95
PET100	1.31	10.07
ROX3	1.31	30
PDR3	1.30	2.27
SFL1	1.30	2.89
MED7	1.30	3.26
GPI19	1.30	3.32

SLO1	1.30	4.42
EAF3	1.30	7.14
SEG1	1.30	8.15
TIM21	1.30	15.10
DPL1	1.30	30
FIS1	1.30	30
SEA4	1.29	2.13
ISC10	1.29	2.32
GUD1	1.29	2.85
LEU3	1.29	3.26
JID1	1.29	4.05
MRK1	1.29	5.68
ORM1	1.29	7.36
FLC2	1.29	11.58
PSK1	1.29	12.54
SEC17	1.29	12.56
COA6	1.29	13.23
FMP10	1.29	14.34
KTR1	1.29	30
PRE9	1.29	30
NUP60	1.28	2.12
KRE5	1.28	2.68
SAW1	1.28	2.72
ASF2	1.28	3.02
PXP1	1.28	5.50
NHX1	1.28	5.98
PCL10	1.28	7.23
YFH1	1.28	7.31
RQC1	1.28	7.60
TOA1	1.28	9.46
QCR10	1.28	30
RPB4	1.28	30
TIP20	1.27	2.99
SDD4	1.27	3.51
VPS70	1.27	9.38
SCEI	1.27	13.66
IES6	1.27	14.49
CCT2	1.27	30

PFK1	1.27	30
RHO1	1.27	30
RPS27B	1.27	30
SOD1	1.27	30
SHU2	1.26	2.74
SUT2	1.26	3.05
SYS1	1.26	6.36
MFB1	1.26	7.83
MCH1	1.26	8.04
MDG1	1.26	8.21
MSP1	1.26	9.55
GIM4	1.26	9.77
BMH1	1.26	30
CUZ1	1.26	30
GLO2	1.26	30
PRE7	1.26	30
RGD1	1.25	2.73
MNE1	1.25	3.77
PHO2	1.25	4.44
UBP9	1.25	5.22
HRT3	1.25	6.57
CDC40	1.25	7.76
RSM10	1.25	9.98
CRM1	1.25	30
EFT1	1.25	30
PGA3	1.25	30
PRE4	1.25	30
REF2	1.24	3.22
DPI35	1.24	4.19
NPL4	1.24	6.03
MAM3	1.24	7.66
SSU1	1.24	12.26
ARF2	1.24	30
LPL1	1.24	30
NFS1	1.24	30
XRN1	1.24	30
MSG5	1.23	2.37
RAS1	1.23	2.55

DSL1	1.23	3.07
VLD1	1.23	3.08
PEX35	1.23	3.49
VID28	1.23	3.58
CSS2	1.23	4.47
GPB2	1.23	4.58
YPT32	1.23	5.93
PAN2	1.23	10.24
TRX3	1.23	14.10
RPB2	1.23	30
AIM11	1.22	2.02
RIT1	1.22	2.64
PIP2	1.22	2.84
COA4	1.22	2.94
NUR1	1.22	4.40
RRG9	1.22	5.31
INA17	1.22	5.77
LRE1	1.22	5.95
ITT1	1.22	7.41
ASK1	1.22	7.89
AIM41	1.22	9.45
NCR1	1.22	10.94
GUT1	1.22	30
RPN7	1.22	30
IBD2	1.21	3.53
ASR1	1.21	5.90
RSM22	1.21	6.90
TAF1	1.21	9.54
CTR2	1.21	10.35
RPN6	1.21	30
SEM1	1.21	30
RTR2	1.20	2.40
ETP1	1.20	3.18
TEL2	1.20	4.82
THO2	1.20	5.38
ALO1	1.20	5.69
YAF9	1.20	5.74
MRPL51	1.20	8.13

RCI37	1.20	8.95
MAG2	1.20	9.16
MRPS18	1.20	13.29
SRP54	1.20	13.71
DSK2	1.20	30
KGD1	1.20	30
YRA1	1.20	30
RAD50	1.19	3.08
OXP1	1.19	3.42
CKB2	1.19	7.87
RUP1	1.19	8.36
GTR1	1.19	9.37
GLC7	1.19	30
TYW3	1.18	2.64
UBS1	1.18	3.02
SNU114	1.18	3.08
RAV2	1.18	5.10
CDD1	1.18	6.28
RAD52	1.18	6.73
APP1	1.18	6.90
GPD1	1.18	11.26
FPK1	1.18	11.95
COA3	1.18	30
FAS2	1.18	30
VPS73	1.17	2.61
RMD1	1.17	3.09
SLY1	1.17	4.39
OSW7	1.17	4.64
TAF4	1.17	6.14
SSK2	1.17	6.46
ASK10	1.17	7.14
LYS20	1.17	9.53
CHZ1	1.17	14.21
PDS1	1.16	2.56
TRI1	1.16	2.72
PLC1	1.16	3.48
UBX3	1.16	3.63
PSF2	1.16	4.24

PAN6	1.16	4.54
RAD5	1.16	4.77
SLM4	1.16	4.98
DBP8	1.16	5.62
STH1	1.16	6.15
NST1	1.16	8.01
MDM32	1.16	8.68
YPS1	1.16	9.21
KTR4	1.16	15.70
TIF5	1.16	30
ARK1	1.15	2.44
PFA3	1.15	2.44
GPB1	1.15	3.68
PEX13	1.15	4.73
SWT21	1.15	4.85
SOD2	1.15	13.86
UBA1	1.15	30
OAR1	1.14	2.49
SPP1	1.14	4.04
HTZ1	1.14	4.43
PAP2	1.14	4.81
HEM2	1.14	4.97
PET191	1.14	5.39
VPS24	1.14	5.42
NEO1	1.14	6.29
FMP27	1.14	6.61
SLM1	1.14	6.86
MRP8	1.14	30
MOD5	1.13	2.03
CWC2	1.13	2.43
HRQ1	1.13	4.32
MTC6	1.13	5.10
RTR1	1.12	2.08
UBP14	1.12	2.26
SWF1	1.12	2.77
AIM18	1.12	2.88
ERR3	1.12	3.88
SDS3	1.12	4.28

GSM1	1.12	5.09
CEG1	1.12	5.42
SWR1	1.12	5.71
PCS60	1.12	5.96
SUA7	1.12	15.22
COX2	1.12	30
RRN5	1.11	2.38
TFB3	1.11	2.68
ATP25	1.11	3.38
HFL1	1.11	3.41
HEM1	1.11	3.43
RAD26	1.11	3.46
PCL7	1.11	5.04
GRX3	1.11	5.91
FRQ1	1.11	8.93
NPA3	1.11	9.30
TFS1	1.11	14.36
YKT6	1.11	30
DIA3	1.10	2.51
RNQ1	1.10	3.68
HIR1	1.10	3.70
RAD57	1.10	3.98
FAA3	1.10	4.32
MNN1	1.10	4.42
CTT1	1.10	5.25
MTC1	1.10	6.14
SAS5	1.10	6.68
PUP1	1.10	9.90
ASN2	1.10	30
HTA1	1.10	30
IZH1	1.09	2.32
KNH1	1.09	2.55
SLF1	1.09	3.03
AOS1	1.09	3.72
SIC1	1.09	5.15
GCD1	1.09	6.06
YHC1	1.09	6.18
SGV1	1.09	6.73

URH1	1.09	7.41
PDR16	1.09	10.32
FBP26	1.09	14.34
OPY2	1.09	30
PUP3	1.09	30
PKH1	1.08	2.49
TRM112	1.08	4.04
YIP3	1.08	4.78
SPO14	1.08	5.19
TRE2	1.08	6.62
RUB1	1.08	8.34
POG1	1.07	2.12
DPB11	1.07	2.40
MND2	1.07	2.42
RPD3	1.07	3.78
RPL18B	1.07	4.01
PDR15	1.07	4.38
GCR2	1.07	5.06
MOG1	1.07	5.69
RHO5	1.07	7.15
CSG2	1.07	9.26
SPO24	1.07	10.65
VPS21	1.07	15.10
MTG2	1.06	2.07
PRP16	1.06	2.97
ULA1	1.06	3.10
COQ21	1.06	3.73
YUR1	1.06	4.05
VBA1	1.06	4.76
YJU2	1.06	4.90
SOM1	1.06	5.57
PAR32	1.06	6.35
PDR12	1.06	8.66
FPS1	1.06	11.60
RPN5	1.06	30
SND3	1.06	30
MRX10	1.05	2.32
MRP2	1.05	2.81

ACK1	1.05	2.96
ASE1	1.05	2.98
STP1	1.05	3.18
NAM7	1.05	3.35
PRY2	1.05	3.40
GPM2	1.05	8.09
FTH1	1.05	8.23
INO80	1.05	13.36
DUG3	1.04	3.18
TMH18	1.04	3.22
NRT1	1.04	3.36
YIM1	1.04	4.17
RSC4	1.04	4.26
TAF14	1.04	4.51
RTT103	1.04	4.57
YAP3	1.04	4.75
LSM5	1.04	4.82
NCE101	1.04	5.45
MRPL10	1.04	6.08
BSD2	1.04	6.47
UCC1	1.04	9.31
COX13	1.04	30
UME1	1.03	2.94
BUD16	1.03	3.01
TMT1	1.03	3.64
COX11	1.03	4.08
TMS1	1.03	4.28
DID4	1.03	4.96
CTK1	1.03	5.12
APE4	1.03	5.21
MIN7	1.03	5.54
NNR1	1.03	9.24
NCE102	1.03	30
FOB1	1.02	2.10
GGA1	1.02	2.10
ASI3	1.02	2.14
TFB4	1.02	2.70
MRM2	1.02	3.29

RRN10	1.02	3.92
INM2	1.02	5.07
ARP8	1.02	5.75
MIM1	1.02	9.58
YLH47	1.01	2.09
INP52	1.01	2.19
SIP4	1.01	2.20
LAS1	1.01	2.21
TDA5	1.01	2.30
PFK2	1.01	30
TMH11	1.01	30
MGM1	1.00	3.26
MATALPHA2	1.00	3.40
SET1	1.00	3.53
NOT3	1.00	3.88
YAP5	1.00	5.29
CBP4	1.00	6.64
TOM1	1.00	7.01
GTS1	1.00	7.39
SPN1	1.00	8.14
PSG1	1.00	8.52
MET12	1.00	11.52
SEC53	1.00	30

a) -Log10 (p-value) greater than 30 is indicated as 30.

Table S4. YPH499/Co40 gene with decreased expression level due to mutation

Gene or locus tag	Log2 (fold change)	Log10 (p-value)[a)]
CUP1-1	-8.94	30
CUP1-2	-8.60	30
RGI1	-8.10	30
MRX15	-7.80	30
IRC7	-6.84	30
HSP30	-6.62	30
TEM1	-6.38	30
HXT3	-6.37	30
PDC1	-6.26	30
HXT14	-6.05	8.46
HSP150	-5.90	30
AAC3	-5.29	8.99
GAT4	-5.24	11.73
OPI10	-5.18	30
CDC10	-5.14	30
ARI1	-5.00	30
ULI1	-5.00	30
AIM17	-4.90	30
ALT1	-4.89	30
BFR1	-4.87	30
CIS3	-4.82	30
DCD1	-4.75	30
SUR7	-4.74	30
GAT3	-4.68	5.32
SUC2	-4.68	30
YPS6	-4.67	30
CDC3	-4.63	30
WSC3	-4.61	30
CLB2	-4.59	15.38
REE1	-4.55	30
DSF1	-4.53	30
FAR1	-4.45	30
UBP11	-4.44	30
CYK3	-4.43	30
TOH1	-4.31	30
CYS3	-4.30	30

TKL1	-4.29	30
CIR1	-4.20	30
SAP4	-4.16	15.77
MKC7	-4.14	30
MVD1	-4.10	30
SPS22	-4.09	7.65
SUN4	-4.09	30
EXG1	-4.06	30
NCW1	-4.06	30
COS2	-3.99	30
KEL3	-3.99	30
KIN3	-3.97	11.01
SSA4	-3.97	30
ABP1	-3.96	30
MAL32	-3.95	30
SPO74	-3.90	30
HXK2	-3.89	30
ERG4	-3.88	30
ANB1	-3.87	30
COQ2	-3.83	30
FIT2	-3.82	16.93
CYB5	-3.82	30
RAX2	-3.82	30
THI11	-3.81	4.66
HXK1	-3.81	30
PNP1	-3.81	30
SAM1	-3.81	30
RDS1	-3.75	14.81
COS3	-3.74	30
PAU18	-3.72	8.46
FPR4	-3.72	30
CLN2	-3.70	12.58
MSF1	-3.70	30
HOF1	-3.69	6.46
COS5	-3.69	30
SCW4	-3.69	30
ENO2	-3.67	30
AFR1	-3.65	30

BUD4	-3.63	17.37
MAN2	-3.63	30
MAL12	-3.62	30
YAP7	-3.60	30
PPZ1	-3.59	30
GSP1	-3.55	30
CRN1	-3.54	30
MLO50	-3.54	30
HOM3	-3.53	30
YIP5	-3.53	30
AMN1	-3.51	30
ATX2	-3.51	30
LYS9	-3.51	30
HBN1	-3.50	11.04
SRL1	-3.47	14.94
YVC1	-3.46	12.54
MME1	-3.46	30
HEM25	-3.44	30
YHM2	-3.43	30
EGO4	-3.42	30
CLN3	-3.41	2.83
RPC25	-3.41	17.05
BRP1	-3.41	30
BTN2	-3.40	30
CPR5	-3.40	30
GOR1	-3.39	30
RAI1	-3.39	30
RDT1	-3.36	15.68
FRE3	-3.35	12.03
BAR1	-3.34	8.27
HXT13	-3.34	30
SAM4	-3.34	30
SKM1	-3.34	30
YMD8	-3.34	30
DOG2	-3.33	30
PAN5	-3.31	30
YPK2	-3.31	30
NTG1	-3.30	12.28

RME1	-3.29	30
SRX1	-3.29	30
SHE1	-3.28	4.95
AHP1	-3.26	30
FKS1	-3.26	30
MYO4	-3.26	30
EXG2	-3.25	30
LDS1	-3.23	4.82
UTH1	-3.23	30
HAM1	-3.22	30
MEI4	-3.20	5.29
PAU13	-3.19	3.49
MPH2	-3.19	9.12
MRPL32	-3.19	14.59
FRM2	-3.19	30
KAE1	-3.19	30
MIS1	-3.19	30
POM33	-3.19	30
CLB1	-3.18	8.34
MIC60	-3.18	30
NUP188	-3.17	30
TRM9	-3.17	30
YRF1-3	-3.16	17.75
BUD7	-3.16	30
THR1	-3.15	30
XKS1	-3.14	30
RBH2	-3.13	16.31
ARO7	-3.12	30
DAL1	-3.11	30
GAS3	-3.11	30
TUB3	-3.11	30
VMA22	-3.10	9.42
TTI1	-3.10	10.02
RNH203	-3.10	30
TIP1	-3.10	30
TPM1	-3.10	30
IMA2	-3.09	30
SCP160	-3.09	30

STE4	-3.08	30
APC5	-3.07	9.45
ARO8	-3.06	30
SIM1	-3.06	30
TPS3	-3.06	30
YHB1	-3.06	30
FRS1	-3.05	30
SCW10	-3.05	30
UTR2	-3.05	30
BDH1	-3.04	30
DOT5	-3.03	30
IMD4	-3.03	30
ALK1	-3.02	6.51
DSE4	-3.02	15.37
PUS4	-3.02	16.09
CDA1	-3.01	8.20
YRF1-6	-3.01	15.55
YRF1-2	-3.01	15.56
BIO5	-3.01	30
PHR1	-3.01	30
SMC2	-3.01	30
BBP1	-3.00	9.44
CTR1	-3.00	30
EXP1	-3.00	30
MST1	-2.99	8.02
ERG26	-2.99	30
SRM1	-2.99	30
CRH1	-2.98	30
GRX8	-2.97	10.58
YRF1-7	-2.97	15.76
RSR1	-2.97	30
SWI5	-2.96	4.14
MID2	-2.96	13.74
GCV1	-2.96	30
REX2	-2.96	30
SIA1	-2.96	30
DCG1	-2.95	4.81
GFD2	-2.95	15.51

OYE2	-2.95	30
TAL1	-2.95	30
VTH1	-2.95	30
RRT5	-2.94	4.98
CSI2	-2.94	11.98
YRF1-1	-2.93	10.58
ERG6	-2.93	30
YPS5	-2.92	10.81
FET4	-2.92	30
PIR3	-2.92	30
QDR2	-2.91	16.06
VTH2	-2.91	30
PER1	-2.90	30
SRB2	-2.90	30
YRF1-8	-2.90	30
RRT12	-2.89	7.86
YRF1-5	-2.89	30
ADH7	-2.88	2.35
EGT2	-2.88	10.18
IMD2	-2.88	13.78
RRG7	-2.88	30
HIS2	-2.87	30
RBD2	-2.87	30
SMP1	-2.87	30
SDD1	-2.86	11.57
ARC40	-2.86	30
GGC1	-2.86	30
PRO1	-2.86	30
UPS3	-2.86	30
GND1	-2.85	30
PSD2	-2.84	30
TRE1	-2.84	30
SKT5	-2.83	30
DDI2	-2.82	8.94
TAN1	-2.82	10.43
PWP1	-2.82	30
CBT1	-2.81	30
KAR1	-2.80	9.01

OSW1	-2.80	17.13
PER33	-2.80	30
RGA1	-2.80	30
DED81	-2.79	30
LYS1	-2.79	30
ADA2	-2.78	30
SIL1	-2.78	30
CDC5	-2.77	15.98
GSH2	-2.77	30
MRS3	-2.77	30
SAC1	-2.77	30
SSA2	-2.76	30
SEC61	-2.75	30
SER2	-2.75	30
YRF1-4	-2.74	30
NNF1	-2.73	5.50
TRS120	-2.73	10.22
SNZ2	-2.73	11.19
MPH3	-2.73	12.48
EMP24	-2.73	30
NCP1	-2.73	30
YSP2	-2.73	30
RKM2	-2.72	10.37
AXL1	-2.72	12.25
BNA3	-2.72	15.26
PRM5	-2.72	30
DIA2	-2.71	10.69
ERD2	-2.71	30
SHE3	-2.70	5.21
THI80	-2.70	7.36
AIM26	-2.69	8.84
ERG27	-2.69	30
MPC2	-2.69	30
POP1	-2.69	30
PAU6	-2.68	9.25
SOP4	-2.68	30
PHS1	-2.67	11.72
YPP1	-2.67	13.53

CHS3	-2.67	30
STE5	-2.67	30
ACP1	-2.66	30
RPS17B	-2.66	30
PRM10	-2.64	10.35
RRI2	-2.64	12.20
COQ3	-2.64	17.54
SRD1	-2.64	30
LYS4	-2.63	30
SRO9	-2.62	9.06
MYO1	-2.62	11.97
SPH1	-2.62	13.84
TRK1	-2.62	30
BNA1	-2.60	30
URK1	-2.59	14.40
SSA1	-2.59	30
AVT5	-2.58	9.10
EHT1	-2.58	17.28
GRE3	-2.58	30
EPT1	-2.57	7.35
HDA1	-2.57	30
NSA1	-2.57	30
SMI1	-2.57	30
DSF2	-2.56	3.20
RCE1	-2.56	3.44
TWF1	-2.56	10.95
COS8	-2.56	30
RUD3	-2.56	30
MGL2	-2.55	18.45
GIN4	-2.54	10.59
CUP9	-2.54	17.80
HTS1	-2.54	30
IMA5	-2.53	7.58
PET8	-2.53	13.57
HIS6	-2.53	15.13
LSC2	-2.53	30
SES1	-2.53	30
TIR3	-2.52	8.04

RPN14	-2.52	11.74
HGH1	-2.52	13.33
YPD1	-2.52	30
SSK22	-2.51	15.88
SHS1	-2.51	30
MRS1	-2.50	12.23
BCH1	-2.50	12.78
SPC24	-2.49	5.12
KTR5	-2.48	17.89
ERG5	-2.48	30
TOS1	-2.48	30
CLN1	-2.47	5.72
GAS5	-2.47	30
CLB4	-2.46	6.91
MSW1	-2.46	12.41
ASN1	-2.45	30
MAS2	-2.44	30
RTC3	-2.44	30
TYS1	-2.44	30
TOS4	-2.43	2.20
LDB17	-2.43	11.41
ADH1	-2.43	30
PAL1	-2.43	30
KDX1	-2.42	18.18
RPL18A	-2.42	30
SPO77	-2.41	4.39
NUC1	-2.41	6.63
VMR1	-2.41	15.19
AGC1	-2.41	18.60
GRS1	-2.41	30
ALG3	-2.40	30
ARG7	-2.40	30
FTR1	-2.40	30
SEC11	-2.40	30
SPT14	-2.40	30
ARL1	-2.39	6.32
ARP5	-2.39	11.46
APM4	-2.39	30

ERG11	-2.39	30
SIP3	-2.38	4.41
ACT1	-2.38	30
TRR1	-2.38	30
KTI12	-2.37	16.52
STD1	-2.37	16.52
KTR2	-2.37	30
FAL1	-2.36	7.94
SOL3	-2.36	8.28
FRD1	-2.36	14.57
AQY3	-2.36	30
MRX1	-2.35	11.42
MYO5	-2.35	14.50
AXL2	-2.34	10.05
ENT1	-2.34	15.75
APE1	-2.34	30
GCV3	-2.34	30
HIP1	-2.34	30
RPS22A	-2.34	30
SAH1	-2.34	30
DAS2	-2.33	9.06
ODC2	-2.33	30
RAD59	-2.32	7.93
ANS1	-2.32	30
CRG1	-2.32	30
SKG6	-2.31	4.53
HXT8	-2.30	14.24
GPM3	-2.30	15.90
ECM33	-2.30	30
GCN1	-2.29	30
OST4	-2.29	30
CAR1	-2.28	30
CCW12	-2.28	30
HTC1	-2.28	30
SPT4	-2.28	30
POF1	-2.27	7.13
ELO3	-2.27	30
RPP2A	-2.27	30

EGH1	-2.26	4.96
PAU23	-2.26	7.52
SRP101	-2.26	30
PRM8	-2.25	12.71
MCD4	-2.25	18.69
RPP0	-2.25	30
SKG1	-2.25	30
TCD2	-2.25	30
PHO3	-2.24	3.74
MCD1	-2.24	5.90
LSB1	-2.24	30
SUT1	-2.23	2.54
ASH1	-2.23	4.49
CTF13	-2.23	6.05
SLD3	-2.23	9.29
ACF2	-2.23	9.86
ADH6	-2.23	30
CUR1	-2.23	30
HXT16	-2.22	5.62
RTC4	-2.22	12.29
TGL1	-2.22	15.93
EGD2	-2.22	30
RCM1	-2.22	30
JLP2	-2.21	4.73
BNI4	-2.21	13.96
ACO2	-2.20	30
EMI2	-2.20	30
ERG13	-2.20	30
RPL22A	-2.20	30
RPS18B	-2.20	30
TSA1	-2.20	30
THI12	-2.19	7.57
RPS11B	-2.19	10.68
PRS4	-2.19	12.88
IQG1	-2.19	14.03
ATP2	-2.19	30
GAR1	-2.19	30
HXT1	-2.19	30

MDL1	-2.19	30
OSH6	-2.18	10.15
SNZ3	-2.18	10.76
DOP1	-2.18	15.99
GDB1	-2.18	30
GFA1	-2.18	30
GUS1	-2.18	30
BIT61	-2.17	3.18
AAD3	-2.17	6.65
ERG20	-2.17	30
NUG1	-2.17	30
GEP3	-2.16	8.06
SWI6	-2.16	11.16
AAP1	-2.16	30
EMC1	-2.16	30
MRPL7	-2.16	30
PHD1	-2.16	30
PMU1	-2.15	30
RPS28B	-2.15	30
DAL7	-2.14	12.37
CRS1	-2.14	16.42
GEA2	-2.14	18.43
GPH1	-2.14	30
PHO13	-2.14	30
TOS3	-2.13	6.45
PSA1	-2.13	30
NDT80	-2.12	4.05
EMA19	-2.12	30
DIT2	-2.11	5.30
SNO2	-2.11	8.10
PML39	-2.11	8.60
CSE4	-2.11	15.14
BUD3	-2.10	9.98
MCM5	-2.10	17.89
QCR2	-2.10	30
LSM8	-2.09	3.18
TRP2	-2.09	13.96
DOT1	-2.08	5.61

PRO3	-2.08	30
TDH2	-2.08	30
NEJ1	-2.07	4.09
SVS1	-2.07	4.78
MMT1	-2.07	7.23
RBS1	-2.07	8.47
ARH1	-2.07	10.47
ACS2	-2.07	30
CTH1	-2.07	30
AIM44	-2.06	4.31
HED1	-2.06	5.62
MET10	-2.06	7.30
IFA38	-2.06	8.40
EFR3	-2.06	11.72
SSO2	-2.06	13.38
ATC1	-2.06	16.67
KRS1	-2.06	30
LYS12	-2.06	30
PMT2	-2.06	30
MCM16	-2.05	4.54
CLP1	-2.05	5.01
CCA1	-2.05	12.25
SNU13	-2.05	14.41
RPL15A	-2.05	30
RPS20	-2.05	30
ARP1	-2.04	4.91
RFC5	-2.04	5.59
PUL3	-2.04	5.78
NAT5	-2.04	12.15
ADD37	-2.04	30
ADE3	-2.04	30
PDI1	-2.04	30
TUB1	-2.04	30
SCO2	-2.03	9.98
DOA1	-2.03	30
HSP10	-2.03	30
KCH1	-2.03	30
BIK1	-2.01	9.48

ALD5	-2.01	30
RPL2B	-2.01	30
YSC84	-2.01	30
PMT5	-2.00	12.87
SEC31	-2.00	17.77
PMA1	-2.00	30
MOB2	-1.99	3.69
SOR2	-1.99	16.81
HAT2	-1.99	30
MLC1	-1.99	30
RPL23B	-1.99	30
SMF1	-1.99	30
PBI1	-1.98	4.76
GNA1	-1.98	7.72
MYO3	-1.98	9.07
BSC5	-1.98	9.10
ARC19	-1.98	30
GUA1	-1.98	30
PFK27	-1.98	30
SME1	-1.98	30
ARR3	-1.97	3.89
FDO1	-1.97	4.37
DUG1	-1.97	19.28
SMF2	-1.97	30
IRC8	-1.96	4.69
SNO3	-1.96	7.44
FRE7	-1.96	8.62
RPS7B	-1.96	12.59
BUD14	-1.96	18.34
RRT2	-1.95	2.67
SNF8	-1.95	30
URB1	-1.94	7.57
TPO2	-1.94	30
APL3	-1.93	5.12
PLP2	-1.93	11.08
NUP145	-1.93	15.79
LTP1	-1.93	19.47
RPL11B	-1.93	30

SPR3	-1.92	2.49
PHO91	-1.92	3.10
ACA1	-1.92	14.73
FMP33	-1.92	15.40
TEF4	-1.92	16.86
MYO2	-1.92	18.23
URA5	-1.92	30
SPC29	-1.91	5.14
RCK1	-1.91	6.60
IPK1	-1.91	8.38
MRX4	-1.91	11.48
EMC5	-1.91	12.84
TPA1	-1.91	17.98
MDL2	-1.91	18.26
EMG1	-1.91	30
GAL10	-1.90	2.16
SAE3	-1.90	3.41
PUS6	-1.90	4.33
AFT1	-1.90	4.52
TRM3	-1.90	5.79
FSH3	-1.90	6.81
KIP2	-1.89	4.25
COG8	-1.89	6.59
LOA1	-1.89	9.95
VPS63	-1.88	5.38
NPY1	-1.88	7.18
UTP10	-1.88	12.31
COP1	-1.88	30
ERG1	-1.88	30
EDS1	-1.87	3.15
DAL4	-1.87	4.18
AIM14	-1.87	9.33
ALG6	-1.87	10.41
ADE1	-1.87	10.55
GUK1	-1.87	30
MCH5	-1.87	30
RIM21	-1.87	30
SSB1	-1.87	30

GUP2	-1.86	2.92
TAF11	-1.86	9.25
TCB2	-1.86	12.21
CWH43	-1.86	30
HXT7	-1.86	30
SEC28	-1.86	30
ATF2	-1.85	4.81
GPA1	-1.85	5.66
DPH2	-1.85	30
RHO2	-1.84	11.71
SAP185	-1.84	14.08
ADE6	-1.84	30
RPL2A	-1.84	30
SER3	-1.84	30
TIF4632	-1.84	30
CSH1	-1.83	3.31
DUT1	-1.83	3.72
SWE1	-1.83	10.71
ECM31	-1.82	3.46
MAK10	-1.82	6.20
KAP122	-1.82	6.66
RFC1	-1.82	7.44
ERR2	-1.82	10.03
YOP1	-1.82	18.19
MRT4	-1.82	30
RPS12	-1.82	30
YHP1	-1.81	2.86
TFC8	-1.81	5.03
NMD4	-1.81	5.24
SOR1	-1.81	13.17
GAL80	-1.81	16.77
CYC8	-1.80	3.75
CLA4	-1.80	5.57
GEF1	-1.80	7.01
CPD1	-1.80	9.63
NUP84	-1.80	10.76
CAF16	-1.80	16.58
AIM45	-1.80	19.66

CPR6	-1.80	30
FLO5	-1.79	4.82
PRP42	-1.79	6.28
RSF2	-1.78	7.38
CDC28	-1.78	10.91
MUK1	-1.78	16.00
AFB1	-1.77	3.01
SEC59	-1.77	7.85
ROM1	-1.77	18.63
CHS7	-1.77	30
GTT2	-1.77	30
RPL42A	-1.77	30
ZRT1	-1.77	30
RMP1	-1.76	5.67
VAM6	-1.76	5.87
EMA17	-1.76	6.33
BDS1	-1.76	7.76
SET5	-1.76	10.88
LAP2	-1.76	10.92
MCM2	-1.76	13.21
COX5B	-1.76	30
CYC1	-1.76	30
URA2	-1.76	30
PES4	-1.75	6.00
ERG8	-1.75	8.96
CBC2	-1.75	11.50
HIM1	-1.74	5.73
ALG5	-1.74	5.76
NIT3	-1.74	17.06
BNR1	-1.73	3.30
HXT15	-1.73	5.62
ARG2	-1.73	7.63
KAR2	-1.73	30
PFY1	-1.73	30
SNO1	-1.73	30
SFH5	-1.72	3.95
VPS25	-1.72	6.21
RPP1	-1.72	8.54

OTU2	-1.72	9.72
PST1	-1.72	30
SEC27	-1.72	30
XDJ1	-1.72	30
KAR5	-1.71	2.20
PCL1	-1.71	2.88
DBP6	-1.71	4.94
RPL26A	-1.71	30
ART5	-1.70	4.76
BOL3	-1.70	4.83
CYC7	-1.70	30
FCY1	-1.70	30
RPL9A	-1.70	30
POL1	-1.69	3.29
SIN4	-1.69	7.20
SPE4	-1.69	7.80
STU2	-1.69	8.04
HER2	-1.69	9.90
BAP3	-1.69	11.67
GCV2	-1.69	14.14
URA6	-1.69	15.45
ARP2	-1.69	30
EGD1	-1.69	30
NCW2	-1.69	30
RPS23A	-1.69	30
ELO1	-1.68	7.73
KTR3	-1.68	8.50
MIC19	-1.68	10.54
MRPL25	-1.68	12.58
AVT1	-1.68	12.79
MRPL11	-1.67	9.91
RPL11A	-1.67	30
RPS8B	-1.67	30
SEC63	-1.67	30
THI5	-1.66	2.22
DRS2	-1.66	4.85
DBF2	-1.66	8.79
NAT1	-1.66	13.34

ALR2	-1.66	14.78
PGM1	-1.65	4.61
RSC3	-1.65	5.05
RRT13	-1.65	5.91
FET3	-1.65	17.18
KRE6	-1.65	30
TUB2	-1.65	30
FRE1	-1.64	7.81
ORT1	-1.64	8.50
RPC82	-1.64	14.20
RRB1	-1.64	18.56
CHC1	-1.64	30
STP22	-1.63	3.69
GDA1	-1.63	8.33
ERR1	-1.63	8.36
SKI2	-1.63	8.71
TRS65	-1.63	9.07
SYM1	-1.63	12.31
DAD4	-1.63	14.51
RRP3	-1.63	16.17
CAX4	-1.62	3.53
CDC45	-1.62	4.04
TAE1	-1.62	6.29
SED4	-1.62	6.45
SNZ1	-1.62	30
YCS4	-1.61	4.61
MET16	-1.61	5.15
CDC14	-1.61	6.09
NUT1	-1.61	7.55
EMC6	-1.61	13.13
MES1	-1.61	30
RPL39	-1.61	30
RPS21B	-1.61	30
LTE1	-1.60	2.06
CGI121	-1.60	3.81
SPO75	-1.60	5.01
MTO1	-1.60	9.20
YSC83	-1.59	14.56

ADO1	-1.59	30
HOM2	-1.59	30
RPL42B	-1.59	30
STT3	-1.59	30
CTS2	-1.58	5.06
YPT6	-1.58	5.10
VPS36	-1.58	8.52
ATP5	-1.58	30
RPL34A	-1.58	30
SEC21	-1.58	30
PUF4	-1.57	7.17
TVP15	-1.57	11.75
UTP15	-1.57	13.82
THR4	-1.57	14.85
ARO4	-1.57	30
PEP3	-1.56	5.04
ECM18	-1.56	6.62
MSH2	-1.56	7.30
POL5	-1.56	12.37
NOP58	-1.56	30
RET3	-1.56	30
SSE1	-1.56	30
STV1	-1.56	30
YET3	-1.56	30
THI22	-1.55	5.64
ERP2	-1.55	7.32
IRC22	-1.55	14.11
KRE9	-1.55	30
SEC62	-1.55	30
ZEO1	-1.55	30
COG3	-1.54	3.52
KAR3	-1.54	4.46
SPB1	-1.54	10.68
SGA1	-1.54	11.77
RPL12A	-1.54	30
RRM3	-1.53	4.74
UIP3	-1.53	5.31
PIF1	-1.53	5.93

NTE1	-1.53	6.07
MET17	-1.53	11.74
CCW14	-1.53	14.48
PGU1	-1.52	2.15
IRC4	-1.52	2.90
AIM10	-1.52	3.41
SPE2	-1.52	4.84
MCO14	-1.52	4.90
TRM11	-1.52	6.67
EMP70	-1.52	6.76
MON2	-1.52	7.49
RPL6A	-1.52	13.34
ERG28	-1.52	14.43
PUG1	-1.51	2.50
NOP19	-1.51	3.82
SAL1	-1.51	4.16
PIN2	-1.51	10.13
SAM2	-1.51	15.87
SRL3	-1.51	16.41
ARF3	-1.50	5.04
YGK1	-1.50	6.19
SFB3	-1.50	12.57
ATG41	-1.50	12.64
WRS1	-1.50	16.03
SEC23	-1.50	16.77
RPL20B	-1.50	30
RPS1B	-1.50	30
RPS5	-1.50	30
GIM5	-1.49	3.33
LOT6	-1.49	3.93
PRR1	-1.49	5.38
MIN4	-1.49	5.76
NGL2	-1.49	6.67
SLD7	-1.49	7.54
ERG12	-1.49	9.15
CKI1	-1.49	9.84
FPR1	-1.49	30
ECL1	-1.48	2.37

GEP4	-1.48	7.18
NOP2	-1.48	10.18
TEX1	-1.47	2.74
VPS41	-1.47	3.99
SEC1	-1.47	4.48
DPP1	-1.47	8.67
ERP4	-1.47	8.83
GIM3	-1.47	13.45
TOM40	-1.47	16.78
GYL1	-1.46	2.29
IRC10	-1.46	3.64
CBP2	-1.46	3.84
YCG1	-1.46	5.83
SCP1	-1.46	6.10
TAF2	-1.46	6.24
RDH54	-1.46	8.38
RAM2	-1.46	12.06
RPS0A	-1.46	30
MTL1	-1.45	2.14
ELO2	-1.45	3.34
ALG1	-1.45	3.82
SET4	-1.45	4.07
AST1	-1.45	4.69
NDJ1	-1.45	7.52
RIA1	-1.45	9.61
PUS1	-1.45	14.03
RMD6	-1.44	2.47
ATM1	-1.44	3.83
MFA2	-1.44	4.77
STE6	-1.44	4.83
RRP1	-1.44	5.62
COG4	-1.44	6.66
EPO1	-1.44	7.28
PKR1	-1.44	8.57
CAB5	-1.44	10.24
RPO26	-1.44	12.00
GAS1	-1.44	30
PAC11	-1.43	2.85

NMA1	-1.43	3.40
URB2	-1.43	3.77
MRC1	-1.43	3.81
DPH6	-1.43	5.48
PPN2	-1.43	7.30
NCL1	-1.43	9.24
INP53	-1.43	9.68
CDC12	-1.43	10.02
BNA6	-1.43	13.44
PAM18	-1.43	13.85
ADK1	-1.43	30
PCM1	-1.43	30
ALK2	-1.42	2.83
TLG1	-1.42	3.54
PET117	-1.42	4.00
MMP1	-1.42	4.65
SEC16	-1.42	6.63
SPR28	-1.42	6.63
YEH1	-1.42	9.09
MRP51	-1.42	13.95
CHA1	-1.42	30
AZR1	-1.41	2.14
POP5	-1.41	3.62
CEX1	-1.41	4.77
MAD1	-1.41	5.24
TRM82	-1.41	7.33
VAN1	-1.41	8.81
RPL31B	-1.41	13.77
RPL35A	-1.41	30
SED1	-1.41	30
SWI4	-1.40	3.72
MSY1	-1.40	6.05
RPL16B	-1.40	30
RPS26B	-1.40	30
MTQ1	-1.39	6.84
PPS1	-1.39	7.86
STE23	-1.39	9.97
RPL1B	-1.39	30

THS1	-1.39	30
KIN4	-1.38	7.34
LTV1	-1.38	8.76
SEC3	-1.38	9.13
NMT1	-1.38	10.55
TMA20	-1.38	11.28
MIC10	-1.38	13.08
LSC1	-1.38	15.27
SRY1	-1.38	16.46
RGT1	-1.38	30
YUH1	-1.37	3.30
LNP1	-1.37	6.31
TRA1	-1.37	9.78
URA1	-1.37	11.96
PIC2	-1.37	30
MSM1	-1.36	2.29
HUG1	-1.36	3.43
MIN9	-1.36	6.13
GDE1	-1.36	12.65
HXT17	-1.35	2.75
SKI8	-1.35	5.34
UBP8	-1.35	5.39
PSD1	-1.35	5.77
DNF2	-1.35	11.19
ADE17	-1.35	14.91
HSP78	-1.35	30
RPS31	-1.35	30
MSD1	-1.34	2.20
BCH2	-1.34	2.79
NRK1	-1.34	4.05
ISM1	-1.34	4.13
CTP1	-1.34	6.64
DPC29	-1.34	11.31
RFS1	-1.34	12.55
RLP24	-1.34	13.94
MEX67	-1.34	30
PUN1	-1.34	30
RPL12B	-1.34	30

MST27	-1.33	2.64
MAK11	-1.33	4.63
NDC1	-1.33	7.18
PTM1	-1.33	10.41
GSY2	-1.33	18.53
MTM1	-1.33	30
RPS19A	-1.33	30
DTD1	-1.32	3.14
ATP23	-1.32	5.33
TMA19	-1.32	30
UBC12	-1.31	2.21
RAX1	-1.31	3.65
YMC1	-1.31	5.22
CDC11	-1.31	8.40
RPS9B	-1.31	18.74
ARO1	-1.31	30
SEC10	-1.30	4.13
RCY1	-1.29	2.68
DUG2	-1.29	2.70
FDC1	-1.29	3.92
TRM10	-1.29	4.82
PUS7	-1.29	8.50
ISU2	-1.29	11.02
LCB5	-1.29	11.38
HRI1	-1.29	30
MRX12	-1.28	3.79
RAD53	-1.28	4.86
NUP85	-1.28	6.17
TUM1	-1.28	6.18
RRP45	-1.28	7.16
MAD2	-1.28	8.29
FYV7	-1.28	9.18
EBP2	-1.28	10.81
HOL1	-1.28	16.36
POR1	-1.28	30
UGP1	-1.28	30
GIC1	-1.27	2.38
ICS2	-1.27	2.41

UBP16	-1.27	3.18
END3	-1.27	10.96
ARP3	-1.27	13.38
IDP1	-1.27	30
RPL8B	-1.27	30
RPP1B	-1.27	30
YVH1	-1.26	3.32
PRP5	-1.26	3.36
AAD4	-1.26	3.60
SRB7	-1.26	4.53
FPR2	-1.26	5.91
UTP21	-1.26	7.68
RPL14A	-1.26	15.43
PDE1	-1.26	30
ZUO1	-1.26	30
STE50	-1.25	4.39
DOG1	-1.25	7.85
GGA2	-1.25	8.03
FSH1	-1.25	19.87
BOL2	-1.25	30
COS1	-1.25	30
CIK1	-1.24	3.62
MMR1	-1.24	4.67
RKM4	-1.24	4.85
MNT2	-1.24	5.91
BGL2	-1.24	30
YEF3	-1.24	30
CIN2	-1.23	2.59
MON1	-1.23	2.78
TIP41	-1.23	2.91
BUD9	-1.23	2.97
AIM24	-1.23	2.99
VHT1	-1.23	4.15
ROD1	-1.23	5.15
ORC1	-1.23	6.05
TIM8	-1.23	6.86
COX17	-1.23	30
RPL43B	-1.23	30

ELM1	-1.22	3.79
COG2	-1.22	3.98
SDO1	-1.22	6.18
IST3	-1.22	7.03
DPM1	-1.22	9.04
ECM16	-1.22	9.68
RPP2B	-1.22	30
PRP2	-1.21	3.00
VAC17	-1.21	4.81
SUP45	-1.21	14.08
CDC19	-1.21	30
SAC6	-1.21	30
TRL1	-1.20	2.77
DEG1	-1.20	4.36
ZDS1	-1.20	4.78
SHM1	-1.20	8.11
TPM2	-1.20	8.64
NOT5	-1.20	8.95
DRS1	-1.20	9.45
RPL31A	-1.20	18.66
ATX1	-1.19	6.77
MRH1	-1.19	11.59
TRP5	-1.19	30
TEA1	-1.18	2.42
SRB8	-1.18	2.66
TAF8	-1.18	3.56
RMD9	-1.18	4.08
YSP3	-1.18	4.47
MPH1	-1.18	6.11
RPL6B	-1.18	6.11
NUP170	-1.18	6.27
HEM12	-1.18	6.93
AQR1	-1.18	11.79
RPS19B	-1.18	30
TDH3	-1.18	30
IPL1	-1.17	2.16
INP51	-1.17	2.58
BET5	-1.17	3.90

UTP13	-1.17	6.36
RPS24B	-1.17	18.35
YRO2	-1.17	20.29
RPS13	-1.17	30
TNA1	-1.17	30
CTF18	-1.16	2.37
TRM2	-1.16	4.73
SHE2	-1.16	6.70
RPL27B	-1.16	9.87
PTP2	-1.16	10.62
YIH1	-1.16	11.43
RPL27A	-1.16	30
RPS24A	-1.16	30
TIF11	-1.16	30
ARD1	-1.15	2.27
MCM21	-1.15	2.81
OST3	-1.15	3.23
COX10	-1.15	4.85
RPG1	-1.15	5.44
AGX1	-1.15	10.63
VMA16	-1.15	21.12
HXT6	-1.15	30
SLT2	-1.15	30
CWP1	-1.14	2.47
RRN7	-1.14	2.51
RTC1	-1.14	2.71
TVP38	-1.14	4.91
CTL1	-1.14	5.16
LRO1	-1.14	5.85
FRS2	-1.14	6.52
EMW1	-1.14	7.17
RCF1	-1.14	8.08
RRP5	-1.14	18.35
PMT1	-1.14	18.47
RPL8A	-1.14	30
MSB2	-1.13	2.74
TSC11	-1.13	3.34
CDC9	-1.13	3.85

RIO2	-1.13	5.15
PPA2	-1.13	5.79
CCH1	-1.13	5.97
AIM7	-1.13	6.19
PAM1	-1.13	7.14
TPN1	-1.13	10.81
COM2	-1.13	10.88
FUM1	-1.13	12.40
SWD2	-1.12	2.63
DAL5	-1.12	2.78
MED1	-1.12	2.90
UTP8	-1.12	3.42
ADF1	-1.12	3.92
OMS1	-1.12	5.51
APL6	-1.12	5.68
NSA2	-1.12	7.22
IPP1	-1.12	8.81
ADE8	-1.12	14.94
TOF1	-1.11	2.62
STU1	-1.11	2.90
PEX6	-1.11	3.17
GRC3	-1.11	3.44
FYV5	-1.11	3.63
PCL9	-1.11	4.73
GPI16	-1.11	7.07
MRM1	-1.11	12.68
PCL5	-1.11	30
SHM2	-1.11	30
RTA1	-1.10	2.20
MTF2	-1.10	3.52
CYB2	-1.10	5.03
NAF1	-1.10	5.13
GRX7	-1.10	5.33
RKI1	-1.10	5.39
SEC7	-1.10	6.11
FCF2	-1.10	11.69
RPS7A	-1.10	30
PUS2	-1.09	2.49

PHO12	-1.09	2.59
ERP3	-1.09	3.61
VPS75	-1.09	3.97
MPD1	-1.09	4.93
PTC2	-1.09	5.62
PRP19	-1.09	6.49
MCM4	-1.09	7.82
PIR1	-1.09	8.40
RPL13A	-1.09	13.04
RPL7A	-1.09	13.35
BZZ1	-1.08	3.46
GET1	-1.08	4.73
SEC65	-1.08	5.51
CMP2	-1.08	5.72
MID1	-1.08	6.46
HCS1	-1.07	2.01
YCH1	-1.07	2.02
SNL1	-1.07	3.38
FMP30	-1.07	3.44
GCD10	-1.07	3.60
DUN1	-1.07	4.49
RPB5	-1.07	5.11
RPA49	-1.07	8.03
PET9	-1.07	11.09
ECO1	-1.06	2.39
LDS2	-1.06	2.92
SAE2	-1.06	3.73
SND2	-1.06	4.40
CYC2	-1.06	4.63
NNT1	-1.06	5.49
NUP57	-1.06	6.90
RPL22B	-1.06	8.50
ATP1	-1.06	17.96
ARG4	-1.06	30
RPL3	-1.06	30
VPS16	-1.05	3.27
ILM1	-1.05	3.71
ABZ2	-1.05	3.77

CHL1	-1.05	3.91
SMD3	-1.05	4.21
SPC25	-1.05	4.34
NOP53	-1.05	5.22
MRPS8	-1.05	8.17
GLR1	-1.05	11.24
TAF9	-1.05	12.41
SUP35	-1.05	20.16
RPS4A	-1.05	30
EXO70	-1.04	2.19
MCO12	-1.04	2.59
EMC2	-1.04	3.56
TAT2	-1.04	10.69
HIS5	-1.04	13.12
RPL1A	-1.04	15.56
ERV25	-1.04	30
RTT107	-1.03	2.62
PXL1	-1.03	3.28
RNT1	-1.03	4.92
NOC3	-1.03	5.05
ZRT2	-1.03	5.67
RPS16A	-1.03	14.22
SAD1	-1.02	2.15
SST2	-1.02	2.19
NUP120	-1.02	2.44
MVB12	-1.02	4.05
FAF1	-1.02	4.37
UPF3	-1.02	4.37
MRP1	-1.02	6.33
HSL1	-1.02	7.36
ASC1	-1.02	30
FUR4	-1.01	2.25
RRF1	-1.01	2.58
YAR1	-1.01	9.86
NOP56	-1.01	11.70
TEF2	-1.01	30
ZRG17	-1.00	2.20
VPS51	-1.00	2.39

MNL2	-1.00	3.06
PYC2	-1.00	5.20
OCA4	-1.00	7.09

a) -Log10 (p-value) greater than 30 is indicated as 30.

Table S5. YPH499/Co53 gene with increased expression level due to mutation

Gene or locus tag	Log2 (fold change)	Log10 (p-value)[a)]
SPG1	12.29	30
DPC7	9.12	30
SIP18	8.90	30
SPS100	8.77	30
RGI2	8.52	30
GRE1	7.97	30
ADY2	7.67	30
CTA1	7.21	30
FMP16	6.56	30
POT1	6.39	30
ACS1	5.79	30
NDE2	5.78	30
SPG4	5.71	30
HXT5	5.49	30
CSM4	5.43	30
FOX2	5.43	30
BOP2	5.33	30
PAI3	5.21	30
PAU19	5.11	30
PUT1	5.08	30
SRL4	5.04	30
ICY1	4.89	30
GDH3	4.82	30
MLS1	4.73	30
DAN4	4.66	30
ATP8	4.62	11.48
DDR2	4.54	30
FAT3	4.48	30
IZH4	4.47	30
ADH2	4.46	30
MSC1	4.46	30
POX1	4.42	30
HBT1	4.39	30
HEF3	4.35	30
PUT4	4.32	30
MEP1	4.25	30

LSO1	4.20	30
SHH4	4.17	30
JEN1	4.12	30
FMP48	4.07	30
MEP2	4.07	30
TKL2	4.04	30
BDH2	4.02	30
PHM7	4.02	30
UIP4	4.00	30
FMP45	3.91	30
COX26	3.90	30
UTR5	3.87	30
GSC2	3.86	30
MPC3	3.86	30
PDR10	3.86	30
ALD3	3.85	30
ARG82	3.82	30
RIM4	3.77	30
ALD4	3.75	30
PAL2	3.70	30
ATG32	3.67	30
PXA1	3.67	30
ATP6	3.66	30
ECM4	3.62	30
INO1	3.62	30
SFA1	3.62	30
GUT2	3.60	30
RTS3	3.60	30
DPA10	3.56	30
PRR2	3.53	30
ATO3	3.49	30
PLB2	3.49	30
PCK1	3.47	30
CMI8	3.44	30
CIS1	3.42	30
DAN1	3.39	30
GAP1	3.35	30
GPX1	3.35	30

ARE2	3.34	30
CRC1	3.34	30
DIP5	3.32	30
FKS3	3.32	30
ENA5	3.29	30
SNT309	3.28	30
IRC15	3.27	30
YAT1	3.26	30
SFC1	3.24	21.08
CIT3	3.24	30
OM45	3.24	30
15S_RRNA	3.23	30
CLD1	3.23	30
GTT1	3.23	30
ZTA1	3.22	30
CIT2	3.21	30
ICL1	3.21	30
SHC1	3.18	30
SDH6	3.13	30
PAU9	3.11	2.87
ATG34	3.10	30
UME6	3.09	23.10
ATG1	3.09	30
DCS2	3.09	30
SPG3	3.09	30
FAA4	3.08	30
STR3	3.08	30
CAT8	3.07	30
MDH2	3.07	30
UBC5	3.06	30
SPG5	3.05	30
GIP2	3.04	30
IDP3	3.04	30
YIG1	3.03	30
ALT2	3.01	30
SOK2	2.99	30
ECI1	2.98	30
BLM10	2.96	30

COB	2.93	30
GRX6	2.93	30
SEF1	2.93	30
KHA1	2.92	30
DBP1	2.91	30
MND1	2.90	20.26
CMC4	2.90	30
ENA2	2.90	30
MHO1	2.90	30
RSB1	2.90	30
PEX11	2.89	30
FMP49	2.88	13.92
ATG9	2.88	30
CUP2	2.88	30
RTN2	2.88	30
SPS19	2.88	30
APC11	2.87	30
MCO8	2.86	30
MSN2	2.86	30
PEX28	2.85	30
YGP1	2.85	30
RAD4	2.84	30
AAC1	2.83	30
ENA1	2.83	30
NUD1	2.83	30
SDH2	2.83	30
STB3	2.83	30
YPT53	2.83	30
OLE1	2.82	30
DDI1	2.81	30
RIM101	2.80	30
HFD1	2.78	30
RAD55	2.78	30
SLZ1	2.78	30
ARO10	2.77	30
EAR1	2.74	30
VHS2	2.74	30
MOH1	2.73	30

PPQ1	2.73	30
SWI1	2.73	30
YAT2	2.73	30
ISF1	2.72	30
DIN7	2.71	19.71
BI4	2.71	30
ISU1	2.71	30
AGP1	2.70	30
SPO20	2.70	30
CWC23	2.68	5.74
HVG1	2.67	13.22
NGR1	2.67	20.20
RGS2	2.67	30
AST2	2.66	21.62
CTF19	2.66	30
RRT1	2.65	30
YAP1	2.65	30
FBP1	2.64	10.34
GPG1	2.63	30
CCC1	2.62	30
BYE1	2.61	30
PSP1	2.60	30
TRX2	2.60	30
YOR1	2.60	30
UBX5	2.59	30
ALD2	2.57	30
CDC31	2.57	30
UGX2	2.56	30
AI5_ALPHA	2.55	30
COX1	2.55	30
CPR1	2.55	30
GIS1	2.55	30
NGL3	2.55	30
IRC13	2.54	18.90
GIP1	2.54	20.26
EDC2	2.54	30
LCL1	2.54	30
USV1	2.54	30

FMP23	2.53	13.45
ILT1	2.53	30
AI4	2.52	30
URA10	2.52	30
ATG36	2.51	20.34
JHD1	2.51	30
RPT6	2.51	30
ERV1	2.50	30
MIX17	2.50	30
MGR3	2.49	30
AI3	2.48	30
PTR2	2.48	30
AI2	2.47	30
MAG1	2.47	30
YCP4	2.47	30
BI2	2.46	30
CDC27	2.45	15.46
BSC2	2.45	30
PFK26	2.45	30
PHO4	2.45	30
OM14	2.44	20.77
PIL1	2.44	30
AFG3	2.43	30
GPT2	2.43	30
HXT4	2.43	30
HMS1	2.42	30
LGE1	2.42	30
PET20	2.42	30
PMP3	2.42	30
RCN2	2.42	30
SUE1	2.42	30
NFI1	2.41	30
SKS1	2.41	30
FZO1	2.40	13.01
FMP40	2.40	30
MEF2	2.40	30
NPR1	2.40	30
PRB1	2.39	30

RAD51	2.39	30
SHH3	2.39	30
SUR1	2.39	30
HHF1	2.38	30
NTH2	2.38	30
RPM1	2.38	30
ATG39	2.37	30
SUM1	2.37	30
SPF1	2.36	30
MIT1	2.35	6.69
SNA3	2.35	30
SSH4	2.35	30
PEX9	2.34	12.18
BDF2	2.34	30
DOC1	2.34	30
FMP52	2.34	30
BI3	2.33	30
COX7	2.33	30
HYR1	2.33	30
OTU1	2.33	30
NBP35	2.32	30
VPS27	2.32	30
OPY1	2.31	15.73
CRF1	2.31	30
FUB1	2.31	30
NRD1	2.31	30
SCS7	2.31	30
PEX22	2.30	20.22
AMS1	2.30	30
GLO1	2.30	30
ICL2	2.30	30
UBC8	2.30	30
BSC4	2.29	30
FDH1	2.29	30
LPX1	2.29	30
MUB1	2.29	30
CCS1	2.28	30
MDH3	2.28	30

CMR3	2.27	5.43
MPT5	2.27	15.34
ATG11	2.27	30
ATO2	2.27	30
SSM4	2.27	30
AI1	2.25	30
AHC1	2.24	12.53
SNF11	2.24	20.05
GDH2	2.24	30
GRX4	2.24	30
RRT8	2.24	30
YTA7	2.24	30
ATH1	2.23	30
KIC1	2.23	30
KNS1	2.23	30
MGA2	2.23	30
PDH1	2.23	30
SNC2	2.23	30
YAK1	2.23	30
TFC3	2.22	15.65
ARA1	2.22	30
IRC20	2.22	30
PDR12	2.22	30
PMC1	2.22	30
RPN8	2.22	30
LEE1	2.21	4.02
YPT11	2.21	9.00
RCF3	2.21	9.19
WAR1	2.20	16.77
NDL1	2.20	30
TPH3	2.20	30
FAA2	2.18	30
UBX4	2.17	11.01
HAL9	2.17	12.09
YSF3	2.17	13.19
CCL1	2.17	30
GYP7	2.17	30
HUA1	2.17	30

GPI2	2.16	15.54
BPT1	2.16	30
CSR2	2.16	30
RPN3	2.16	30
ZWF1	2.16	30
PRM3	2.15	8.25
GTO1	2.15	17.61
GIS3	2.15	18.24
ATG8	2.15	30
MPD2	2.15	30
RIB1	2.15	30
SNQ2	2.15	30
GTO3	2.13	4.57
HAA1	2.13	30
SIP2	2.13	30
RNR3	2.12	5.73
MIN8	2.12	30
MSC3	2.12	30
SYG1	2.12	30
TSC10	2.11	9.74
FAA1	2.11	30
FMS1	2.11	30
GPA2	2.11	30
MPO1	2.11	30
MCO32	2.10	8.84
YME2	2.10	30
PIG2	2.09	8.83
ARA2	2.09	12.35
GNP1	2.08	19.39
HHO1	2.08	30
RPT2	2.08	30
YET2	2.08	30
HYM1	2.07	11.38
APE3	2.07	30
CAT2	2.07	30
BRE4	2.06	30
GLC3	2.06	30
PEX30	2.06	30

PEX18	2.05	9.80
GPI10	2.05	14.59
CBF1	2.04	8.56
RSA1	2.04	13.80
GIM4	2.04	30
UBC13	2.04	30
VHS3	2.04	30
MRX8	2.03	10.11
MNC1	2.02	12.90
ATG4	2.02	16.86
CPS1	2.02	30
DAT1	2.02	30
UMP1	2.02	30
TES1	2.01	4.49
TDA11	2.01	10.18
BXI1	2.01	30
COX16	2.01	30
CSC1	2.01	30
NAR1	2.01	30
REH1	2.01	30
CAR2	2.00	30
MPM1	2.00	30
TAR1	2.00	30
WHI5	1.99	16.59
TMC1	1.99	30
MET8	1.98	3.79
AVT3	1.98	30
GET3	1.98	30
GLT1	1.98	30
NBL1	1.98	30
OLI1	1.98	30
PEX3	1.98	30
RRD1	1.98	30
SND1	1.98	30
ICT1	1.97	30
LSP1	1.97	30
HUB1	1.96	30
HUR1	1.96	30

MPE1	1.96	30
PDR1	1.96	30
SNC1	1.95	6.75
HRT1	1.95	30
PEX15	1.95	30
ATG14	1.94	2.09
TPK1	1.94	30
VID24	1.93	9.81
UGA2	1.93	14.92
FAS1	1.93	30
TGL3	1.93	30
TBS1	1.92	11.42
PRP31	1.92	11.57
AIM19	1.92	16.43
DIA1	1.92	30
KGD2	1.92	30
SAF1	1.91	10.23
ARO9	1.91	30
GPN2	1.91	30
PFU1	1.91	30
CTR3	1.90	5.04
IBA57	1.90	6.89
IDP2	1.90	7.93
GFD1	1.90	30
PMR1	1.90	30
PRE5	1.90	30
SSL2	1.90	30
TIM13	1.89	9.70
RAP1	1.89	14.52
MPC1	1.89	30
VFA1	1.88	4.18
MET30	1.88	30
PHB2	1.88	30
RTC6	1.88	30
XBP1	1.87	6.78
RRT6	1.87	9.73
NAB3	1.87	30
RAD54	1.87	30

SKP1	1.87	30
SKN7	1.86	9.03
LAP3	1.86	30
RKM5	1.85	5.42
CST6	1.85	14.73
ATG5	1.85	30
NUM1	1.85	30
SNX4	1.85	30
TFG2	1.85	30
DDP1	1.84	30
MRPL28	1.84	30
GSH1	1.83	17.04
SLM3	1.83	30
ULS1	1.83	30
HOT1	1.82	4.03
HIT1	1.82	12.34
COX23	1.82	12.76
NNK1	1.82	14.29
PRC1	1.82	30
VPS29	1.82	30
PTK1	1.81	5.35
ROY1	1.81	12.60
CSS3	1.81	30
HUL5	1.81	30
LUG1	1.80	6.20
RAD14	1.80	9.89
DEF1	1.80	30
TRS31	1.80	30
CIP1	1.79	3.40
CDC123	1.79	6.51
ADY4	1.79	9.07
STE24	1.78	15.02
PCP1	1.78	30
SHE10	1.78	30
ISR1	1.77	3.59
BUD23	1.77	30
MRPL16	1.77	30
PGA2	1.77	30

RTG1	1.77	30
TMA10	1.77	30
WTM1	1.77	30
SLO1	1.76	10.80
TGL4	1.76	15.13
CDC53	1.76	30
RPS30B	1.76	30
SPT2	1.76	30
CAF40	1.75	6.19
RTS2	1.75	8.52
MIN6	1.75	9.38
MAF1	1.75	13.49
ALP1	1.74	9.85
RRN3	1.74	13.77
RNA14	1.74	16.73
PGD1	1.73	6.80
SNG1	1.73	11.40
VPS55	1.73	30
AIM9	1.72	16.93
ISA1	1.72	30
FHN1	1.71	4.85
CDC23	1.71	6.96
NNF2	1.71	7.99
SEN2	1.71	8.51
GOS1	1.71	10.65
RFA3	1.71	10.86
SDH8	1.71	12.03
GLO4	1.71	30
PEP4	1.71	30
SSD1	1.71	30
SSU72	1.70	3.85
YPF1	1.70	11.27
BCP1	1.70	13.70
FMP46	1.70	30
LDB7	1.70	30
PMA2	1.69	3.94
SPC42	1.69	4.51
RRG1	1.69	5.52

PRP18	1.69	6.77
PDR8	1.69	8.26
ATG19	1.69	12.86
RFA1	1.69	13.08
MAP1	1.69	30
MNN5	1.69	30
SCH9	1.69	30
PXA2	1.68	7.37
SWF1	1.68	7.89
KEL1	1.68	8.56
SRC1	1.68	14.21
PTC1	1.68	15.65
FMP10	1.68	30
PBI2	1.68	30
QCR10	1.68	30
RRT15	1.68	30
YPR1	1.68	30
MVP1	1.67	9.51
GLC8	1.67	30
ISY1	1.67	30
OAZ1	1.67	30
NPC2	1.66	30
QCR6	1.66	30
NTO1	1.65	5.25
SHU2	1.65	5.67
QDR1	1.65	6.15
TRI1	1.65	7.01
PUT3	1.65	30
RVS161	1.65	30
PDC6	1.64	3.62
YTP1	1.64	5.40
EMP46	1.64	7.94
MKK1	1.64	15.57
CRP1	1.64	30
PCT1	1.64	30
PUT2	1.64	30
INP54	1.63	4.01
PTI1	1.63	4.11

TOD6	1.63	4.72
MDV1	1.63	7.05
SLX8	1.63	12.75
HSF1	1.63	14.26
UBC6	1.63	16.33
YRR1	1.62	4.99
RSN1	1.62	10.67
GAL83	1.62	14.56
CHZ1	1.62	30
KRE1	1.62	30
RPT1	1.62	30
SPO24	1.62	30
SUR2	1.62	30
CHK1	1.61	4.69
ECM10	1.61	4.71
DPC25	1.61	7.02
HTA2	1.61	7.33
ATG21	1.61	7.42
NCB2	1.61	11.42
DIC1	1.61	13.46
RPS27A	1.61	30
SWC5	1.60	5.73
CAB2	1.60	6.83
MRK1	1.60	9.69
NIS1	1.60	11.69
IMP2'	1.60	30
SLG1	1.60	30
FEX2	1.59	5.53
TUL1	1.59	8.01
WTM2	1.59	9.30
HTL1	1.59	10.07
CNL1	1.59	30
TVP18	1.59	30
CTK3	1.58	3.53
MNN14	1.58	7.95
SGT1	1.58	9.64
GRR1	1.58	10.25
PLB3	1.58	11.36

NAS6	1.58	12.11
APJ1	1.58	30
CCT8	1.58	30
ATG26	1.57	15.42
RPB4	1.57	30
MED7	1.56	5.12
PBA1	1.56	6.65
SPR6	1.56	8.23
CAB4	1.56	11.52
UBP13	1.56	15.96
PRE10	1.56	30
TPS2	1.56	30
SNA4	1.55	3.46
SPR1	1.55	3.57
VID27	1.55	11.26
DNF1	1.55	15.94
DLD3	1.55	30
GID8	1.55	30
SKN1	1.55	30
MOT2	1.54	5.18
MRX9	1.54	8.62
DPB4	1.54	10.40
MLO1	1.54	10.55
INO4	1.54	13.11
FET5	1.54	30
INH1	1.54	30
IWR1	1.54	30
NQM1	1.54	30
ORM2	1.54	30
STF1	1.54	30
HEL1	1.53	4.42
SAN1	1.53	8.98
FAP1	1.53	11.55
ACC1	1.53	30
FMT1	1.52	2.51
EMP65	1.52	4.62
PSH1	1.52	8.31
CCT3	1.52	30

CCT6	1.52	30
SOL2	1.52	30
GPI19	1.51	4.80
DAS1	1.51	5.58
MDM34	1.51	6.33
RAD16	1.51	9.32
ATG15	1.51	10.44
CWC24	1.51	10.54
TRS23	1.51	14.75
HPF1	1.51	30
RAD23	1.51	30
ATG31	1.50	2.65
RTG3	1.50	3.22
GAT2	1.50	3.84
MCO76	1.50	3.89
COS9	1.50	4.72
ECM27	1.50	5.23
SDP1	1.50	7.09
THI6	1.50	11.96
ECM21	1.50	12.86
RPN13	1.50	30
SHP1	1.50	30
MCM1	1.49	6.57
SMB1	1.49	10.69
CDC34	1.49	30
FIS1	1.49	30
IES6	1.49	30
SEM1	1.49	30
PEX5	1.48	7.92
TRR2	1.48	8.29
VTC1	1.48	8.98
RPC31	1.48	9.27
ORM1	1.48	10.17
YFH1	1.48	10.23
NUP2	1.48	13.52
DSC2	1.48	13.61
AI5_BETA	1.48	30
RDL1	1.48	30

SCS2	1.48	30
SPT15	1.48	30
TFB4	1.47	6.12
CDH1	1.47	6.25
ETR1	1.47	6.33
RRG9	1.47	8.16
DBP2	1.47	30
SAP155	1.47	30
ELA1	1.46	4.41
PEX35	1.46	5.23
PCI8	1.46	5.67
YFT2	1.46	8.75
COQ4	1.46	10.55
ZRT3	1.46	14.55
ESA1	1.46	15.10
ACO1	1.46	30
CDC37	1.46	30
PRE3	1.46	30
UBI4	1.46	30
VNX1	1.45	2.99
RAD30	1.45	3.97
TAD2	1.45	4.37
INO2	1.45	4.83
RMD5	1.45	10.73
SNF3	1.45	12.29
ADR1	1.45	30
AIM46	1.45	30
CCT5	1.45	30
GAC1	1.45	30
GRE2	1.45	30
MRS6	1.45	30
MUM2	1.44	3.55
SEN54	1.44	3.83
ASG1	1.44	4.07
BEM1	1.44	4.66
UBX7	1.44	8.56
PET100	1.44	12.51
PRE2	1.44	30

PUP2	1.44	30
TRX3	1.44	30
WHI3	1.43	2.57
IRA1	1.43	4.40
FYV6	1.43	8.01
FZF1	1.43	8.35
INA17	1.43	8.35
RFA2	1.43	14.80
COA6	1.43	30
COX15	1.43	30
HST1	1.43	30
PRE1	1.43	30
RPI1	1.42	3.63
LDO16	1.42	4.61
PEX31	1.42	5.08
SHR5	1.42	5.08
GAD1	1.42	8.51
ESL2	1.42	10.24
MET4	1.42	11.40
STE11	1.42	12.58
RPT3	1.42	30
IZH1	1.41	4.22
VPS72	1.41	4.58
JID1	1.41	4.87
YSA1	1.41	5.70
COQ21	1.41	7.25
MIH1	1.41	8.72
PKH2	1.41	9.69
UFD1	1.41	30
YTA12	1.41	30
BIG1	1.40	2.67
HSM3	1.40	3.45
AQY1	1.40	4.25
ATG20	1.40	9.20
MCH1	1.40	10.31
RPT4	1.40	30
CAF120	1.39	3.46
HPA2	1.39	3.65

RNH70	1.39	14.92
MCO10	1.39	30
RTK1	1.39	30
ATG33	1.38	2.10
RAS1	1.38	3.26
AZF1	1.38	3.92
VLD1	1.38	3.93
RSF1	1.38	5.39
EAF3	1.38	7.76
LSM5	1.38	9.52
MRPL51	1.38	11.27
UTR1	1.38	15.70
KTR1	1.38	30
PGA3	1.38	30
MSG5	1.37	3.11
SNF1	1.37	3.77
STE7	1.37	3.87
CFD1	1.37	6.90
ATG12	1.36	2.82
NCA3	1.36	2.97
OAF1	1.36	3.47
PET130	1.36	3.72
RMD1	1.36	4.33
ATG40	1.36	6.03
MXR1	1.36	7.22
RCR1	1.36	11.24
21S_RRNA	1.36	30
DBR1	1.35	3.28
ROG1	1.35	3.64
MAM3	1.35	9.10
ARG80	1.35	10.38
ERC1	1.35	12.49
UBP6	1.35	15.40
ARF2	1.35	30
MIC26	1.35	30
ABM1	1.34	2.67
SUT2	1.34	3.39
MPS3	1.34	3.92

MNE1	1.34	4.30
MIM2	1.34	4.38
MET28	1.34	6.14
IOC4	1.34	7.86
GRX3	1.34	9.19
RPN1	1.34	30
SAW1	1.33	2.73
VID30	1.33	6.18
ASK1	1.33	9.52
PEX19	1.33	10.71
NYV1	1.33	13.07
GUT1	1.33	30
LEU3	1.32	3.39
RAD3	1.32	4.87
PCL8	1.32	4.90
PAN6	1.32	6.12
TOP3	1.32	7.34
BOI2	1.32	7.54
NHP6A	1.32	7.58
CAP1	1.32	7.97
CDD1	1.32	8.00
BRE1	1.32	8.21
TIM21	1.32	15.40
DPL1	1.32	30
DSK2	1.32	30
GLN1	1.32	30
PRE7	1.32	30
PHO92	1.31	2.32
NHX1	1.31	6.04
HSV2	1.31	12.68
SNA2	1.31	13.85
SPT5	1.31	15.70
MTR2	1.31	30
NCE102	1.31	30
MNT4	1.30	2.11
AEP3	1.30	2.54
COA4	1.30	3.27
CDC16	1.30	3.59

BAG7	1.30	4.88
OSW7	1.30	5.88
VPS35	1.30	8.94
TOA1	1.30	9.42
GLO2	1.30	30
PRE9	1.30	30
YKT6	1.30	30
FUN19	1.29	2.41
YHC3	1.29	2.82
CDC8	1.29	4.16
HTZ1	1.29	5.82
GSM1	1.29	6.86
MSP1	1.29	9.74
IRA2	1.29	10.97
OYE3	1.29	11.57
RUB1	1.29	12.25
SSU1	1.29	13.34
VPS21	1.29	30
OAR1	1.28	3.17
TMS1	1.28	7.08
QNS1	1.28	8.60
SDD3	1.28	12.27
MIA40	1.28	30
RPS27B	1.28	30
ECM2	1.27	4.70
RAD1	1.27	7.07
SEG1	1.27	7.31
AFT2	1.27	7.46
FYV10	1.27	8.67
UBR1	1.27	13.40
COX3	1.27	30
GDI1	1.27	30
PYC1	1.27	30
HXT11	1.26	3.61
NFU1	1.26	4.58
ELP4	1.26	6.55
YIP3	1.26	6.77
RPO31	1.26	8.44

GLC7	1.26	30
KTR4	1.26	30
LPL1	1.26	30
SPP41	1.25	2.18
RAM1	1.25	2.40
PAH1	1.25	4.30
RRD2	1.25	5.23
PCL10	1.25	6.77
MDG1	1.25	7.66
RSC2	1.25	7.82
VPS62	1.25	9.51
AIM41	1.25	9.64
SPT6	1.25	10.31
RHO1	1.25	30
YRA1	1.25	30
PSO2	1.24	2.01
ISC10	1.24	2.18
SVL3	1.24	2.19
NUR1	1.24	4.39
SFT1	1.24	7.42
PBP4	1.24	13.05
ARB1	1.24	30
BMH1	1.24	30
PRE4	1.24	30
AIM18	1.23	3.49
UBX3	1.23	3.86
TAF13	1.23	4.49
SLM1	1.23	7.94
CTR2	1.23	10.59
OPI1	1.23	13.36
MNR2	1.23	30
TCP1	1.23	30
RCI50	1.22	3.39
SCD5	1.22	3.51
UFE1	1.22	5.51
SLM4	1.22	5.57
UGA1	1.22	6.60
MDH1	1.22	30

CPT1	1.21	4.27
FYV8	1.21	6.29
MFB1	1.21	6.85
TOM70	1.21	8.10
VMA10	1.21	10.11
FRQ1	1.21	10.57
MSN5	1.21	13.01
BAP2	1.21	30
CUZ1	1.21	30
OPY2	1.21	30
RTR2	1.20	2.18
MCY1	1.20	3.86
PET191	1.20	5.71
FLC2	1.20	9.21
TAF12	1.20	10.94
COX8	1.20	14.92
COA3	1.20	30
COX2	1.20	30
DAL80	1.19	2.08
DSL1	1.19	2.72
REF2	1.19	2.74
HXT9	1.19	3.07
RAV2	1.19	4.94
MKK2	1.19	7.07
SEC17	1.19	9.81
MIM1	1.19	13.62
PPH3	1.18	2.68
VPS24	1.18	5.51
YHC1	1.18	7.15
NCR1	1.18	9.79
ROX3	1.18	13.55
HTA1	1.18	30
OXP1	1.17	3.10
FEX1	1.17	3.14
PHO2	1.17	3.80
RPL18B	1.17	4.82
YNG2	1.17	6.66
TFA2	1.17	7.84

NOP16	1.17	7.90
RCI37	1.17	8.03
PDR16	1.17	11.80
BDF1	1.17	12.33
RPN7	1.17	15.70
RTR1	1.16	2.14
VID28	1.16	3.07
NAM7	1.16	4.07
RAD57	1.16	4.32
HEM2	1.16	4.97
NAM9	1.16	5.07
LRE1	1.16	5.13
ASR1	1.16	5.22
NEO1	1.16	6.30
COI1	1.16	6.53
PUP3	1.16	30
MOD5	1.15	2.10
MTC7	1.15	2.91
HFL1	1.15	3.62
RRN10	1.15	5.02
RTT103	1.15	5.47
FMP27	1.15	6.70
ALD6	1.15	6.85
CKB2	1.15	7.05
PSK1	1.15	8.85
RPN6	1.15	30
MED2	1.14	3.16
ULA1	1.14	3.59
GON7	1.14	4.13
YIM1	1.14	5.10
ASK10	1.14	6.32
VPS70	1.14	7.00
LYS20	1.14	8.68
EFT1	1.14	30
MRP8	1.14	30
SBA1	1.14	30
TMH11	1.14	30
SFL1	1.13	2.06

VPS3	1.13	2.14
VAB2	1.13	2.31
SHE9	1.13	2.33
MIN7	1.13	6.66
MAG2	1.13	7.41
SPT16	1.13	7.66
YPS1	1.13	8.42
ECM15	1.13	8.91
CCT2	1.13	30
COX13	1.13	30
SCL1	1.13	30
PFA3	1.12	2.16
SNU114	1.12	2.71
FHL1	1.12	3.86
MATALPHA2	1.12	4.25
CIN5	1.12	4.72
NHP6B	1.12	7.99
XRN1	1.12	30
PIP2	1.11	2.14
IBD2	1.11	2.80
PLC1	1.11	3.02
AGP2	1.11	3.70
MRM2	1.11	3.75
PAP2	1.11	4.36
RPH1	1.11	4.60
RSC4	1.11	4.75
COX11	1.11	4.79
BSD2	1.11	7.35
URH1	1.11	7.36
FPK1	1.11	9.68
NFS1	1.11	30
RNR2	1.11	30
RIT1	1.10	2.11
PIB2	1.10	2.48
STP1	1.10	3.35
TRM12	1.10	3.86
SRP54	1.10	10.51
SOD1	1.10	30

FOB1	1.09	2.26
RRN5	1.09	2.28
MGM1	1.09	3.86
UBC9	1.09	3.86
LEA1	1.09	4.39
GCD1	1.09	5.91
CSG2	1.09	9.37
MRPS18	1.09	10.10
SUA7	1.09	13.71
SDD4	1.08	2.32
SCC4	1.08	3.16
YUR1	1.08	4.11
ALO1	1.08	4.20
SIC1	1.08	4.77
TAF4	1.08	4.77
MXR2	1.08	5.06
TYE7	1.08	7.63
TOM22	1.08	11.16
SMX3	1.08	13.36
TYW3	1.07	2.15
EFM4	1.07	3.69
HRT3	1.07	4.38
VBA1	1.07	4.67
RSM22	1.07	4.91
RSM10	1.07	6.73
GOT1	1.06	2.30
ATP25	1.06	2.96
GPB2	1.06	3.08
GPI8	1.06	3.95
RER1	1.06	4.52
RQC1	1.06	4.53
TOS8	1.06	5.94
LSM3	1.06	5.98
SEC4	1.06	10.73
FBP26	1.06	12.81
RPB2	1.06	30
DPB11	1.05	2.22
CIN4	1.05	2.34

SPP1	1.05	3.28
THO2	1.05	3.67
LSM7	1.05	4.17
CDC40	1.05	5.00
PAN2	1.05	6.64
BMH2	1.05	8.04
ETP1	1.04	2.23
SNU56	1.04	2.26
HEM1	1.04	2.83
PIB1	1.04	4.40
ITT1	1.04	4.85
TRE2	1.04	5.86
STE13	1.04	6.00
CBP4	1.04	7.04
RPN5	1.04	30
ASI3	1.03	2.15
MRP2	1.03	2.54
MAL31	1.03	3.09
NCE101	1.03	5.10
PAR32	1.03	5.65
MRPL10	1.03	5.81
MDM32	1.03	6.27
UME1	1.02	2.71
ACK1	1.02	2.79
SYS1	1.02	3.79
WIP1	1.02	4.53
NPA3	1.02	7.33
PUP1	1.02	7.98
GDH1	1.02	10.41
SND3	1.02	14.49
PLN1	1.02	30
RAD50	1.01	2.07
RNQ1	1.01	2.92
TMT1	1.01	3.43
ERV2	1.01	3.73
CMC1	1.01	3.90
CDC36	1.01	4.42
APE4	1.01	4.87

CCT7	1.01	6.04
UCC1	1.01	8.41
QCR9	1.01	8.91
SET3	1.00	2.12
HRQ1	1.00	3.22
LIH1	1.00	3.94
CEG1	1.00	3.99
CMD1	1.00	4.62
SOM1	1.00	4.63
NST1	1.00	5.27
IPT1	1.00	5.50
GTR1	1.00	5.80
MRPL31	1.00	5.92
FTH1	1.00	7.07
ASN2	1.00	30
TIF35	1.00	30

a) -Log10 (p-value) greater than 30 is indicated as 30.

Table S6. YPH499/Co53 gene with decreased expression level due to mutation

Gene or locus tag	Log2 (fold change)	Log10 (p-value)[a)]
CUP1-2	-8.70	30
CUP1-1	-8.29	30
RGI1	-8.13	30
HSP30	-6.52	30
PDC1	-6.51	30
IRC7	-6.37	30
DCD1	-6.28	30
HXT3	-6.26	30
ULI1	-5.93	30
YPS6	-5.86	30
HSP150	-5.65	30
ARI1	-5.53	30
REE1	-5.15	30
SUR7	-5.14	30
CDC10	-5.00	30
CDC3	-4.91	30
OPI10	-4.78	30
MRX15	-4.75	30
NCW1	-4.73	30
AIM17	-4.70	30
SUC2	-4.68	30
RME1	-4.61	30
FIT2	-4.57	30
UBP11	-4.57	30
BFR1	-4.56	30
CYK3	-4.53	30
KIN3	-4.50	13.49
SSA4	-4.49	30
TKL1	-4.48	30
AAD3	-4.47	30
RAX2	-4.47	30
SRL1	-4.42	30
HXT14	-4.38	8.22
GCV1	-4.37	30
SAP4	-4.37	30
ATX2	-4.33	30

MVD1	-4.33	30
RDT1	-4.31	30
CIS3	-4.28	30
TOH1	-4.27	30
FAR1	-4.21	30
HXK2	-4.21	30
NTG1	-4.21	30
SAM1	-4.20	30
POF1	-4.12	16.44
XKS1	-4.12	30
ALT1	-4.11	30
DSF1	-4.08	30
EXG1	-4.08	30
HXK1	-4.04	30
CYS3	-4.03	30
TEM1	-4.02	30
THI12	-4.02	30
CLN1	-3.99	11.06
MSF1	-3.99	30
COS5	-3.94	30
EXG2	-3.93	30
CIR1	-3.89	30
HXT13	-3.86	30
MCM16	-3.85	10.72
CRN1	-3.82	30
ENO2	-3.80	30
TUB3	-3.80	30
CYB5	-3.77	30
IMD2	-3.77	30
AAC3	-3.76	6.90
PHR1	-3.76	30
MKC7	-3.69	30
COS2	-3.68	30
MLO50	-3.67	30
MAL12	-3.66	30
CLN2	-3.65	12.26
BUD4	-3.65	17.23
ERG4	-3.65	30

DAL1	-3.63	30
KIP2	-3.62	11.09
LYS9	-3.61	30
KAE1	-3.58	30
FKS1	-3.57	30
MAN2	-3.56	30
PUS4	-3.55	30
SRD1	-3.52	30
SUN4	-3.52	30
YIP5	-3.51	30
BTN2	-3.50	30
HOM3	-3.49	30
PNP1	-3.49	30
BBP1	-3.47	11.11
DOG2	-3.47	30
MAL32	-3.47	30
MIC60	-3.45	30
MIS1	-3.45	30
DIA2	-3.43	14.88
ADH7	-3.42	2.95
SWI5	-3.41	5.25
MYO4	-3.41	30
MME1	-3.40	30
AFR1	-3.39	30
FRM2	-3.39	30
SPO74	-3.39	30
LDS1	-3.38	5.06
ARO7	-3.37	30
CPR5	-3.36	30
HBN1	-3.35	10.40
SIL1	-3.35	30
YRF1-6	-3.35	30
CLB1	-3.34	9.18
GOR1	-3.34	30
HED1	-3.33	11.26
YRF1-1	-3.33	12.59
EGO4	-3.33	30
YRF1-3	-3.32	30

YRF1-8	-3.32	30
DCG1	-3.31	5.47
ARR3	-3.31	8.09
CLB2	-3.31	9.33
SCW4	-3.31	30
PPZ1	-3.29	30
YRF1-5	-3.29	30
ABP1	-3.28	30
COS3	-3.28	30
YHB1	-3.28	30
YRF1-7	-3.28	30
KEL3	-3.27	30
PMA1	-3.25	30
RBH2	-3.24	17.01
ARO8	-3.24	30
HAM1	-3.23	30
IMD4	-3.23	30
SKM1	-3.23	30
ERG26	-3.22	30
SER2	-3.22	30
TRM9	-3.22	30
YAP7	-3.21	15.43
HEM25	-3.21	30
IMA2	-3.21	30
PAN5	-3.21	30
RAI1	-3.21	30
YPK2	-3.20	30
RDS1	-3.19	11.65
YRF1-2	-3.19	16.93
CDC5	-3.19	30
THI5	-3.18	5.60
GAT4	-3.18	6.34
FRE3	-3.18	11.22
AMN1	-3.17	30
DSE4	-3.16	16.05
MCO14	-3.15	13.90
QDR2	-3.14	17.80
SMC2	-3.13	30

MRS3	-3.11	30
AXL2	-3.10	14.97
FPR4	-3.10	30
UTH1	-3.10	30
UTR2	-3.10	30
TAL1	-3.09	30
VTH1	-3.09	30
CLB4	-3.08	9.62
PAU6	-3.08	11.15
RPN14	-3.07	15.39
CRH1	-3.06	30
ADA2	-3.04	30
SIM1	-3.04	30
SMI1	-3.04	30
VMR1	-3.03	30
SPH1	-3.02	16.99
AHP1	-3.02	30
SCP160	-3.02	30
OSH6	-3.01	16.04
CTR1	-3.01	30
SSA2	-3.01	30
AGC1	-3.00	30
SAM4	-3.00	30
YHM2	-3.00	30
SPO77	-2.99	5.88
YPS5	-2.98	10.95
ADE17	-2.98	30
FRS1	-2.97	30
YRF1-4	-2.97	30
COQ2	-2.96	30
PER33	-2.96	30
AXL1	-2.95	13.67
BDH1	-2.95	30
OYE2	-2.95	30
ALK1	-2.94	6.25
PIR3	-2.94	30
PCL1	-2.92	6.15
GRX8	-2.91	10.24

ERG27	-2.91	30
CLN3	-2.90	2.24
SKT5	-2.90	30
VTH2	-2.90	30
RRT5	-2.89	4.82
SKG6	-2.89	6.10
MIN4	-2.89	14.90
SCW10	-2.89	30
SRO9	-2.88	10.34
RGA1	-2.88	30
SIA1	-2.88	30
ACP1	-2.87	30
NUP188	-2.87	30
DED81	-2.86	30
TPM1	-2.85	30
PER1	-2.84	30
GSP1	-2.83	30
NCP1	-2.83	30
SPT14	-2.83	30
FET4	-2.82	30
SMP1	-2.82	30
REX2	-2.81	30
TPS3	-2.81	30
BIO5	-2.80	30
HTS1	-2.80	30
PRM5	-2.80	30
PSD2	-2.80	30
STE4	-2.80	30
CSI2	-2.79	11.15
MPH2	-2.78	7.37
YPP1	-2.78	14.38
ERG6	-2.78	30
FRD1	-2.78	30
GSH2	-2.78	30
SRM1	-2.78	30
RCK1	-2.77	11.20
BIT61	-2.75	4.75
JLP2	-2.74	6.61

SSA1	-2.74	30
SRB2	-2.73	17.90
BUD7	-2.73	30
SEC61	-2.73	30
STD1	-2.73	30
TTI1	-2.72	8.18
DDI2	-2.72	8.48
RNH203	-2.72	30
SNZ2	-2.71	11.00
RBD2	-2.71	17.71
STE5	-2.71	30
YMD8	-2.71	30
HOF1	-2.70	4.42
CBT1	-2.70	30
MPH3	-2.69	12.14
DOT1	-2.68	7.96
SCO2	-2.68	14.75
MGL2	-2.68	30
NSA1	-2.68	30
RSR1	-2.68	30
ANB1	-2.67	14.80
GND1	-2.67	30
ATC1	-2.66	30
LYS4	-2.66	30
MYO1	-2.65	11.98
GAS3	-2.65	14.83
GCV2	-2.65	30
PAU15	-2.64	2.36
SHE1	-2.64	3.81
GIN4	-2.64	10.89
COQ3	-2.64	17.25
RTC4	-2.63	15.67
RRI2	-2.62	11.94
POP1	-2.62	30
TOS4	-2.61	2.35
SNO2	-2.59	11.01
GRE3	-2.59	30
HIP1	-2.59	30

SAC1	-2.59	30
TFC8	-2.58	8.58
MSW1	-2.58	13.27
ARC40	-2.58	30
GCV3	-2.58	30
PWP1	-2.58	30
SHE3	-2.57	4.88
GEP3	-2.57	10.28
BRP1	-2.57	14.00
MPC2	-2.57	30
POM33	-2.57	30
YSP2	-2.57	30
MEI4	-2.56	3.76
DUT1	-2.56	5.87
WSC3	-2.56	7.72
GPH1	-2.56	30
LYS1	-2.56	30
GAS5	-2.54	30
HTC1	-2.54	30
RRG7	-2.54	30
SPR3	-2.53	3.60
PRM10	-2.53	9.75
ADE1	-2.52	16.36
EHT1	-2.52	16.63
RKM2	-2.51	9.02
LDB17	-2.51	11.66
ALG3	-2.51	30
COS8	-2.51	30
ELO3	-2.51	30
SAE3	-2.50	5.11
PUL3	-2.50	7.73
SOL3	-2.50	9.03
URK1	-2.50	13.28
IQG1	-2.50	16.63
MAS2	-2.50	30
SOP4	-2.50	30
PRO1	-2.49	18.20
KTR2	-2.49	30

TRE1	-2.48	30
MDM10	-2.47	2.53
BDS1	-2.47	12.86
CTH1	-2.47	30
PAL1	-2.47	30
NUC1	-2.46	6.66
GPM3	-2.46	17.20
CUR1	-2.46	30
CHS3	-2.45	30
SES1	-2.45	30
TRK1	-2.45	30
NPY1	-2.44	10.64
GFD2	-2.44	11.68
SPE4	-2.44	13.20
ADH1	-2.44	30
EGH1	-2.43	5.58
DOT5	-2.43	30
ATF2	-2.42	7.23
MID2	-2.42	10.13
SSK22	-2.42	15.03
ADH6	-2.42	30
EMP24	-2.42	30
FTR1	-2.42	30
RRT12	-2.41	6.03
TIR3	-2.41	7.55
EGT2	-2.41	7.58
RTC3	-2.41	30
THR1	-2.41	30
CRG1	-2.40	30
PSA1	-2.40	30
PHO3	-2.39	4.24
MST1	-2.39	5.88
YVC1	-2.39	7.19
SHS1	-2.39	17.34
EXP1	-2.39	30
DIT2	-2.38	6.23
TIP1	-2.38	30
PUS6	-2.37	5.97

SRX1	-2.37	15.78
ANS1	-2.37	30
EMI2	-2.36	30
GCN1	-2.36	30
RUD3	-2.36	30
GFA1	-2.35	30
GUA1	-2.35	30
GDB1	-2.34	30
TRS120	-2.33	8.15
HGH1	-2.33	11.73
SNZ3	-2.33	11.99
PET8	-2.33	12.19
MDL1	-2.33	30
RPP2A	-2.33	30
MYO3	-2.32	11.45
KCH1	-2.31	30
MRPL7	-2.31	30
CDC45	-2.30	6.56
BNA3	-2.30	11.88
ASN1	-2.30	30
PRO3	-2.30	30
MYO5	-2.29	13.90
ERG20	-2.29	30
BAR1	-2.28	4.76
ADE6	-2.28	30
GNA1	-2.27	9.35
ARP5	-2.27	10.64
ECM31	-2.26	4.49
UPS3	-2.26	16.98
AAP1	-2.26	30
ATP2	-2.26	30
GEA2	-2.26	30
TAN1	-2.25	7.41
HIS2	-2.25	17.44
OSW1	-2.24	12.33
TGL1	-2.24	16.10
ERG11	-2.24	30
RPS22A	-2.24	30

AYT1	-2.23	3.32
RBS1	-2.23	9.29
FAL1	-2.22	6.99
TDH2	-2.22	30
HUG1	-2.21	6.83
RPC25	-2.21	9.19
PRS4	-2.21	12.76
APE1	-2.21	30
BUD14	-2.21	30
LSB1	-2.21	30
SKG1	-2.21	30
FDO1	-2.20	4.77
KAR1	-2.20	6.24
KTI12	-2.20	14.70
BNA1	-2.20	30
GRS1	-2.20	30
SRP101	-2.20	30
EDS1	-2.19	3.91
POL1	-2.19	4.75
ARH1	-2.19	11.19
MRPL25	-2.19	18.67
SIP3	-2.18	3.95
AIM44	-2.18	4.47
ERD2	-2.18	16.61
MCM5	-2.18	18.69
SUT1	-2.17	2.43
COG8	-2.17	8.01
SAH1	-2.17	30
ARP1	-2.16	5.41
DAL7	-2.16	12.57
CUP9	-2.16	13.78
HDA1	-2.16	30
TUB1	-2.16	30
EPT1	-2.15	5.69
SNO3	-2.15	8.62
DOP1	-2.15	15.46
CYC1	-2.15	30
MYO2	-2.15	30

ODC2	-2.15	30
RPL18A	-2.15	30
BCH1	-2.14	10.06
FRE1	-2.14	11.49
ACO2	-2.14	30
DOA1	-2.14	30
SVS1	-2.13	4.81
ARG7	-2.13	30
ERG1	-2.13	30
PFK27	-2.13	30
TSA1	-2.13	30
VMA22	-2.12	5.28
ERG13	-2.12	30
PST1	-2.12	30
MAK10	-2.10	7.59
KDX1	-2.10	14.68
HSP10	-2.10	30
RPS17B	-2.10	30
MOB2	-2.09	4.09
FSH3	-2.09	7.63
TAF11	-2.09	10.75
CAR1	-2.09	30
CCW12	-2.09	30
TPO2	-2.09	30
MRS1	-2.08	9.27
MRX4	-2.08	13.17
ALR2	-2.08	30
SMF1	-2.08	30
TOS3	-2.07	6.15
BOL3	-2.07	6.60
SSO2	-2.07	13.47
APM4	-2.07	30
COP1	-2.07	30
LSC2	-2.07	30
PFY1	-2.07	30
TIF4632	-2.07	30
TOS1	-2.07	30
GIC1	-2.06	4.75

RFC5	-2.06	5.52
IMA5	-2.06	5.75
GUS1	-2.06	30
KRS1	-2.06	30
NOP58	-2.06	30
DSF2	-2.05	2.22
BNI4	-2.05	12.36
CRS1	-2.05	15.44
ADE3	-2.05	30
EMC1	-2.05	30
GAR1	-2.05	30
QCR2	-2.05	30
SNZ1	-2.05	30
OTU2	-2.04	12.44
HXT1	-2.04	30
LYS12	-2.04	30
PMT2	-2.04	30
TYS1	-2.04	30
PHO91	-2.03	3.20
DAS2	-2.03	7.42
HXT8	-2.03	11.67
CHS7	-2.03	30
SSB1	-2.03	30
NUP84	-2.02	12.99
NUP145	-2.02	16.84
EMA19	-2.02	18.02
AQY3	-2.02	30
EGD2	-2.02	30
URA6	-2.02	30
SEC31	-2.01	17.77
RPC82	-2.01	19.07
DUG1	-2.01	30
GAL80	-2.01	30
SEC28	-2.01	30
TCD2	-2.01	30
ECL1	-2.00	3.62
PBI1	-2.00	4.69
CTF13	-2.00	5.32

BIK1	-2.00	9.51
RCM1	-2.00	17.82
ACT1	-2.00	30
RIM21	-2.00	30
YPD1	-2.00	30
SPO13	-1.99	2.21
SDD1	-1.99	6.90
TWF1	-1.99	7.40
ACA1	-1.99	15.53
ARC19	-1.99	30
RPL11B	-1.99	30
RAX1	-1.98	6.59
ERG5	-1.97	16.07
MDL2	-1.97	19.09
FET3	-1.97	30
GAL10	-1.96	2.44
NEJ1	-1.96	3.81
SLD3	-1.96	7.73
SNL1	-1.96	8.43
UTP10	-1.96	13.08
STU2	-1.95	9.86
CDC28	-1.95	12.55
FMP33	-1.95	15.65
PHO13	-1.95	30
RPS18B	-1.95	30
SRL3	-1.95	30
URA2	-1.95	30
AFT1	-1.94	4.50
HXT16	-1.94	4.55
MRPL32	-1.94	7.04
FRE7	-1.94	8.37
MCD4	-1.94	14.77
ACS2	-1.94	30
HXT7	-1.94	30
NDT80	-1.93	3.61
CLP1	-1.93	4.65
SET4	-1.93	5.96
MRX1	-1.93	8.30

APL3	-1.92	5.00
NAT1	-1.92	16.42
KRE6	-1.92	30
RPS28B	-1.92	30
MRC1	-1.91	5.65
IFA38	-1.91	7.33
SEC62	-1.91	30
IRC10	-1.90	5.32
SED4	-1.90	7.85
SER3	-1.90	30
MSH2	-1.89	9.61
NUG1	-1.89	30
ACE2	-1.88	2.31
EST2	-1.88	2.42
PIF1	-1.88	7.79
CDA1	-1.87	4.12
APC5	-1.87	4.48
PML39	-1.87	7.19
TRM11	-1.87	8.97
GGC1	-1.87	30
RPL22A	-1.87	30
VPS41	-1.86	5.61
THI22	-1.86	7.32
BSC5	-1.86	8.09
MTO1	-1.86	11.44
PMU1	-1.86	30
TRR1	-1.86	30
IRC8	-1.85	4.32
THI80	-1.85	4.41
COX5B	-1.85	30
GTT2	-1.85	30
MMT1	-1.84	6.06
MSY1	-1.84	9.22
PMT5	-1.84	11.32
PHD1	-1.84	17.63
ARP2	-1.84	30
RPL15A	-1.84	30
UTP15	-1.83	17.49

ADD37	-1.83	30
RPL23B	-1.83	30
SEC27	-1.83	30
SRB7	-1.82	7.67
VAC17	-1.82	8.59
ERG8	-1.82	9.64
MCH5	-1.82	30
MLC1	-1.82	30
TUB2	-1.82	30
AIM34	-1.81	5.16
URB2	-1.81	5.31
GPA1	-1.81	5.41
PRR1	-1.81	7.02
SEC59	-1.81	8.09
DEG1	-1.81	8.22
LOA1	-1.81	9.34
DBF2	-1.81	10.06
CCW14	-1.81	18.67
SMF2	-1.81	30
YHP1	-1.80	2.78
DAL5	-1.80	5.60
SOR1	-1.80	13.10
DOG1	-1.80	13.51
RPL2B	-1.80	30
YSC84	-1.80	30
PAU23	-1.79	5.34
ORT1	-1.79	9.77
CCA1	-1.79	9.93
CHC1	-1.79	30
GUK1	-1.79	30
SPC24	-1.78	3.09
VAM6	-1.78	6.07
KTR3	-1.78	9.26
RIA1	-1.78	13.08
ECM33	-1.78	19.52
RPL2A	-1.78	30
MET10	-1.77	5.63
NMT1	-1.77	15.28

MRH1	-1.77	30
SEC21	-1.77	30
PAC11	-1.76	3.84
RRF1	-1.76	5.48
MUK1	-1.76	15.67
SPT4	-1.76	30
ROF1	-1.75	2.35
ACF4	-1.74	2.23
RAD59	-1.74	5.35
BET5	-1.74	6.82
PDI1	-1.74	30
HIS6	-1.73	8.24
SOR2	-1.73	13.58
DPH2	-1.73	18.22
ALD5	-1.73	30
CPR6	-1.73	30
MST27	-1.72	3.70
DAL4	-1.72	3.78
CTF18	-1.72	3.96
HXT15	-1.72	5.71
RAM2	-1.72	15.38
RPS20	-1.72	30
YEF3	-1.72	30
POL32	-1.71	2.93
VPS36	-1.71	9.41
ENT1	-1.71	9.70
LAP2	-1.71	10.36
INP53	-1.71	12.86
ATG41	-1.71	15.40
RPP0	-1.71	30
SSE1	-1.71	30
PGM1	-1.70	4.87
TAE1	-1.70	6.63
SFB3	-1.70	15.23
ROM1	-1.70	17.14
CYC7	-1.70	30
NCW2	-1.70	30
BUD9	-1.69	4.51

SWE1	-1.69	9.40
SNF8	-1.69	17.80
DIT1	-1.68	2.68
CLA4	-1.68	4.84
YVH1	-1.68	4.84
COG4	-1.68	8.47
ALG6	-1.68	8.92
CYC2	-1.68	9.32
YEH1	-1.68	11.52
SNO1	-1.68	30
COG3	-1.67	3.92
MON2	-1.67	8.38
YET3	-1.67	30
URB1	-1.66	5.86
SAP185	-1.66	11.94
TEF4	-1.66	13.49
AIM45	-1.66	17.14
RPL9A	-1.66	30
ACF2	-1.65	6.22
PLP2	-1.65	8.57
KIN4	-1.65	9.58
LTV1	-1.65	11.22
URA5	-1.65	30
MCD1	-1.64	3.57
MSE1	-1.64	4.31
KAR3	-1.64	4.81
RPS11B	-1.64	6.90
SCP1	-1.64	7.04
KTR5	-1.64	9.52
DPP1	-1.64	10.01
TCB2	-1.64	10.04
POL5	-1.64	13.30
BNA6	-1.64	16.51
CHA1	-1.64	30
LMO1	-1.63	2.51
AZR1	-1.63	2.59
AIM10	-1.63	3.77
MAK11	-1.63	6.32

ERR1	-1.63	8.12
PUS1	-1.63	16.66
HO	-1.62	2.36
NAT5	-1.62	8.52
RRB1	-1.62	18.02
MRT4	-1.62	30
RPL34A	-1.62	30
RPS12	-1.62	30
STT3	-1.62	30
XDJ1	-1.62	30
ASH1	-1.61	2.76
TEX1	-1.61	3.12
SKI2	-1.61	8.62
SGA1	-1.61	12.38
PIR1	-1.61	15.37
GDE1	-1.61	16.18
RPS8B	-1.61	30
ALK2	-1.60	3.52
DRS2	-1.60	4.56
BUD3	-1.60	6.55
ELO1	-1.60	6.97
MVB12	-1.60	8.01
CKI1	-1.60	10.90
TPA1	-1.60	13.54
SPC29	-1.59	3.75
RTT107	-1.59	4.98
IPK1	-1.59	6.39
CAF16	-1.59	13.54
LTP1	-1.59	14.14
CWH43	-1.59	30
GUP2	-1.58	2.22
ECO1	-1.58	4.12
SEC1	-1.58	5.11
SWI6	-1.58	6.90
RDH54	-1.58	9.18
HAT2	-1.58	15.13
SAM2	-1.58	16.76
AQR1	-1.58	18.45

TOM40	-1.58	18.76
RPL11A	-1.58	30
TAF8	-1.57	5.30
AST1	-1.57	5.34
RFC1	-1.57	6.09
AVT1	-1.57	11.41
CYC8	-1.56	2.97
HCS1	-1.56	3.32
NMA1	-1.56	3.77
LOT6	-1.56	3.95
ALG1	-1.56	4.15
AVT5	-1.56	4.30
ARG2	-1.56	6.52
SET5	-1.56	8.98
GAS1	-1.56	30
RPL12A	-1.56	30
RSC3	-1.55	4.63
CDC14	-1.55	5.71
RSF2	-1.55	6.08
TRM2	-1.55	7.21
MPD1	-1.55	8.20
CDC12	-1.55	11.33
NNF1	-1.54	2.41
DBP6	-1.54	4.14
YCS4	-1.54	4.30
ARF3	-1.54	5.21
PXL1	-1.54	5.98
NUT1	-1.54	7.06
PRM8	-1.54	7.21
DRS1	-1.54	13.89
HSP78	-1.54	30
KAR2	-1.54	30
ZRT1	-1.54	30
OGG1	-1.53	4.41
SPO75	-1.53	4.67
SLD7	-1.53	7.71
MRM1	-1.53	30
TDH3	-1.53	30

PES4	-1.52	4.93
NGL2	-1.52	6.81
SEC16	-1.52	7.27
ERR2	-1.52	7.58
ERG28	-1.52	14.25
RPL42A	-1.52	30
RKM4	-1.51	6.68
FCY1	-1.51	30
IPL1	-1.50	3.22
GDA1	-1.50	7.29
TRP2	-1.50	8.53
NOP2	-1.50	10.16
RPO26	-1.50	12.66
PUG1	-1.49	2.63
RFU1	-1.49	2.66
PET112	-1.49	2.75
TLG1	-1.49	3.79
UBP16	-1.49	3.94
PRP5	-1.49	4.39
FDC1	-1.49	4.72
UBP8	-1.49	6.34
GEF1	-1.48	5.27
PTM1	-1.48	12.16
SEC11	-1.48	14.42
MEX67	-1.48	30
GYL1	-1.47	2.23
UIP3	-1.47	4.98
SPR28	-1.47	7.03
MCM2	-1.47	9.72
RFS1	-1.47	14.28
SEC23	-1.47	16.12
TRM3	-1.46	4.01
MED1	-1.46	4.32
PSD1	-1.46	6.42
ERP2	-1.46	6.51
PPN2	-1.46	7.65
TRS65	-1.46	7.77
ADO1	-1.46	30

MSM1	-1.45	2.55
CIN2	-1.45	3.30
HIM1	-1.45	4.29
MMP1	-1.45	4.82
MTF2	-1.45	5.32
ORC1	-1.45	7.55
SNU13	-1.45	8.36
SHE2	-1.45	9.31
EMW1	-1.45	10.40
RRP3	-1.45	13.40
MES1	-1.45	19.11
GAS2	-1.44	3.98
AAD4	-1.44	4.31
YSP3	-1.44	5.92
APL6	-1.44	8.47
FPR1	-1.44	30
GSY2	-1.44	30
HOM2	-1.44	30
MTM1	-1.44	30
PCM1	-1.44	30
SEC63	-1.44	30
RMD9	-1.43	5.47
EFR3	-1.43	6.79
RPL20B	-1.43	30
STV1	-1.43	30
RRM3	-1.42	4.31
NTE1	-1.42	5.27
DPH6	-1.42	5.47
CPD1	-1.42	6.67
PAM18	-1.42	13.44
ADE8	-1.42	30
AIM24	-1.41	3.42
SEC10	-1.41	4.69
RRP1	-1.41	5.35
YCG1	-1.41	5.47
SIN4	-1.41	5.51
ECM18	-1.41	5.65
TAF2	-1.41	5.86

NDJ1	-1.41	7.09
TUM1	-1.41	7.12
HER2	-1.41	7.49
RHO2	-1.41	7.82
THR4	-1.41	12.65
LCB5	-1.41	13.11
RPS1B	-1.41	30
RPS23A	-1.41	30
SED1	-1.41	30
SHM2	-1.41	30
CSH1	-1.40	2.12
SWD2	-1.40	3.65
PHS1	-1.40	4.42
CEX1	-1.40	4.78
ROD1	-1.40	6.15
MIN9	-1.40	6.27
NUP85	-1.40	7.04
CDC11	-1.40	9.19
SME1	-1.40	12.61
ARO4	-1.40	30
KRE9	-1.40	30
DUG2	-1.39	2.97
ATM1	-1.39	3.60
MAD1	-1.39	5.21
NDC1	-1.39	7.38
MIC19	-1.39	7.71
MPH1	-1.39	7.80
SYM1	-1.39	9.42
NIT3	-1.39	11.93
MRP51	-1.39	13.41
ARO1	-1.39	30
RPS21B	-1.39	30
SIR2	-1.38	2.75
STU1	-1.38	3.96
EPO1	-1.38	6.77
PKR1	-1.38	7.98
RPL6A	-1.38	11.36
ARP3	-1.38	15.15

RPL8A	-1.38	30
UGP1	-1.38	30
FLO5	-1.37	3.07
CTS2	-1.37	4.13
ISM1	-1.37	4.26
RRN11	-1.37	5.51
PPS1	-1.37	7.46
SPB1	-1.37	8.66
RPL26A	-1.37	16.53
HOL1	-1.37	18.14
IDP1	-1.37	30
RPL39	-1.37	30
THS1	-1.37	30
VMA16	-1.37	30
RKM3	-1.36	2.13
PRP42	-1.36	4.16
URA1	-1.36	11.64
BGL2	-1.36	30
RPS0A	-1.36	30
COS7	-1.35	2.23
AIM14	-1.35	5.72
RPP1	-1.35	5.86
YOP1	-1.35	11.56
CDC19	-1.35	30
RPL12B	-1.35	30
NCL1	-1.34	8.17
HSL1	-1.34	11.15
DPC29	-1.34	11.31
RPL8B	-1.34	30
RPS5	-1.34	30
ERG29	-1.33	2.62
CGI121	-1.33	2.74
BAP3	-1.33	8.05
STE23	-1.33	9.26
WRS1	-1.33	13.30
RTA1	-1.32	2.84
VPS75	-1.32	5.45
PPA2	-1.32	7.32

UTP13	-1.32	7.77
EMG1	-1.32	12.67
RGT1	-1.32	30
RPL16B	-1.32	30
RRP5	-1.32	30
YUH1	-1.31	3.06
SEC3	-1.31	8.41
MAD2	-1.31	8.54
TRA1	-1.31	9.07
AGX1	-1.31	13.16
PUN1	-1.31	20.00
SLT2	-1.31	30
ZUO1	-1.31	30
MON1	-1.30	2.98
ZRG17	-1.30	3.36
STE6	-1.30	4.07
CHL1	-1.30	5.42
MTQ1	-1.30	6.01
OMS1	-1.30	6.94
RCF1	-1.30	9.77
SDH4	-1.30	16.15
NAF1	-1.29	6.35
TVP15	-1.29	8.52
END3	-1.29	11.03
ADK1	-1.29	30
SAL1	-1.28	3.32
GCS1	-1.28	5.06
DNF2	-1.28	10.09
RPS24B	-1.28	30
TRP5	-1.28	30
HMI1	-1.27	2.30
ARL1	-1.27	2.47
CBP2	-1.27	3.10
SEC12	-1.27	4.94
RPA12	-1.27	7.17
IRC22	-1.27	10.19
RPA49	-1.27	10.31
SUP45	-1.27	15.21

PDE1	-1.27	30
RPS29A	-1.27	30
REV7	-1.26	2.80
SWI4	-1.26	3.10
PHO12	-1.26	3.14
TDA8	-1.26	3.66
PUF4	-1.26	5.13
DUN1	-1.26	5.85
CSE4	-1.26	6.80
PUS7	-1.26	7.99
RPL42B	-1.26	30
RCY1	-1.25	2.52
TOF1	-1.25	2.93
AIM26	-1.25	3.05
STE20	-1.25	4.26
SAE2	-1.25	4.81
MID1	-1.25	8.09
PAM1	-1.25	8.36
YSC83	-1.25	9.77
VPS13	-1.25	10.20
COM2	-1.25	12.63
EGD1	-1.25	30
YRO2	-1.25	30
IME4	-1.24	2.07
BUD2	-1.24	2.86
RTC1	-1.24	2.96
CIK1	-1.24	3.47
GPI16	-1.24	8.35
NOT5	-1.24	9.33
GCN20	-1.24	12.13
GAT1	-1.23	3.35
GCD10	-1.23	4.40
ANY1	-1.23	13.37
NOP56	-1.23	15.99
RPP1B	-1.23	30
SAC6	-1.23	30
CAX4	-1.22	2.31
CNE1	-1.22	2.34

STE50	-1.22	4.14
SDO1	-1.22	6.12
RNT1	-1.22	6.28
NIT1	-1.22	6.53
SEC7	-1.22	7.05
UTP21	-1.22	7.17
PEX6	-1.21	3.62
SEC65	-1.21	6.51
PMT1	-1.21	20.24
VHT1	-1.20	4.09
EMC5	-1.20	6.15
HXT6	-1.20	30
ICS2	-1.19	2.18
TRL1	-1.19	2.82
PRP2	-1.19	3.02
VPS16	-1.19	3.97
SKI8	-1.19	4.27
HAP5	-1.19	4.54
LNP1	-1.19	5.02
CCH1	-1.19	6.33
LIP5	-1.19	8.92
BOL2	-1.19	19.59
PIC2	-1.19	30
TNA1	-1.19	30
ATP23	-1.18	4.30
SMC4	-1.18	5.36
FPR2	-1.18	5.38
PYC2	-1.18	6.51
CMP2	-1.18	6.58
HRI1	-1.18	18.71
RPS13	-1.18	30
NMD4	-1.17	2.76
CDC9	-1.17	3.96
GEP4	-1.17	5.12
TRM82	-1.17	5.48
BUD6	-1.16	2.92
COX10	-1.16	4.84
RPG1	-1.16	5.41

RRP45	-1.16	6.14
PRP19	-1.16	7.04
FCY21	-1.16	7.15
DPB2	-1.16	8.02
RLP24	-1.16	10.89
RPP2B	-1.16	30
PAC1	-1.15	2.12
PEP5	-1.15	2.40
USB1	-1.15	2.44
COG2	-1.15	3.63
EAF7	-1.15	3.84
YMC1	-1.15	4.14
SAM50	-1.15	4.18
ZDS1	-1.15	4.43
SMC3	-1.15	4.44
AIM7	-1.15	6.16
NUS1	-1.15	7.20
TPN1	-1.15	11.01
SRY1	-1.15	12.04
MEP3	-1.15	18.10
RPL1B	-1.15	30
VPS63	-1.14	2.50
KAP122	-1.14	3.22
CWP2	-1.14	3.70
MLC2	-1.14	5.36
MRPL11	-1.14	5.47
SHM1	-1.14	7.46
TMA20	-1.14	8.35
RFT1	-1.14	8.78
PTP2	-1.14	10.36
ECM7	-1.14	11.23
RPL13A	-1.14	13.86
FSH1	-1.14	16.65
PCL5	-1.14	30
RPS26B	-1.14	30
TMA19	-1.14	30
TMN2	-1.13	2.10
ABZ2	-1.13	4.16

VAN1	-1.13	6.16
PIN2	-1.13	6.37
SLK19	-1.13	9.43
GLR1	-1.13	12.57
RPL35A	-1.13	30
ROG3	-1.12	3.59
TRZ1	-1.12	3.69
GCD14	-1.12	4.09
CTP1	-1.12	4.95
CKA1	-1.12	11.73
NFT1	-1.11	2.66
ALG5	-1.11	2.93
UPF3	-1.11	4.77
MNT2	-1.11	4.82
RIO2	-1.11	5.01
NOP53	-1.11	5.70
NUP170	-1.11	5.73
PTC2	-1.11	5.79
OCA4	-1.11	8.30
FUM1	-1.11	11.78
FAB1	-1.10	2.65
EMA17	-1.10	3.07
POL3	-1.10	3.77
AAD10	-1.10	6.84
EBP2	-1.10	8.46
GIM3	-1.10	8.52
ATP1	-1.10	19.14
HCH1	-1.10	30
RPS31	-1.10	30
PRP28	-1.09	2.08
CIA1	-1.09	2.69
SLX9	-1.09	2.96
ELM1	-1.09	3.16
NUP133	-1.09	5.51
FRS2	-1.09	5.99
MIC10	-1.09	8.92
YAR1	-1.09	11.10
ATP5	-1.09	12.49

RET3	-1.09	14.28
VRP1	-1.08	2.08
GIM5	-1.08	2.15
MMR1	-1.08	3.67
NUP57	-1.08	7.01
DIP2	-1.08	7.14
TAT2	-1.08	11.04
IML2	-1.08	11.36
ERG2	-1.08	18.50
POR1	-1.08	30
PEP3	-1.07	2.89
SGO1	-1.07	3.50
MRP1	-1.07	6.89
YKE2	-1.07	8.97
PET9	-1.07	10.80
CBF5	-1.07	18.53
PMT6	-1.06	2.63
SER33	-1.06	5.67
RBK1	-1.06	8.48
RPL21A	-1.06	12.73
POL2	-1.05	2.37
HRD3	-1.05	3.43
TVP38	-1.05	4.20
CTL1	-1.05	4.47
RPL6B	-1.05	5.00
ISU2	-1.05	7.91
RPL31A	-1.05	14.91
FLO10	-1.04	2.03
POP5	-1.04	2.25
MNL2	-1.04	3.15
NUP82	-1.04	3.36
STE14	-1.04	3.84
CDC7	-1.04	5.17
MCM4	-1.04	7.16
GPI17	-1.04	9.71
HIS5	-1.04	13.10
SEC24	-1.04	15.09
GEA1	-1.03	2.43

WHI4	-1.03	2.44
NRP1	-1.03	2.59
RLF2	-1.03	3.07
EMP70	-1.03	3.75
LRO1	-1.03	4.84
BUD17	-1.03	6.43
ECM16	-1.03	7.39
SUP35	-1.03	19.20
BZZ1	-1.02	3.17
PCL9	-1.02	4.05
CYB2	-1.02	4.50
BCS1	-1.02	4.61
ERG12	-1.02	5.12
ATF1	-1.02	5.82
TIF11	-1.02	30
NRK1	-1.01	2.64
PTC4	-1.01	3.21
MMM1	-1.01	3.27
SAP190	-1.01	4.99
LSC1	-1.01	9.16
PGK1	-1.01	30
FMP30	-1.00	3.10
OST4	-1.00	6.29
TPM2	-1.00	6.49
ADE12	-1.00	12.29
RPS19B	-1.00	18.69
YKE4	-1.00	21.15

a) -Log10 (p-value) greater than 30 is indicated as 30.

Table S7. YPH499/Co58 gene with increased expression level due to mutation.

Gene or locus tag	Log2 (fold change)	Log10 (p-value)[a)]
SPG1	12.67	30
SIP18	9.59	30
DPC7	9.28	30
RGI2	8.77	30
SPS100	8.65	30
GRE1	8.38	30
ADY2	7.71	30
FMP16	7.38	30
CTA1	7.37	30
POT1	6.95	30
NDE2	6.31	30
ACS1	6.19	30
SPG4	6.19	30
PAI3	5.86	30
LSO1	5.75	30
BOP2	5.74	30
FOX2	5.43	30
PUT1	5.37	30
HXT5	5.31	30
DDR2	5.30	30
CSM4	5.27	30
SHH4	5.11	30
SRL4	5.03	30
ICY1	4.99	30
HBT1	4.97	30
GDH3	4.84	30
MSC1	4.76	30
ATP8	4.75	8.72
ARG82	4.56	30
FMP45	4.53	30
UIP4	4.49	30
ALD3	4.40	30
ADH2	4.38	30
PAU19	4.37	7.85
RTS3	4.37	30
HEF3	4.35	30

TKL2	4.33	30
MEP1	4.31	30
POX1	4.30	30
PAU8	4.25	0.18
ECM4	4.19	30
COX26	4.18	30
JEN1	4.16	30
PHM7	4.14	30
BDH2	4.11	30
FAT3	4.09	30
TAR1	4.07	30
GTT1	4.06	30
NRD1	4.04	30
15S_RRNA	3.95	30
MPC3	3.95	30
MATALPHA1	3.92	0.10
MLS1	3.92	30
GUT2	3.88	30
OM45	3.86	30
PAL2	3.86	30
ALD4	3.85	30
GPI2	3.82	30
FMP48	3.81	30
PRR2	3.80	30
SFA1	3.80	30
RIM4	3.78	30
SNT309	3.72	30
UTR5	3.66	21.31
PXA1	3.64	30
CIS1	3.60	30
CMI8	3.59	30
PDR10	3.58	30
RTN2	3.57	30
SHH3	3.57	30
ATP6	3.56	30
MDH2	3.55	30
SEF1	3.54	30
ATG32	3.52	30

RRT15	3.52	30
UBC5	3.52	30
CRC1	3.50	30
MND1	3.45	30
CWC23	3.44	10.58
CIT2	3.44	30
CPR1	3.42	30
ATO3	3.41	30
DCS2	3.38	30
GPX1	3.36	30
SLZ1	3.35	30
IRC15	3.34	30
DAN4	3.32	12.74
ECI1	3.32	30
MSN2	3.32	30
SPG5	3.32	30
YAT1	3.32	30
FBP1	3.31	17.87
TRX2	3.29	30
SEN2	3.28	30
GSC2	3.27	30
GRX6	3.26	30
MCO8	3.25	30
SHC1	3.24	30
CDC31	3.23	30
NUD1	3.22	30
APC11	3.21	30
INO1	3.19	30
STR3	3.19	30
ARE2	3.18	30
GTO1	3.18	30
PUT4	3.18	30
YIG1	3.18	30
ATG34	3.15	30
ATG1	3.13	30
IDP3	3.13	30
CLD1	3.12	30
MNC1	3.11	30

PEX11	3.10	30
SPS19	3.08	30
LEE1	3.07	10.18
SDH6	3.06	30
ALD2	3.04	30
HFD1	3.04	30
YPT53	3.02	30
ZTA1	3.01	30
UME6	2.99	14.86
ATG9	2.99	30
HHF1	2.99	30
UGX2	2.99	30
PCK1	2.97	30
DBP1	2.96	30
OM14	2.95	30
PAU10	2.94	0.67
ISF1	2.94	30
TRS31	2.93	30
VHS2	2.92	30
ISU1	2.91	30
RIM101	2.91	30
RRT1	2.91	30
SDH2	2.91	30
CAT8	2.90	30
CIT3	2.90	30
SFC1	2.89	9.94
IZH4	2.88	16.77
CCC1	2.88	30
IRC13	2.87	20.91
MHO1	2.87	30
MIX17	2.87	30
PMP3	2.87	30
RSB1	2.87	30
ATO2	2.86	30
BLM10	2.86	30
CUP2	2.86	30
LCL1	2.86	30
UBX5	2.86	30

DPA10	2.85	30
ALT2	2.84	30
ERV1	2.84	30
SPG3	2.84	30
DIA1	2.83	30
ENA5	2.82	30
MOH1	2.80	30
RAD55	2.79	20.03
RPT6	2.79	30
PRM3	2.76	14.71
FKS3	2.76	30
GEX1	2.75	0.00
DIN7	2.75	14.22
PEX9	2.75	15.31
GIS1	2.75	30
SPO20	2.75	30
CMC4	2.74	30
PIL1	2.74	30
USV1	2.74	30
BYE1	2.73	30
DOC1	2.73	30
PLB2	2.73	30
YCP4	2.73	30
COB	2.72	30
MEP2	2.71	15.40
UMP1	2.71	30
EAR1	2.70	30
NDL1	2.70	30
NGL3	2.70	30
CTF19	2.69	30
MGR3	2.68	30
TMA10	2.68	30
GTO3	2.67	7.09
VPS27	2.67	30
STB3	2.66	19.32
PEX28	2.66	30
SLS1	2.65	2.87
GPG1	2.65	30

SWI1	2.64	30
RKM5	2.63	13.98
RGS2	2.63	15.96
SKS1	2.63	30
HYR1	2.62	30
APE3	2.61	30
HUB1	2.61	30
KHA1	2.61	30
GLO1	2.60	30
SNA3	2.60	30
CIP1	2.59	9.23
RCF3	2.59	11.14
PFK26	2.58	30
SOK2	2.57	9.78
NIS1	2.57	30
PBI2	2.57	30
ATG36	2.56	14.53
MPO1	2.56	30
PEX18	2.54	14.68
ATG8	2.54	30
DDI1	2.54	30
HXT4	2.54	30
NFI1	2.54	30
PHO92	2.53	14.07
ARA1	2.53	30
CRF1	2.53	30
FUB1	2.52	30
RCN2	2.52	30
SUR1	2.52	30
AST2	2.51	11.48
BI4	2.51	30
DIP5	2.50	30
PET20	2.50	30
PHO4	2.50	30
ARA2	2.49	15.95
AAC1	2.49	30
FAA4	2.49	30
HHO1	2.49	30

SAF1	2.48	18.10
MDH3	2.48	30
NBP35	2.48	30
PEX22	2.47	17.43
ENA2	2.47	30
NTH2	2.47	30
RPM1	2.47	30
SUE1	2.47	30
UBC8	2.47	30
MEI5	2.46	0.00
GIP2	2.46	30
HUA1	2.46	30
MPT5	2.45	13.99
TIM13	2.45	17.35
SNF11	2.45	18.80
YAK1	2.45	30
SNC2	2.44	30
ZWF1	2.44	30
HMLALPHA1	2.43	0.00
EMT2	2.43	1.38
DAT1	2.43	30
MIN8	2.43	30
TPH3	2.43	30
EMT3	2.42	0.08
AMS1	2.42	30
FDH1	2.41	17.41
ATG39	2.41	30
ENA1	2.41	30
GAP1	2.41	30
OPY1	2.40	11.97
PEX30	2.40	30
CPS1	2.39	30
FMP40	2.39	30
KIC1	2.39	30
MEF2	2.39	30
OTU1	2.39	30
PPQ1	2.39	30
RDL1	2.39	30

GYP7	2.38	30
LSP1	2.38	30
RRT8	2.38	30
BSC2	2.37	30
NGR1	2.36	8.27
RIB1	2.36	30
LGE1	2.35	30
PSP1	2.35	30
CNL1	2.34	30
AIM19	2.33	30
BI2	2.33	30
GPT2	2.33	30
MPM1	2.33	30
NQM1	2.33	30
RRG9	2.33	30
FZO1	2.32	7.49
WTM1	2.32	30
YAP1	2.32	30
YET2	2.32	30
CSS3	2.31	30
GET3	2.31	30
ILT1	2.31	30
PRB1	2.30	30
FMP49	2.29	3.93
45436	2.29	10.77
LPX1	2.29	30
TPK1	2.29	30
CDC26	2.28	6.51
HTA2	2.28	17.30
CAR2	2.28	30
MUB1	2.28	30
RPT2	2.28	30
YOR1	2.28	30
FLO11	2.27	2.94
HOT13	2.27	5.08
CDC53	2.27	30
SCS7	2.27	30
URA10	2.27	30

YGP1	2.27	30
YPF1	2.27	30
MIN10	2.26	4.53
CDC27	2.26	7.50
ATG4	2.26	16.91
COX7	2.26	30
EDC2	2.26	30
SSH4	2.26	30
GIP1	2.25	8.25
MIN6	2.25	15.44
NPR1	2.25	30
ROY1	2.25	30
RPN8	2.25	30
DEF1	2.24	30
MCO10	2.24	30
MPD2	2.24	30
RAD4	2.24	30
REH1	2.24	30
TOD6	2.23	9.71
MVP1	2.23	17.99
ICT1	2.23	30
RCR1	2.23	30
SNX4	2.23	30
GAT2	2.22	10.02
CCL1	2.22	30
CCS1	2.22	30
GLC8	2.22	30
RRD1	2.22	30
GPN2	2.21	30
SEM1	2.21	30
SPO24	2.21	30
TFG2	2.21	30
SNC1	2.20	6.74
TSC10	2.20	7.38
DAN1	2.20	8.17
GIS3	2.20	12.97
YAT2	2.20	16.76
RFA3	2.20	18.09

MPC1	2.20	30
XBP1	2.19	8.17
TFC3	2.19	9.86
COX1	2.19	30
HRP1	2.19	30
MSC3	2.19	30
BDF2	2.18	30
BI3	2.18	30
KNS1	2.18	30
PEX3	2.18	30
STE24	2.18	30
STF1	2.18	30
SUM1	2.18	30
SUT2	2.17	11.01
RRN3	2.17	30
HVG1	2.16	3.79
TES1	2.16	3.81
LUG1	2.16	7.90
RSA1	2.16	11.18
AI5_ALPHA	2.16	30
SSM4	2.16	30
CTR3	2.15	4.98
GPI10	2.15	11.31
PBA1	2.15	13.52
BUD23	2.15	30
PEP4	2.15	30
ATH1	2.14	30
BXI1	2.14	30
IRC20	2.14	30
HAL9	2.13	7.31
CAF40	2.13	8.36
ICL2	2.13	14.60
AI4	2.13	30
NPC2	2.13	30
FMS1	2.12	30
UGA2	2.11	13.09
ATG11	2.11	15.42
LAP3	2.11	30

VHS3	2.11	30
ELP6	2.10	2.87
CTK3	2.10	6.18
DDP1	2.10	30
GAD1	2.10	30
NBL1	2.10	30
NHP6B	2.10	30
SKP1	2.10	30
ATG14	2.09	1.70
MRX8	2.09	7.51
YTA7	2.09	30
HUR1	2.08	30
CBF1	2.07	5.84
CDC23	2.07	9.19
FMP46	2.07	30
VPS55	2.07	30
GMC2	2.06	1.97
PRP18	2.06	8.97
PMC1	2.06	30
INP54	2.05	6.06
AI3	2.05	30
YME2	2.05	30
WAR1	2.04	8.14
SIP2	2.04	15.81
AI2	2.04	30
AVT3	2.03	30
GLO4	2.03	30
PEX15	2.02	16.91
HUL5	2.02	30
RPS30B	2.02	30
IMT4	2.01	0.72
SUF4	2.01	0.72
ATG5	2.01	16.30
CSR2	2.01	16.90
PDC6	2.00	4.77
AHC1	2.00	5.49
SDH8	2.00	13.67
HEM2	2.00	16.83

CSC1	2.00	30
MPE1	2.00	30
NAB3	2.00	30
PRC1	2.00	30
PTR2	2.00	30
SSL2	2.00	30
TRX3	2.00	30
TVP18	2.00	30
GNP1	1.99	10.44
CAT2	1.99	12.28
HRT1	1.99	30
XYL2	1.98	3.54
PRY2	1.98	13.71
COX3	1.98	30
GDH2	1.98	30
TMC1	1.98	30
UBP6	1.98	30
HYM1	1.97	6.17
COX16	1.97	15.15
PRE5	1.97	30
RAD51	1.97	30
RTG1	1.97	30
TFS1	1.97	30
COS9	1.96	7.54
GND2	1.96	30
HAA1	1.96	30
KGD2	1.96	30
TRS23	1.96	30
TBS1	1.95	7.82
ULA1	1.95	11.61
GFD1	1.95	30
RHO5	1.95	30
PTK1	1.94	4.50
KEI1	1.94	5.30
GOS1	1.94	11.05
MET28	1.94	13.02
FAA1	1.94	30
IMP2'	1.94	30

PHB2	1.94	30
TDA11	1.93	5.67
SKN7	1.93	6.51
PRP31	1.93	7.62
BPT1	1.93	30
RPS27B	1.93	30
MCO32	1.92	4.13
CST6	1.92	10.84
NCB2	1.92	13.85
VPS29	1.92	14.54
MGA2	1.92	30
RPS27A	1.92	30
SND1	1.92	30
RNR3	1.91	2.32
UBX4	1.91	4.42
21S_RRNA	1.91	30
MET30	1.91	30
PAN3	1.90	3.65
DAS1	1.90	8.03
CSS2	1.90	10.64
MRPL28	1.90	13.70
PCP1	1.90	15.00
KRE1	1.90	30
SHP1	1.90	30
WSC2	1.89	6.09
PDR1	1.89	15.92
CDD1	1.89	16.18
RTC6	1.89	30
STE11	1.89	30
UBC13	1.89	30
SEN34	1.88	3.01
RBL2	1.88	4.87
VID24	1.88	5.86
LDO16	1.88	7.63
RAP1	1.88	9.26
SLM3	1.88	13.50
SUR2	1.88	30
ICL1	1.87	9.68

TOP3	1.87	14.09
UGA1	1.87	15.79
FIS1	1.87	30
ORM2	1.87	30
PET100	1.87	30
PGA2	1.87	30
RTR2	1.86	5.40
COA4	1.86	6.40
RFA1	1.86	12.05
MIN7	1.86	30
JHD1	1.85	6.60
PEX35	1.85	7.18
SDP1	1.85	9.57
TPS2	1.85	30
PAU22	1.84	0.00
SUP16	1.84	0.71
RPI1	1.84	5.09
PFU1	1.84	11.33
ATG19	1.84	11.37
DBP2	1.84	30
PMR1	1.84	30
RVS161	1.84	30
NIT2	1.83	5.66
MAF1	1.83	10.33
AFG3	1.83	30
COX2	1.83	30
HTA1	1.83	30
TIR2	1.81	1.77
ISA1	1.81	30
SPF1	1.81	30
AGP1	1.80	13.76
AI1	1.80	30
FMP52	1.80	30
RFA2	1.79	30
SNQ2	1.79	30
ECM10	1.78	4.29
SHU2	1.78	4.77
TAD2	1.78	5.64

HXT11	1.78	6.84
CDC8	1.78	7.11
BRE4	1.78	11.80
PRE10	1.78	30
NAS6	1.77	11.88
RNA14	1.77	11.94
MNN5	1.77	30
OAZ1	1.77	30
PRE3	1.77	30
MET8	1.76	1.52
GLG2	1.76	4.49
COX23	1.76	7.31
HIT1	1.76	7.31
BCP1	1.76	10.15
FZF1	1.76	10.73
CAB4	1.76	11.63
MRPL16	1.76	12.65
RPT4	1.76	30
MMO1	1.75	1.35
SNA4	1.75	3.54
MSG5	1.75	4.13
GPA2	1.75	13.75
LPL1	1.75	30
RAD54	1.75	30
CMR3	1.74	1.40
PIG2	1.74	3.06
CAF120	1.74	4.31
PGD1	1.74	4.74
HXT9	1.74	6.08
FAA2	1.74	6.23
EMP46	1.74	6.71
TRR2	1.74	9.38
COQ4	1.74	12.33
CDC37	1.74	30
RPT1	1.74	30
FHN1	1.73	3.42
HMS1	1.73	5.99
MKK1	1.73	12.53

PDH1	1.73	14.92
APJ1	1.73	30
NOP16	1.73	30
SDD3	1.73	30
SSD1	1.73	30
PDS1	1.72	5.00
SAN1	1.72	8.85
FYV6	1.72	9.61
MTR2	1.72	30
PRY1	1.71	1.68
REC104	1.71	5.64
VPS72	1.71	5.65
PAH1	1.71	7.38
PCT1	1.71	30
RPN3	1.71	30
MDG1	1.70	12.88
CCT5	1.70	30
ECM15	1.70	30
YPT11	1.69	2.33
SNF1	1.69	4.92
RAD14	1.69	5.32
TGL4	1.69	9.07
RAD16	1.69	9.29
ISY1	1.69	14.74
FRQ1	1.69	30
ATG31	1.68	2.68
POG1	1.68	4.60
INA17	1.68	9.47
GCD1	1.68	13.06
PRE4	1.68	30
SSU72	1.67	2.40
CDC123	1.67	3.54
SWC5	1.67	4.60
BSC4	1.67	4.74
WHI5	1.67	6.02
HTL1	1.67	8.16
DSC2	1.67	13.75
DPL1	1.67	30

LDB7	1.67	30
NCE102	1.67	30
PGA3	1.67	30
YSA1	1.66	6.50
CWC24	1.66	9.68
CCT3	1.66	30
SOD1	1.66	30
UBI4	1.66	30
TEN1	1.65	6.16
MAG1	1.65	30
MDH1	1.65	30
PRE9	1.65	30
TIF5	1.65	30
SPO11	1.64	0.47
ASG7	1.64	1.78
FUN19	1.64	3.45
RRG1	1.64	3.49
NNF2	1.64	4.81
SPT10	1.64	4.87
DPB4	1.64	8.78
RPC31	1.64	9.07
ASK10	1.64	11.98
GIM4	1.64	13.79
NAR1	1.64	30
IME1	1.63	0.53
HOT1	1.63	1.99
MCH2	1.63	4.41
GRR1	1.63	7.99
SCH9	1.63	11.33
MRPL51	1.63	12.68
FET5	1.63	13.38
ESA1	1.63	14.85
COA3	1.63	30
CRP1	1.63	30
QCR6	1.63	30
SOD2	1.63	30
ATG33	1.62	2.46
SPC42	1.62	2.75

YRR1	1.62	3.56
RAD30	1.62	4.06
AQY1	1.62	4.55
MND2	1.62	4.73
EFM4	1.62	7.62
PLB3	1.62	8.50
PEX19	1.62	13.33
GLC3	1.62	30
ISR1	1.61	1.96
FIT3	1.61	2.48
PTI1	1.61	2.64
NTO1	1.61	3.49
ATG21	1.61	5.13
WTM2	1.61	6.86
MKK2	1.61	11.36
QCR10	1.61	30
TCP1	1.61	30
ECM12	1.60	0.95
TUL1	1.60	5.78
CCT8	1.60	30
UBA1	1.60	30
MRP10	1.59	12.47
HSV2	1.59	15.40
INH1	1.59	30
MIC26	1.59	30
RPT3	1.59	30
SND3	1.59	30
SYG1	1.59	30
YRA1	1.59	30
WHI3	1.58	2.46
SWF1	1.58	4.46
MDM34	1.58	5.28
PEX5	1.58	6.84
TGL3	1.58	7.04
RMD5	1.58	9.81
THI6	1.58	10.00
PUT2	1.58	10.82
TOA1	1.58	11.43

CBP4	1.58	15.10
HST1	1.58	30
RPN13	1.58	30
CHK1	1.57	3.14
FEX2	1.57	3.70
PXA2	1.57	4.13
SNG1	1.57	5.91
NNK1	1.57	6.13
VPS35	1.57	10.86
RUB1	1.57	15.22
CDC34	1.57	30
DSK2	1.57	30
PRE2	1.57	30
PRE7	1.57	30
UFD1	1.57	30
DPC25	1.56	4.52
PSH1	1.56	6.61
RNH70	1.56	15.10
DPI8	1.56	30
MDV1	1.55	4.33
UBX7	1.55	7.58
RTT102	1.55	8.86
MAP1	1.55	10.11
GRX2	1.55	14.21
OLI1	1.55	30
PUP2	1.55	30
FMP23	1.54	1.73
ECM27	1.54	4.06
CPT1	1.54	5.87
SRC1	1.54	7.75
ORM1	1.54	8.21
HSF1	1.54	8.36
FAP1	1.54	8.49
SLG1	1.54	30
YTP1	1.53	3.03
HEL1	1.53	3.35
RSF1	1.53	5.10
MCM1	1.53	5.26

MAK3	1.53	6.14
COQ21	1.53	6.48
SEC17	1.53	14.04
ACO1	1.53	30
SIC1	1.52	8.28
NCE101	1.52	10.06
GSP2	1.51	2.97
SDS24	1.51	3.45
CAB2	1.51	4.06
KEL1	1.51	4.21
BAG7	1.51	5.17
MATALPHA2	1.51	6.39
SHE10	1.51	9.10
QNS1	1.51	9.49
GRE2	1.51	13.34
GID8	1.51	30
CHM7	1.50	3.77
INO2	1.50	3.88
RCI50	1.50	4.32
NUM1	1.50	11.27
AIM41	1.50	11.55
ROX3	1.50	30
SBA1	1.50	30
VFA1	1.49	1.51
SFL1	1.49	2.83
TFB4	1.49	4.62
YFT2	1.49	6.82
VID27	1.49	7.31
FLC2	1.49	11.94
GLT1	1.49	12.95
CCT2	1.49	30
TMA17	1.49	30
SUS1	1.48	2.18
BEM1	1.48	3.69
ELP4	1.48	7.08
HTB1	1.48	8.82
YPR1	1.48	9.49
TOM70	1.48	9.87

PUT3	1.48	10.25
ZRT3	1.48	10.84
SSU1	1.48	13.75
RPN1	1.48	30
TOM7	1.48	30
UGA4	1.47	0.90
NCA3	1.47	2.53
AIM18	1.47	3.81
CKB2	1.47	9.56
RPN7	1.47	30
YKT6	1.47	30
PSO2	1.46	2.22
BIG1	1.46	2.23
ETP1	1.46	3.52
TRI1	1.46	3.52
ETR1	1.46	4.45
SGT1	1.46	5.57
SNA2	1.46	13.76
EMI1	1.45	2.43
OAF1	1.45	2.91
RRT6	1.45	3.09
SWM2	1.45	4.65
GSH1	1.45	5.76
SYS1	1.45	6.56
MCH1	1.45	8.23
RSC2	1.45	8.45
ULS1	1.45	9.08
IWR1	1.45	10.43
NYV1	1.45	11.93
GPD1	1.45	13.71
AIM46	1.45	14.32
SPT5	1.45	14.70
RPB4	1.45	30
SOL2	1.45	30
YSF3	1.44	2.45
PKH1	1.44	3.51
THP3	1.44	4.98
CIN5	1.44	6.58

YPS1	1.44	11.27
SUP17	1.43	0.31
QDR1	1.43	2.81
ARK1	1.43	2.87
HPA2	1.43	2.94
VPS73	1.43	2.96
RTS2	1.43	3.30
RNQ1	1.43	5.12
MET4	1.43	8.36
LSM3	1.43	9.12
MIA40	1.43	30
BSC1	1.42	1.86
SPR1	1.42	2.15
MUM2	1.42	2.53
MRM2	1.42	5.06
MIH1	1.42	6.51
SUB1	1.42	8.03
MRS6	1.42	13.66
SAP155	1.42	14.32
PYC1	1.42	30
YPT52	1.42	30
ABM1	1.41	2.09
RAS1	1.41	2.48
HSM3	1.41	2.56
SFT1	1.41	7.71
RPS30A	1.41	30
TMH11	1.41	30
ADY4	1.40	3.07
TRM12	1.40	5.26
CFD1	1.40	5.50
RTT103	1.40	6.61
RHO1	1.40	30
IDP2	1.39	2.08
CDC16	1.39	3.05
HTZ1	1.39	5.08
MLO1	1.39	5.73
ESL2	1.39	6.98
SRP54	1.39	14.17

MRP8	1.39	30
AIM9	1.38	6.32
RER1	1.38	6.43
ATG20	1.38	6.46
NUP2	1.38	8.09
GLO2	1.38	15.70
GLN1	1.38	30
VPS21	1.38	30
HFL1	1.37	3.99
VID30	1.37	4.91
PAP2	1.37	5.37
LSM7	1.37	5.86
ECM21	1.37	7.32
FYV10	1.37	7.94
SGT2	1.37	30
RRT7	1.36	0.43
YOX1	1.36	1.39
TMA23	1.36	1.74
AZF1	1.36	2.76
PCI8	1.36	3.51
CDH1	1.36	3.69
PTC1	1.36	6.19
RSM22	1.36	6.57
TFA2	1.36	8.19
PUP1	1.36	11.93
YTA12	1.36	12.10
GUT1	1.36	15.22
GRX4	1.35	4.15
MRK1	1.35	4.46
NHP6A	1.35	5.92
GRX3	1.35	6.85
VMA10	1.35	9.61
SCS2	1.35	9.94
FMP10	1.35	11.27
TIM21	1.35	11.49
RPN11	1.35	30
ARP10	1.34	0.63
SPP381	1.34	1.85

RAM1	1.34	2.23
SLF1	1.34	3.41
CAP1	1.34	6.10
BSD2	1.34	8.57
GTR1	1.34	9.02
BDF1	1.34	12.58
KTR1	1.34	30
BOP3	1.33	1.07
ASG1	1.33	2.38
UME1	1.33	3.82
BRE1	1.33	5.93
UBP13	1.33	7.30
RDS3	1.33	8.30
MIX23	1.33	9.09
NPA3	1.33	10.40
AI5_BETA	1.33	30
ARF2	1.33	30
MIT1	1.32	0.85
UBC9	1.32	4.59
AFT2	1.32	6.06
ATG26	1.32	6.80
MRPL33	1.32	8.62
RPL29	1.32	9.12
NNR1	1.32	11.99
TIF35	1.32	30
MSS11	1.31	0.81
CRZ1	1.31	1.98
ALP1	1.31	3.12
NUR1	1.31	3.78
RRP46	1.31	6.08
GAC1	1.31	10.30
SKN1	1.31	11.08
MRPS18	1.31	11.49
CHZ1	1.31	13.28
CCT6	1.31	30
RNR2	1.31	30
SCL1	1.31	30
YPT1	1.31	30

HPC2	1.30	0.79
REF2	1.30	2.58
PDR8	1.30	2.77
JID1	1.30	2.84
MET13	1.30	3.04
ALO1	1.30	4.80
PKH2	1.30	5.86
ASK1	1.30	6.45
SPN1	1.30	10.93
CUZ1	1.30	15.40
PFK1	1.30	30
SAW1	1.29	1.79
MCO76	1.29	1.92
RGD1	1.29	2.02
HNT3	1.29	2.08
RIT1	1.29	2.23
ROG1	1.29	2.41
YIM1	1.29	5.13
TMS1	1.29	5.29
PTH4	1.28	1.05
GON7	1.28	4.08
MXR1	1.28	4.46
NPL4	1.28	4.66
NOG2	1.28	10.77
IBA57	1.27	1.59
DID4	1.27	5.87
YNG2	1.27	5.93
UTR1	1.27	8.99
LSB3	1.27	10.12
NFS1	1.27	30
SRN2	1.26	1.75
CMD1	1.26	5.90
FPS1	1.26	12.67
RPN6	1.26	30
OAR1	1.25	2.22
VID28	1.25	2.68
MNN14	1.25	3.16
ECM29	1.25	3.89

NAM9	1.25	4.43
ASR1	1.25	4.47
GAL83	1.25	5.16
SNF3	1.25	6.05
KTR4	1.25	13.13
OPY2	1.25	30
MGR1	1.24	0.99
DMA2	1.24	1.37
VNX1	1.24	1.52
MED7	1.24	2.03
MOT2	1.24	2.20
TAF13	1.24	3.35
YIP3	1.24	4.67
TAF12	1.24	8.60
PBP4	1.24	9.43
PBN1	1.24	9.49
SUA7	1.24	13.84
EFT1	1.24	30
MRPL39	1.24	30
NUP60	1.23	1.44
MED2	1.23	2.78
ALG13	1.23	3.44
MGM1	1.23	3.70
TRM112	1.23	3.89
MAM3	1.23	5.38
UBC6	1.23	5.41
CTR2	1.23	7.71
CMI7	1.23	12.50
PEP12	1.23	15.22
CWC21	1.22	1.46
ASI3	1.22	2.37
FEX1	1.22	2.62
KEL2	1.22	2.62
VHS1	1.22	2.89
TAF1	1.22	6.73
MHF1	1.22	7.14
CMK2	1.22	12.41
OLE1	1.22	30

STB1	1.21	0.72
SVL3	1.21	1.62
GPI19	1.21	1.96
MPS3	1.21	2.27
SEG1	1.21	4.86
MRPL31	1.21	6.81
UCC1	1.21	9.53
COX8	1.21	10.99
SSA3	1.21	30
ZAP1	1.20	0.50
ATG12	1.20	1.50
TIP20	1.20	1.87
VPS3	1.20	1.94
IRA1	1.20	2.26
PSF2	1.20	3.29
MRX9	1.20	3.32
VPS24	1.20	4.33
TAF14	1.20	4.41
PIB1	1.20	4.54
MTC1	1.20	5.29
RCI37	1.20	6.27
SPT16	1.20	6.42
CSG2	1.20	8.87
STP4	1.19	1.79
MCT1	1.19	2.01
VLD1	1.19	2.05
SNU114	1.19	2.33
MRP2	1.19	2.57
DUG3	1.19	3.23
NHX1	1.19	3.47
RRD2	1.19	3.47
CDC36	1.19	4.66
LIN1	1.19	5.75
MEH1	1.19	6.03
VTI1	1.19	6.14
POB3	1.19	7.37
MRPS16	1.19	7.80
RTK1	1.19	10.38

HAP2	1.18	1.20
SPP41	1.18	1.44
YHC3	1.18	1.78
GOT1	1.18	2.04
IBD2	1.18	2.38
MHP1	1.18	3.88
SOH1	1.18	3.90
INM2	1.18	5.15
ARG80	1.18	5.36
VPS70	1.18	5.64
CAF20	1.18	8.33
OPI1	1.18	8.72
HIR2	1.17	0.87
DCN1	1.17	1.35
SMK1	1.17	1.46
MGA1	1.17	1.57
STE7	1.17	1.89
ALG14	1.17	1.95
PIB2	1.17	2.04
HEM1	1.17	2.81
PCL7	1.17	4.07
DJP1	1.17	6.34
GTS1	1.17	7.78
ASN2	1.17	30
ATP12	1.16	1.89
SHR5	1.16	2.26
SCD5	1.16	2.28
PCL8	1.16	2.60
PHO2	1.16	2.67
PCL10	1.16	4.16
SDH5	1.16	5.41
TRE2	1.16	5.57
PDR12	1.16	7.45
MNR2	1.16	10.61
SNX41	1.15	0.68
RAD50	1.15	2.16
HMLALPHA2	1.15	3.02
PDR15	1.15	3.61

LEA1	1.15	3.79
INO4	1.15	4.48
PAR32	1.15	5.42
CPR2	1.15	8.83
MIM1	1.15	8.94
NPP1	1.15	11.04
SMX3	1.15	11.48
DLD3	1.15	30
SEN54	1.14	1.70
PET130	1.14	1.74
TPC1	1.14	1.85
ASE1	1.14	2.47
RSN1	1.14	3.04
YPT32	1.14	3.52
YFH1	1.14	3.96
RAD1	1.14	4.04
TAF4	1.14	4.17
MSP1	1.14	5.27
VPS62	1.14	5.44
SPT6	1.14	5.85
ERC1	1.14	5.94
GPM2	1.14	7.06
NVJ1	1.14	9.36
FBP26	1.14	11.15
PFA3	1.13	1.58
TMH18	1.13	2.81
EAF3	1.13	3.50
IOC4	1.13	3.80
YHC1	1.13	4.90
RFC3	1.13	5.28
NIF3	1.13	5.35
CCT7	1.13	5.79
FTH1	1.13	6.97
FPK1	1.13	7.42
HSP31	1.13	9.55
DED1	1.13	30
ECM11	1.12	0.08
ECM2	1.12	2.53

AGP2	1.12	2.69
PAN6	1.12	3.03
SWT21	1.12	3.26
ALD6	1.12	4.80
IPT1	1.12	5.30
MAG2	1.12	5.38
SIP4	1.11	1.96
MNE1	1.11	2.05
UBX3	1.11	2.36
ERR3	1.11	2.72
AOS1	1.11	2.83
YAF9	1.11	3.29
CTK1	1.11	4.36
SNF7	1.11	6.91
PSG1	1.11	7.73
RAD23	1.11	9.34
TIM17	1.11	15.70
BAP2	1.11	30
CWC2	1.10	1.61
ELA1	1.10	1.75
RAD5	1.10	2.99
RAV2	1.10	3.00
SWR1	1.10	3.78
ITT1	1.10	4.04
OCA1	1.10	5.00
IGO2	1.10	8.54
GPX2	1.09	0.71
AME1	1.09	0.82
PDR3	1.09	1.08
ULP1	1.09	2.82
RGT2	1.09	3.27
PET191	1.09	3.47
RQC1	1.09	3.59
YIF1	1.09	4.70
PRE1	1.09	11.20
COX13	1.09	30
PRX1	1.09	30
MSI1	1.08	0.85

NRG2	1.08	0.92
VAR1	1.08	1.07
LEU3	1.08	1.57
MRX10	1.08	1.67
SGE1	1.08	2.09
ATP25	1.08	2.32
YUR1	1.08	2.98
ATG15	1.08	3.26
APP1	1.08	3.94
NST1	1.08	4.79
SEC4	1.08	8.40
GAB1	1.07	0.98
URN1	1.07	1.46
TFB3	1.07	1.76
PEX31	1.07	1.79
SPP1	1.07	2.45
IRC24	1.07	3.07
FYV8	1.07	3.45
NCR1	1.07	5.53
RSM19	1.07	11.56
MSA1	1.06	1.11
ARL3	1.06	1.89
GPI8	1.06	2.80
DUF1	1.06	3.27
GCR2	1.06	3.50
MSN5	1.06	6.75
PFK2	1.06	30
PLN1	1.06	30
QCR8	1.06	30
ATG16	1.05	1.21
IRA2	1.05	4.75
RPL14B	1.05	4.93
ADR1	1.05	5.76
MET12	1.05	9.34
TOA2	1.05	9.54
CCT4	1.05	10.61
IGO1	1.05	30
MER1	1.04	0.44

UGO1	1.04	0.56
MAM33	1.04	2.99
IST1	1.04	3.39
DNM1	1.04	4.08
RUP1	1.04	4.22
TOM22	1.04	7.44
RPN5	1.04	14.38
PGI1	1.04	30
PNT1	1.03	0.48
FRE4	1.03	0.88
DAL80	1.03	1.10
USE1	1.03	2.45
UFE1	1.03	2.73
RSC4	1.03	2.88
HRT3	1.03	3.00
DIC1	1.03	3.12
IMP1	1.03	3.21
MRX14	1.03	3.68
RPO31	1.03	3.76
YPT7	1.03	4.03
PAN2	1.03	4.65
PAU21	1.02	0.00
NRM1	1.02	0.39
TFC1	1.02	0.87
IAI11	1.02	1.32
YAP6	1.02	1.44
CIN4	1.02	1.54
PEX29	1.02	1.57
STP1	1.02	1.97
IES2	1.02	2.64
MSC6	1.02	2.83
LRE1	1.02	2.88
CDC40	1.02	3.44
APE4	1.02	3.60
LSM2	1.02	3.92
MRPL10	1.02	4.24
LYS20	1.02	4.94
UBR1	1.02	5.79

HXT2	1.02	5.89
RPN12	1.02	6.11
SUF10	1.01	0.25
MSC2	1.01	1.14
UBS1	1.01	1.47
RMD1	1.01	1.60
TOS2	1.01	1.79
ATG40	1.01	2.25
YAE1	1.01	2.68
COX11	1.01	2.87
LIH1	1.01	2.99
BMH2	1.01	5.34
HNT1	1.01	8.43
RPT5	1.01	10.61
PCC1	1.00	1.57
TFC7	1.00	1.86
MTC6	1.00	2.71
ERF2	1.00	2.87
RAD52	1.00	3.19
MOG1	1.00	3.43
CPR4	1.00	5.33
RNR4	1.00	8.71
FBA1	1.00	30

a) -Log10 (p-value) greater than 30 is indicated as 30.

Table S8. YPH499/Co58 gene with decreased expression level due to mutation.

Gene or locus tag	Log2 (fold change)	Log10 (p-value)[a)]
RGI1	-8.13	30
CUP1-1	-7.56	30
CUP1-2	-7.41	30
CIR1	-6.46	30
HXT3	-6.44	30
HSP30	-5.96	30
SOP4	-5.84	30
DCD1	-5.80	30
MCM16	-5.70	30
PDC1	-5.51	30
CDA1	-5.48	30
ATX2	-5.42	30
CDC3	-5.35	30
HSP150	-5.31	30
CLB4	-5.25	30
DSF1	-5.13	30
CDC10	-5.10	30
YPS6	-5.06	30
UBP11	-5.05	30
REE1	-4.98	30
MRX15	-4.94	30
HXK2	-4.92	30
KIN3	-4.88	14.91
IRC7	-4.88	30
MVD1	-4.88	30
ULI1	-4.87	30
MRPL32	-4.86	30
SAE3	-4.83	12.74
ALT1	-4.79	30
RDS1	-4.79	30
NUC1	-4.77	30
BFR1	-4.72	30
RKM2	-4.67	30
THI12	-4.65	30
FRM2	-4.64	30
CYS3	-4.61	30

SSA4	-4.60	30
RAX2	-4.59	30
AIM17	-4.55	30
PUS4	-4.55	30
ALK1	-4.53	11.80
HXT13	-4.53	30
EXG1	-4.52	30
MAN2	-4.48	30
HXT14	-4.47	8.26
SUR7	-4.44	30
TKL1	-4.42	30
COS5	-4.41	30
CYK3	-4.34	30
SUC2	-4.33	30
NCW1	-4.30	30
NTG1	-4.25	30
RAI1	-4.24	30
XKS1	-4.23	30
RME1	-4.20	30
MIC60	-4.18	30
MYO4	-4.17	30
HOM3	-4.16	30
OPI10	-4.14	30
KAR1	-4.13	14.89
CYB5	-4.11	30
LYS9	-4.05	30
PAN5	-4.02	30
THI5	-4.01	7.84
YVC1	-4.00	14.90
FRS1	-4.00	30
ARO7	-3.98	30
TPS3	-3.98	30
PRO1	-3.97	30
PNP1	-3.93	30
ECM31	-3.91	9.69
BNA1	-3.89	30
COS3	-3.88	30
CRN1	-3.88	30

EGO4	-3.87	30
SAM4	-3.87	30
SPO74	-3.86	30
FKS1	-3.85	30
AAD3	-3.84	14.67
YIP5	-3.84	30
NCP1	-3.83	30
NUP188	-3.83	30
OYE2	-3.83	30
TWF1	-3.83	30
COQ2	-3.82	30
RDT1	-3.82	30
FAR1	-3.81	30
PFS1	-3.80	4.35
AGC1	-3.80	30
COS2	-3.78	30
ARO8	-3.76	30
KEL3	-3.76	30
PAU18	-3.75	8.64
MST1	-3.71	10.32
BNA3	-3.71	30
ENO2	-3.70	30
SAM1	-3.70	30
PMA1	-3.69	30
RRT12	-3.67	10.79
KAE1	-3.66	30
CCA1	-3.65	30
PRO3	-3.65	30
VTH2	-3.65	30
ABP1	-3.63	30
ARI1	-3.63	30
CLN1	-3.62	9.32
MIS1	-3.61	30
CIS3	-3.60	30
MKC7	-3.60	30
ERG4	-3.59	30
MLO50	-3.59	30
SWI5	-3.58	5.44

VTH1	-3.58	30
EHT1	-3.57	30
HEM25	-3.57	30
MAL32	-3.57	30
NPY1	-3.57	30
SMC2	-3.57	30
CLP1	-3.56	10.58
URA6	-3.55	30
PHO13	-3.54	30
AXL1	-3.53	30
YHB1	-3.53	30
YMD8	-3.53	30
ADA2	-3.52	30
SRL1	-3.51	14.70
YHM2	-3.51	30
SKG6	-3.50	7.72
FIT2	-3.50	14.87
YPK2	-3.49	30
SRM1	-3.48	30
TIP1	-3.45	30
ASN1	-3.44	30
PST1	-3.44	30
SPO13	-3.43	4.75
SIL1	-3.43	30
DAL7	-3.40	30
GCV1	-3.40	30
GPM3	-3.40	30
HXK1	-3.39	30
PPZ1	-3.39	30
TOS4	-3.38	3.55
SCP160	-3.38	30
LDS1	-3.37	5.19
RTC4	-3.37	30
TUB3	-3.36	30
SAP4	-3.35	11.30
ERG26	-3.35	30
PHR1	-3.35	30
RBH2	-3.35	30

CHS3	-3.34	30
RNH203	-3.34	30
TRM9	-3.34	30
ADE1	-3.33	30
PBI1	-3.32	9.49
MAL12	-3.32	30
HTS1	-3.31	30
LYS4	-3.30	30
SKT5	-3.30	30
HIS2	-3.29	30
MRPL25	-3.29	30
RGA1	-3.29	30
SUN4	-3.29	30
SER2	-3.28	30
TEM1	-3.27	14.15
AFR1	-3.27	30
SAC1	-3.27	30
YRF1-4	-3.26	30
POP1	-3.25	30
GSH2	-3.24	30
MSW1	-3.24	30
SPT14	-3.24	30
SHE3	-3.23	6.35
FIR1	-3.22	2.54
TAN1	-3.22	12.14
COQ3	-3.21	30
DOG2	-3.21	30
SCW4	-3.21	30
FRD1	-3.20	30
TPM1	-3.19	30
UTR2	-3.18	30
MCM5	-3.17	30
SHE1	-3.16	4.79
DUT1	-3.16	7.74
PAL1	-3.16	30
AAC3	-3.15	5.22
CTR1	-3.15	30
IMA2	-3.14	30

TOH1	-3.14	30
TRE1	-3.14	30
MDM10	-3.12	3.52
PML39	-3.10	14.58
GFD2	-3.10	16.02
RRG7	-3.10	30
SIA1	-3.10	30
BUD4	-3.09	13.21
RRI2	-3.09	14.64
CHA1	-3.09	30
BAR1	-3.08	6.96
KIP2	-3.08	8.52
MET10	-3.08	12.42
CPR5	-3.08	30
MEI4	-3.07	4.68
CLB2	-3.04	8.22
TIR3	-3.04	9.90
TFC8	-3.04	10.39
STE4	-3.04	30
AMN1	-3.03	30
PER1	-3.03	30
TTI1	-3.02	8.96
DAL1	-3.02	17.25
FPR4	-3.02	30
SKM1	-3.02	30
PAU6	-3.01	10.46
DED81	-3.01	30
PHO3	-3.00	5.44
ERG5	-3.00	30
STE5	-3.00	30
EPT1	-2.99	8.74
RSR1	-2.99	30
SHS1	-2.99	30
BBP1	-2.98	8.64
DIA2	-2.98	11.68
SMI1	-2.98	30
SPO77	-2.97	5.41
DSE4	-2.96	14.16

HIP1	-2.96	30
MRH1	-2.96	30
YRF1-8	-2.96	30
MAK10	-2.95	12.15
YRF1-6	-2.95	14.67
ERG27	-2.95	30
NAT5	-2.95	30
REX2	-2.95	30
ATP2	-2.94	30
SRX1	-2.94	30
FDO1	-2.93	7.16
POF1	-2.93	9.75
HGH1	-2.93	15.61
CBT1	-2.93	30
YRF1-1	-2.92	10.03
MSF1	-2.92	15.63
SES1	-2.92	30
YPS5	-2.91	10.20
SWI4	-2.91	10.50
FTR1	-2.91	30
SAH1	-2.90	30
CLN2	-2.89	8.30
ADH6	-2.89	30
FET4	-2.89	30
MRX1	-2.88	14.58
ARC40	-2.88	30
SRP101	-2.88	30
YRF1-3	-2.87	14.37
SPH1	-2.87	15.11
GCN1	-2.87	30
YPP1	-2.86	14.02
CLB6	-2.85	2.04
KTI12	-2.85	30
TPA1	-2.85	30
EGT2	-2.84	9.49
MYO1	-2.83	12.98
RPN14	-2.83	13.29
MME1	-2.83	16.15

GEA2	-2.83	30
GRS1	-2.83	30
HER2	-2.83	30
MRS3	-2.83	30
TRS120	-2.82	10.38
DOT5	-2.82	30
ELO3	-2.82	30
HXT16	-2.81	7.28
SLD3	-2.81	12.68
OSH6	-2.81	14.04
EMP24	-2.81	30
MGL2	-2.81	30
YAP7	-2.80	12.11
ERG6	-2.80	30
SER3	-2.80	30
STD1	-2.80	30
UTH1	-2.80	30
SCO2	-2.79	15.26
BUD7	-2.79	30
MYO2	-2.79	30
PIR3	-2.79	30
PMT2	-2.79	30
CTF13	-2.78	8.00
MIN9	-2.78	16.88
SEC61	-2.77	30
TRP2	-2.77	30
HAT2	-2.76	30
TGL1	-2.76	30
CDC5	-2.75	15.11
MDL2	-2.75	30
SRO9	-2.74	9.30
YRF1-2	-2.74	13.03
ALG3	-2.74	30
EMC1	-2.74	30
RPS17B	-2.74	30
LYS1	-2.73	30
RFC5	-2.72	7.85
URK1	-2.72	14.83

EMC5	-2.72	30
RCM1	-2.72	30
MDL1	-2.71	30
SPR3	-2.70	3.83
GAT4	-2.70	4.80
RCK1	-2.70	10.30
FRE7	-2.70	12.99
GAS3	-2.70	14.56
SMP1	-2.70	17.23
ARG7	-2.70	30
GSP1	-2.70	30
THI11	-2.69	2.75
SRB2	-2.69	17.23
ANS1	-2.69	30
ERD2	-2.69	30
MRPL7	-2.69	30
SEC11	-2.69	30
LDB17	-2.68	12.45
KAR5	-2.67	3.75
JLP2	-2.67	5.85
DOT1	-2.67	7.56
SCP1	-2.67	14.21
TRK1	-2.67	30
TAF11	-2.66	14.83
MYO5	-2.66	16.79
ADD37	-2.66	30
ARC19	-2.66	30
HDA1	-2.66	30
SNO2	-2.65	11.00
ACO2	-2.65	30
ERG11	-2.65	30
EDS1	-2.63	4.97
NMD4	-2.63	8.58
YRF1-7	-2.63	12.70
SWI6	-2.63	14.11
KDX1	-2.63	30
RPL18A	-2.62	30
BDH1	-2.61	30

PMU1	-2.61	30
SYM1	-2.61	30
RCE1	-2.60	3.12
PUL3	-2.60	7.65
RPC25	-2.60	10.97
SEC59	-2.60	13.11
PSD2	-2.60	16.85
YRF1-5	-2.60	17.00
ADE17	-2.60	30
SSO2	-2.60	30
YSP2	-2.58	30
CLB1	-2.57	5.88
WSC3	-2.57	7.35
MPH3	-2.57	10.82
SNZ3	-2.57	13.12
RBD2	-2.57	15.38
IFA38	-2.56	11.06
APM4	-2.56	30
GCV2	-2.56	30
MAS2	-2.56	30
URB2	-2.55	8.50
IQG1	-2.55	16.71
COS8	-2.55	30
TCD2	-2.55	30
SIP3	-2.54	4.83
FAL1	-2.54	8.27
ARP5	-2.54	11.87
GND1	-2.54	30
GUS1	-2.54	30
TAL1	-2.54	30
CDC14	-2.53	11.52
ERP2	-2.53	14.24
DOP1	-2.53	30
MCO14	-2.52	9.39
RUD3	-2.52	30
NNF1	-2.51	4.80
CUP9	-2.51	16.57
NUG1	-2.51	30

SCW11	-2.50	4.35
CSI2	-2.50	9.27
AXL2	-2.50	10.58
GUA1	-2.50	30
NSA1	-2.50	30
ADE6	-2.49	30
ALD5	-2.49	30
UTP10	-2.49	30
RSC3	-2.48	8.96
SSK22	-2.48	14.86
MMS21	-2.46	4.12
MPH2	-2.46	6.11
AIM10	-2.45	6.33
SOL3	-2.45	8.57
SNZ2	-2.44	9.07
VMR1	-2.44	14.59
ACS2	-2.44	30
SKG1	-2.44	30
FRE3	-2.43	7.19
VAC17	-2.43	12.64
SEC28	-2.43	30
TRM11	-2.42	12.44
HIS6	-2.42	13.30
NUP84	-2.42	15.98
ADH1	-2.41	30
GAR1	-2.41	30
SEC3	-2.41	30
SIM1	-2.41	30
SPC29	-2.40	6.61
CSE4	-2.40	17.52
UPS3	-2.40	17.62
ERG1	-2.40	30
HTC1	-2.40	30
PSA1	-2.40	30
IMD2	-2.39	9.88
MYO3	-2.39	11.53
SPE4	-2.39	12.22
LOA1	-2.39	13.68

KAR2	-2.39	30
THR1	-2.38	30
DUG1	-2.37	30
NUP145	-2.37	30
RPL2B	-2.37	30
RRT2	-2.36	3.39
RAD59	-2.36	7.69
HAM1	-2.36	12.96
APE1	-2.36	30
RIA1	-2.36	30
APC5	-2.35	5.95
AIM24	-2.35	7.15
BTN2	-2.35	30
UTP15	-2.35	30
CRH1	-2.34	30
ALK2	-2.33	5.75
GIN4	-2.33	8.83
BIK1	-2.33	11.15
BRP1	-2.33	11.53
ERG13	-2.33	30
CIN2	-2.32	6.09
RRF1	-2.31	7.89
ACP1	-2.31	14.46
DPH2	-2.31	30
KRS1	-2.31	30
ARP2	-2.30	30
ODC2	-2.30	30
POL5	-2.30	30
GIM5	-2.29	5.80
OSW1	-2.29	12.09
SET5	-2.29	14.99
DOA1	-2.29	30
ERG20	-2.29	30
SEC21	-2.29	30
TUB1	-2.29	30
URB1	-2.28	8.94
ELO1	-2.28	11.49
BUD14	-2.28	30

GDB1	-2.28	30
GRE3	-2.28	30
LSC2	-2.28	30
SUT1	-2.27	2.25
ORT1	-2.27	13.14
CRS1	-2.27	16.95
ACA1	-2.27	17.72
GOR1	-2.27	30
PWP1	-2.27	30
SIR2	-2.26	5.32
GRX8	-2.26	6.57
BUD3	-2.26	10.50
COP1	-2.26	30
BDS1	-2.25	10.61
EXG2	-2.25	30
GTT2	-2.25	30
PFY1	-2.25	30
ROM1	-2.25	30
URA2	-2.25	30
AZR1	-2.24	3.97
MRC1	-2.24	6.73
BCH1	-2.24	10.17
NOP58	-2.24	30
NKP2	-2.23	2.63
AFB1	-2.23	3.78
POL1	-2.23	4.83
GAL10	-2.22	2.68
NOP19	-2.22	6.12
KTR5	-2.22	14.29
HO	-2.21	3.36
SNO3	-2.21	8.40
OTU2	-2.21	13.69
AAP1	-2.21	30
TIF4632	-2.21	30
GNA1	-2.20	8.53
RPS18B	-2.20	30
DRS2	-2.19	6.83
OGG1	-2.19	7.20

SRD1	-2.19	14.43
MES1	-2.19	30
QCR2	-2.19	30
RPS22A	-2.19	30
SNF8	-2.19	30
RKM3	-2.18	4.28
TLG1	-2.18	6.19
ANB1	-2.18	10.51
ADH4	-2.18	30
CAR1	-2.18	30
RPL23B	-2.18	30
SMF1	-2.18	30
ALG1	-2.17	6.39
GGC1	-2.17	30
GPA1	-2.15	6.73
BSC5	-2.15	9.56
IMD4	-2.15	30
RPL22A	-2.15	30
YSC84	-2.15	30
MOB2	-2.14	3.92
SVS1	-2.14	4.49
ALG5	-2.14	7.09
LTV1	-2.14	16.11
EXP1	-2.14	17.95
ADE3	-2.14	30
RRT5	-2.13	2.81
SSA1	-2.13	30
SCW10	-2.12	15.43
LYS12	-2.12	30
PHD1	-2.12	30
HXT15	-2.11	7.17
RKM4	-2.11	10.52
SSA2	-2.11	30
RFC1	-2.10	8.93
NMA1	-2.09	5.43
TUM1	-2.09	12.29
CAF16	-2.09	30
EMI2	-2.09	30

LMO1	-2.08	3.24
VMA22	-2.08	5.02
CDC45	-2.08	5.37
CLA4	-2.08	6.52
QDR2	-2.08	9.08
CWH43	-2.08	30
FET3	-2.08	30
RPC82	-2.08	30
RPL9A	-2.08	30
SEC63	-2.08	30
SPT4	-2.08	30
PHS1	-2.07	7.71
EMP70	-2.07	10.04
RPL12A	-2.07	30
RPS28B	-2.07	30
SRL3	-2.07	30
PRR1	-2.06	8.01
AIM45	-2.06	30
PFK27	-2.06	30
TSA1	-2.06	30
KAR3	-2.05	6.18
PET8	-2.05	9.57
NUT1	-2.05	10.17
HRI1	-2.05	30
DIT2	-2.04	4.86
FDC1	-2.04	7.05
ARH1	-2.04	9.51
MON2	-2.04	10.70
PMT5	-2.04	12.43
NUP157	-2.04	17.74
GCV3	-2.04	30
MLH2	-2.03	3.58
AST1	-2.03	7.18
SEC65	-2.03	13.89
GUK1	-2.03	30
PRM5	-2.03	30
SPS22	-2.02	2.47
MCD1	-2.02	4.80

GEP3	-2.02	6.92
PRS4	-2.02	10.61
INP53	-2.02	15.70
MEP3	-2.02	30
RPL2A	-2.02	30
MRX4	-2.01	11.73
GFA1	-2.01	30
SFB3	-2.01	30
ROF1	-2.00	2.52
ARG2	-2.00	8.94
FCY1	-2.00	30
TYS1	-2.00	30
DSF2	-1.99	2.08
SKI2	-1.99	11.14
ACT1	-1.99	30
PCM1	-1.99	30
YRO2	-1.99	30
IML3	-1.98	3.73
ARP1	-1.98	4.54
ABZ2	-1.98	8.93
ENT1	-1.98	11.71
ATC1	-1.98	14.75
COM2	-1.98	30
APL3	-1.97	4.90
INA1	-1.97	9.69
TRS65	-1.97	11.43
MPD1	-1.97	11.46
MCH5	-1.97	30
MLC1	-1.97	30
RIM21	-1.97	30
SSB1	-1.97	30
TMA20	-1.97	30
TRR1	-1.97	30
YPD1	-1.97	30
SWE1	-1.96	11.33
SEC31	-1.96	16.09
YEF3	-1.96	30
GUP2	-1.95	2.79

MRX12	-1.95	6.80
RBS1	-1.95	7.47
EFR3	-1.95	10.33
COX5B	-1.95	30
RPP2A	-1.95	30
PGU1	-1.94	2.97
BOL3	-1.94	5.56
TRM3	-1.94	5.66
VPS75	-1.94	9.15
ALG6	-1.94	10.30
ARO1	-1.94	30
PHO91	-1.93	2.83
HIS7	-1.93	4.48
FRE1	-1.93	9.35
PER33	-1.93	11.61
GIM3	-1.93	30
SEC62	-1.93	30
CYC8	-1.92	3.79
IPL1	-1.92	4.15
AAD4	-1.91	6.01
CIK1	-1.91	6.05
ECM18	-1.91	8.32
ATP23	-1.91	8.59
VAN1	-1.91	13.07
SEC7	-1.91	13.50
MCD4	-1.91	13.58
SMF2	-1.91	30
AFT1	-1.90	4.29
SAL1	-1.90	5.44
RPS11B	-1.90	8.22
GDA1	-1.90	9.66
NEM1	-1.90	14.21
LTP1	-1.90	17.72
THI22	-1.89	7.01
OXA1	-1.89	10.51
BIO5	-1.89	11.47
ERG12	-1.89	12.35
SOR1	-1.89	13.39

KRE6	-1.89	30
CGI121	-1.88	4.36
IMA5	-1.88	4.71
AIM26	-1.88	4.92
AVT5	-1.88	5.31
SEC10	-1.88	6.87
PIF1	-1.88	7.59
ERG8	-1.88	9.38
STT3	-1.88	30
EGH1	-1.87	3.67
YCS4	-1.87	5.56
YMC1	-1.87	8.31
COG3	-1.86	4.38
DDI2	-1.86	4.66
MSY1	-1.86	8.84
KTR3	-1.86	9.14
RMD6	-1.85	3.23
IRC10	-1.85	4.61
ATM1	-1.85	5.12
RRT13	-1.85	6.58
DPP1	-1.85	11.38
RRP45	-1.85	11.98
EGD2	-1.85	30
HXT7	-1.85	30
VAM6	-1.84	6.01
SED4	-1.84	7.10
SEC27	-1.84	30
TPO2	-1.84	30
BUD9	-1.83	4.79
MMS1	-1.83	4.93
PRP5	-1.83	5.43
SNL1	-1.83	7.17
YSP3	-1.83	8.09
TCB2	-1.83	11.03
CTH1	-1.83	30
GCG1	-1.83	30
KTR2	-1.83	30
MPC2	-1.83	30

PAU23	-1.82	5.05
PRM8	-1.82	8.71
NAT1	-1.82	14.28
MET17	-1.82	14.36
ALR2	-1.82	16.08
GAS1	-1.82	30
SIN4	-1.81	7.50
PSD1	-1.81	8.45
VPH2	-1.80	4.22
OMS1	-1.80	10.79
INM1	-1.80	16.88
PUS1	-1.80	18.57
TDH2	-1.80	30
ZRT1	-1.80	30
ATG38	-1.79	3.52
TMN2	-1.79	3.74
COG4	-1.79	8.66
AHP1	-1.79	30
CHC1	-1.79	30
SNO1	-1.79	30
EST2	-1.78	2.14
GIC1	-1.78	3.47
COG8	-1.78	5.80
MID2	-1.78	6.02
MSH2	-1.78	8.27
RRB1	-1.78	30
WRS1	-1.78	30
BIT61	-1.77	2.35
DAL5	-1.77	5.06
PRM10	-1.77	5.48
SRV2	-1.77	7.41
VPS36	-1.77	9.55
RHO2	-1.77	10.35
BAP3	-1.77	11.66
TRA1	-1.77	13.58
MRP51	-1.77	18.47
AGX1	-1.77	30
PUF4	-1.76	7.79

SAP185	-1.76	12.26
CDC11	-1.76	12.37
CYC1	-1.76	30
SPC24	-1.75	2.97
PUS6	-1.75	3.51
UBP16	-1.75	4.73
AIM14	-1.75	8.04
PUS7	-1.75	12.79
YSC83	-1.75	15.69
ATP5	-1.75	30
TRM2	-1.74	8.03
DAD4	-1.74	15.10
ERG28	-1.74	16.84
MRPL11	-1.73	9.83
LRO1	-1.73	10.16
LSB1	-1.73	30
HOF1	-1.72	2.14
NIT1	-1.72	10.21
ERP4	-1.72	10.35
FCY21	-1.72	12.30
PTM1	-1.72	14.48
RPL11B	-1.72	30
HCM1	-1.71	2.34
MRS2	-1.71	2.46
PKR1	-1.71	10.40
EBP2	-1.71	15.96
HCS1	-1.70	3.66
PRP2	-1.70	4.69
PEX6	-1.70	5.53
IPK1	-1.70	6.50
MTQ1	-1.70	8.46
UTP13	-1.70	10.62
AVT1	-1.70	12.34
DPM1	-1.70	14.41
ISU2	-1.70	15.72
NOP56	-1.70	30
PIC2	-1.70	30
OKP1	-1.69	3.04

IRC8	-1.69	3.49
PGM1	-1.69	4.42
UTP8	-1.69	5.73
BNA6	-1.69	16.36
ADE8	-1.69	30
CHS7	-1.69	30
KRE9	-1.69	30
RPS8B	-1.69	30
PHO12	-1.68	4.57
STE6	-1.68	5.74
RPP1	-1.68	7.56
CYC2	-1.68	8.78
NUP170	-1.68	10.16
TAE1	-1.67	6.27
SOR2	-1.67	12.02
RAM2	-1.67	13.79
RPO26	-1.67	14.32
CUR1	-1.67	30
RPP0	-1.67	30
NDT80	-1.66	2.81
DBF2	-1.66	8.26
ARO4	-1.66	30
RPL15A	-1.66	30
REC8	-1.65	2.45
ASH1	-1.65	2.76
MCM21	-1.65	4.47
STU1	-1.65	4.61
NCL1	-1.65	10.63
MCM2	-1.65	10.98
POL32	-1.64	2.75
ARL1	-1.64	3.35
PEP5	-1.64	3.67
PSY3	-1.64	3.83
RCH1	-1.64	8.37
CKI1	-1.64	10.56
RFT1	-1.64	14.89
POM33	-1.64	16.15
PCL5	-1.64	30

FSH3	-1.63	5.17
RMD9	-1.63	6.32
TAF2	-1.63	6.92
ORC1	-1.63	8.57
TOS1	-1.63	12.91
THI80	-1.62	3.43
LOT6	-1.62	3.93
PRP42	-1.62	5.02
KIN4	-1.62	8.74
OAC1	-1.62	11.14
TEF4	-1.62	12.19
LIP5	-1.62	13.73
RPL34A	-1.62	30
PUS2	-1.61	4.10
KAP122	-1.61	5.12
SEC12	-1.61	6.57
PLP2	-1.61	7.85
NUS1	-1.61	11.50
DRS1	-1.61	14.00
ADO1	-1.61	30
HSP10	-1.61	30
VPS41	-1.60	4.43
GPI1	-1.60	5.30
STU2	-1.60	6.88
LNP1	-1.60	7.28
MPH1	-1.60	9.20
HXT1	-1.60	12.57
GYL1	-1.59	2.35
HED1	-1.59	3.75
YVH1	-1.59	4.37
PEP3	-1.59	4.90
NUP85	-1.59	8.25
PIN2	-1.59	10.36
EMW1	-1.59	11.23
NMT1	-1.59	12.19
SEC23	-1.59	17.30
MRT4	-1.59	30
CMR2	-1.58	3.32

CTF18	-1.58	3.37
HIM1	-1.58	4.75
DAS2	-1.58	4.76
MMM1	-1.58	5.78
PIR1	-1.58	14.05
GDE1	-1.58	14.85
ERG29	-1.57	3.23
DBP6	-1.57	4.02
PES4	-1.57	4.72
PXL1	-1.57	5.77
NDC1	-1.57	8.43
PDE1	-1.57	30
TAT2	-1.57	30
MST27	-1.56	2.93
DAL4	-1.56	2.98
SWC7	-1.56	4.70
ERP3	-1.56	5.67
YCG1	-1.56	6.05
ADE13	-1.56	10.36
CBP3	-1.56	11.48
OST4	-1.56	11.84
RRP5	-1.56	30
RRN11	-1.55	6.11
SRL2	-1.55	6.23
SMC3	-1.55	6.56
CPD1	-1.55	7.20
PPS1	-1.55	8.69
NAM2	-1.55	9.81
MID1	-1.55	10.62
SGA1	-1.55	10.97
AIM34	-1.54	3.81
RMP1	-1.54	4.36
SPO75	-1.54	4.38
MAK11	-1.54	5.45
SEC16	-1.54	6.94
COX10	-1.54	7.08
KCH1	-1.54	12.14
HIS4	-1.54	16.66

CRG1	-1.54	30
RPL11A	-1.54	30
SWD2	-1.53	3.98
SEC1	-1.53	4.63
ELM1	-1.53	4.88
CHL1	-1.53	6.36
APL6	-1.53	8.72
PYC2	-1.53	9.24
STV1	-1.53	30
ZRG17	-1.52	3.83
CEX1	-1.52	5.06
HAP5	-1.52	6.25
GAL80	-1.52	12.00
IRC22	-1.52	12.73
SAM2	-1.52	14.84
ANY1	-1.52	17.62
RCY1	-1.51	3.14
ISM1	-1.51	4.50
GEF1	-1.51	5.06
RRP1	-1.51	5.70
SDO1	-1.51	7.77
MLC2	-1.51	7.84
AQR1	-1.51	16.16
RPS20	-1.51	30
PDC5	-1.50	2.26
MTF2	-1.50	5.39
GCS1	-1.50	6.16
FMP41	-1.50	8.09
SNU13	-1.50	8.42
MUK1	-1.50	11.53
PAM18	-1.50	13.72
EMG1	-1.50	14.46
EGD1	-1.50	30
NPR3	-1.49	3.77
ARF3	-1.49	4.59
CDC9	-1.49	5.37
FPR2	-1.49	7.10
MIC19	-1.49	8.03

SPB1	-1.49	9.29
SHE2	-1.49	9.30
GAS5	-1.49	30
BIL1	-1.48	2.51
BMT5	-1.48	3.05
IOC2	-1.48	3.12
NRK1	-1.48	4.50
YGK1	-1.48	5.47
ERR2	-1.48	6.82
RDH54	-1.48	7.77
CCH1	-1.48	8.46
NAN1	-1.48	10.73
PET112	-1.47	2.55
MSE1	-1.47	3.54
VHT1	-1.47	4.94
RGD2	-1.47	5.40
SAE2	-1.47	5.71
RPS12	-1.47	30
AMA1	-1.46	2.83
RRM3	-1.46	4.05
MET16	-1.46	4.20
COG2	-1.46	4.76
STB6	-1.46	6.04
TRM82	-1.46	7.02
GPI16	-1.46	10.19
PRP43	-1.46	10.62
TUB2	-1.46	17.19
RPL20B	-1.46	30
DHR2	-1.45	2.63
TSC11	-1.45	4.36
NGL2	-1.45	5.89
RPS7B	-1.45	7.30
NUP57	-1.45	10.41
CAN1	-1.45	11.18
PMT1	-1.45	30
AIM44	-1.44	2.44
RNH202	-1.44	3.42
THS1	-1.44	30

COQ8	-1.43	2.25
GLE2	-1.43	6.49
CCW12	-1.43	13.74
PUG1	-1.42	2.19
CAX4	-1.42	2.61
CBP2	-1.42	3.38
SRP72	-1.42	4.18
FIN1	-1.42	4.41
SKI8	-1.42	5.33
TRS130	-1.42	5.55
DYN1	-1.42	6.13
PRP19	-1.42	9.26
AQY3	-1.42	16.69
EST3	-1.41	3.61
ZDS2	-1.41	4.85
EMC2	-1.41	5.34
RNR1	-1.41	5.45
SLD7	-1.41	6.52
CYC7	-1.41	30
URA5	-1.41	30
TOS3	-1.40	3.21
TAD3	-1.40	3.50
GAS2	-1.40	3.59
RAD33	-1.40	3.94
MIN4	-1.40	4.91
POL3	-1.40	5.13
RCF1	-1.40	10.41
BUD2	-1.39	2.95
YUH1	-1.39	3.20
POP5	-1.39	3.23
NUP120	-1.39	3.54
UIP3	-1.39	4.44
CYB2	-1.39	6.70
PRP40	-1.39	11.22
GSY2	-1.39	18.49
HIS5	-1.39	30
REV7	-1.38	3.08
NRP1	-1.38	3.72

ESP1	-1.38	3.75
DSE1	-1.38	5.05
MVB12	-1.38	5.98
SFB2	-1.38	6.06
MRP4	-1.38	6.15
PPN2	-1.38	6.55
RNT1	-1.38	7.13
CEM1	-1.38	12.78
SSP1	-1.37	2.14
FUR4	-1.37	3.13
RIC1	-1.37	3.43
EPO1	-1.37	6.24
BNI4	-1.37	6.28
HEM12	-1.37	7.98
FRE6	-1.37	9.55
THR4	-1.37	11.19
RRP3	-1.37	11.49
RTC3	-1.37	30
ORC3	-1.36	4.14
RSF2	-1.36	4.56
MAD1	-1.36	4.67
HSP78	-1.36	30
VMA16	-1.36	30
YET3	-1.36	30
AUS1	-1.35	2.19
PHO81	-1.35	3.31
END3	-1.35	11.21
TPN1	-1.35	13.28
IDP1	-1.35	30
RPS23A	-1.35	30
MIP6	-1.34	2.03
RBH1	-1.34	2.30
MAM1	-1.34	3.16
MLH1	-1.34	3.99
SRP40	-1.34	5.38
ATF1	-1.34	8.33
GCN20	-1.34	13.12
HOL1	-1.34	16.44

TNA1	-1.34	30
RTC1	-1.33	3.10
HRD3	-1.33	4.48
CDC28	-1.33	6.65
CDC12	-1.33	8.24
EMA19	-1.33	8.76
EMC6	-1.33	9.05
YIH1	-1.33	13.30
RPL26A	-1.33	14.75
MSH1	-1.32	2.16
MMP1	-1.32	3.75
PTC2	-1.32	7.07
RFM1	-1.31	2.36
YEA4	-1.31	2.87
COG6	-1.31	3.30
MRS1	-1.31	4.40
SND2	-1.31	5.60
HXT8	-1.31	5.71
RIX7	-1.31	10.26
MRM1	-1.31	15.23
ADK1	-1.31	30
GPH1	-1.31	30
HOM2	-1.31	30
SAC6	-1.31	30
ACM1	-1.30	2.88
GEA1	-1.30	3.17
DEG1	-1.30	4.73
DPB2	-1.30	8.91
TPM2	-1.30	9.16
HRB1	-1.30	10.38
YPT31	-1.30	12.00
CBF5	-1.30	30
MEX67	-1.30	30
ZUO1	-1.30	30
SDD1	-1.29	3.27
GUF1	-1.29	3.29
URA7	-1.29	4.32
RRP12	-1.29	5.55

DUN1	-1.29	5.72
SRY1	-1.29	13.57
ART5	-1.28	2.93
SET4	-1.28	3.03
SNU66	-1.28	3.22
FUN26	-1.28	3.70
SIT1	-1.28	4.31
ZDS1	-1.28	4.97
LAP2	-1.28	6.24
TVP15	-1.28	8.01
NCW2	-1.28	15.32
ERG2	-1.28	30
PAU2	-1.27	2.28
HIR3	-1.27	2.88
DPH6	-1.27	4.26
AFG1	-1.27	5.01
ERR1	-1.27	5.29
YEH1	-1.27	7.01
ADE2	-1.27	7.94
SHM1	-1.27	8.21
NIT3	-1.27	9.52
RPS5	-1.27	30
TEA1	-1.26	2.47
TRS85	-1.26	2.96
PUF6	-1.26	4.16
DOG1	-1.26	7.29
PAM1	-1.26	7.90
YOP1	-1.26	9.60
TOM40	-1.26	12.31
DUG2	-1.25	2.41
WHI4	-1.25	2.93
CAB5	-1.25	7.67
DIP2	-1.25	8.36
UGP1	-1.25	30
STE2	-1.24	2.39
TOF1	-1.24	2.81
MED1	-1.24	3.18
STE50	-1.24	4.09

UBP8	-1.24	4.42
CBC2	-1.24	6.36
SME1	-1.24	9.49
LSC1	-1.24	11.97
PDI1	-1.24	30
HXT17	-1.23	2.34
MCO12	-1.23	3.17
MDE1	-1.23	3.69
TRM10	-1.23	4.09
ISC1	-1.23	4.67
SPC25	-1.23	4.99
RPG1	-1.23	5.65
NUP133	-1.23	6.24
GGA2	-1.23	7.39
VPS13	-1.23	9.29
SEC26	-1.23	11.94
LAT1	-1.23	12.97
POL2	-1.22	2.76
FAF1	-1.22	5.24
CHO2	-1.22	18.71
ARG4	-1.22	30
RPL1B	-1.22	30
BET5	-1.21	3.81
EBS1	-1.21	5.51
LYS2	-1.21	6.51
UTP21	-1.21	6.83
MIC10	-1.21	9.81
ECM33	-1.21	9.93
FSH1	-1.21	17.27
FLO5	-1.20	2.32
ATE1	-1.20	3.13
TFC6	-1.20	3.70
HFA1	-1.20	3.90
RPA12	-1.20	6.10
RPA49	-1.20	8.92
ARP3	-1.20	11.40
RPL35A	-1.20	30
DBF4	-1.19	2.78

MMR1	-1.19	4.12
UTP4	-1.19	5.13
NAF1	-1.19	5.17
CMP2	-1.19	6.23
ECM16	-1.19	8.67
FUM1	-1.19	12.45
GLR1	-1.19	12.76
RPS26B	-1.19	30
TRL1	-1.18	2.54
ERD1	-1.18	3.16
ARG81	-1.18	4.30
SMC4	-1.18	4.93
MNT2	-1.18	5.05
AKR1	-1.18	8.25
ASC1	-1.18	30
SAD1	-1.17	2.38
EXO70	-1.17	2.40
RAX1	-1.17	2.84
NTE1	-1.17	3.63
PET309	-1.17	4.70
LIA1	-1.17	5.74
AIM7	-1.17	6.07
IFH1	-1.17	9.57
ATP1	-1.17	30
QRI7	-1.16	2.03
PCL6	-1.16	2.36
NFT1	-1.16	2.65
IRC5	-1.16	2.71
REB1	-1.16	3.25
BCS1	-1.16	5.12
SPA2	-1.16	5.24
GLN4	-1.16	12.16
ILS1	-1.16	30
HSH155	-1.15	2.68
GRC3	-1.15	3.31
AMF1	-1.15	3.89
MTO1	-1.15	5.10
IST3	-1.15	5.95

UBX2	-1.15	6.30
ATG41	-1.15	7.70
IPP1	-1.15	8.46
CPR6	-1.15	30
ADK2	-1.14	2.01
MDM1	-1.14	2.93
USO1	-1.14	3.57
NDJ1	-1.14	4.94
SAP190	-1.14	5.61
MTM1	-1.14	19.16
RPS24A	-1.14	30
SSE1	-1.14	30
ZEO1	-1.14	30
SLP1	-1.13	2.55
FAB1	-1.13	2.59
BUD6	-1.13	2.69
GWT1	-1.13	3.17
BNA5	-1.13	4.51
VAM7	-1.13	5.07
ENT4	-1.13	5.40
UTP22	-1.13	7.09
GPI17	-1.13	10.40
MCH4	-1.13	11.13
RPS21B	-1.13	30
VPS25	-1.12	3.10
AEP2	-1.12	3.34
SUV3	-1.12	3.72
NOP53	-1.12	5.38
NSG1	-1.12	5.57
MRP1	-1.12	6.95
NOC2	-1.12	7.07
NOT5	-1.12	7.45
NDE1	-1.12	30
RPL12B	-1.12	30
TOG1	-1.11	2.18
NUP82	-1.11	3.54
AIM39	-1.11	4.99
LAG1	-1.11	6.05

MCM4	-1.11	7.37
CCW14	-1.10	7.94
LCB5	-1.10	8.29
RCL1	-1.10	8.67
HMF1	-1.10	14.73
HXT6	-1.10	30
MIL1	-1.09	3.93
PCL9	-1.09	4.27
SPP2	-1.09	6.19
SDH4	-1.09	11.26
RPL42A	-1.09	16.51
RPL42B	-1.09	30
SHM2	-1.09	30
SLT2	-1.09	30
PMT6	-1.08	2.47
BER1	-1.08	2.75
SGO1	-1.08	3.15
LCP5	-1.08	4.01
YTM1	-1.08	5.63
SAY1	-1.07	2.35
CMG1	-1.07	2.71
IRR1	-1.07	3.54
ROD1	-1.07	3.81
RPS0A	-1.07	13.99
RGT1	-1.07	18.26
RPL16B	-1.07	30
RPS1B	-1.07	30
SOV1	-1.06	2.62
BOL1	-1.06	3.28
SPR28	-1.06	4.01
RPL6A	-1.06	7.00
TAF9	-1.06	11.67
TRP5	-1.06	16.83
AHK1	-1.05	3.14
HDA2	-1.05	4.93
ZRT2	-1.05	5.36
MCM3	-1.05	5.60
PET494	-1.05	5.80

ADE12	-1.05	12.29
HAP4	-1.05	13.01
PUN1	-1.05	13.21
MSS2	-1.04	2.08
CWP2	-1.04	3.12
XDJ1	-1.04	11.84
SLH1	-1.03	2.58
TPT1	-1.03	2.58
NGG1	-1.03	3.47
CDC7	-1.03	4.72
ZPS1	-1.03	6.03
RBK1	-1.03	7.60
PRS5	-1.03	9.62
SSZ1	-1.03	16.12
MNL2	-1.02	2.83
PTC5	-1.02	3.77
VPS45	-1.02	3.92
CNN1	-1.01	2.17
MRD1	-1.01	5.27
RPL31A	-1.01	12.90
MUP3	-1.00	2.63
SEC15	-1.00	2.88
PTC4	-1.00	3.00
STE18	-1.00	3.14
SAS4	-1.00	3.19
TMA46	-1.00	3.55
LCB3	-1.00	5.06
DPC29	-1.00	6.63
RET3	-1.00	11.54
RPL8A	-1.00	30
RPL8B	-1.00	30

a) -Log10 (p-value) greater than 30 is indicated as 30.

Table S9. Genes whose expression was upregulated and downregulated in the all 4 acquired mutant strains

Upregulated gene or locus tag	Downregulated gene or locus tag
15S_RRNA	AAC3
AAC1	AAD3
ACO1	AAD4
ACS1	AAP1
ADH2	ABP1
ADR1	ABZ2
ADY2	ACA1
ADY4	ACO2
AFG3	ACP1
AFT2	ACS2
AGP1	ACT1
AHC1	ADA2
AI1	ADD37
AI2	ADE1
AI3	ADE17
AI4	ADE3
AI5_ALPHA	ADE6
AI5_BETA	ADE8
AIM19	ADH1
AIM46	ADH6
AIM9	ADK1
ALD2	ADO1
ALD3	AFR1
ALD4	AFT1
ALP1	AGC1
ALT2	AGX1
AMS1	AHP1
APC11	AIM10
APE3	AIM14
APJ1	AIM17
AQY1	AIM24
ARA1	AIM44
ARA2	AIM45
ARE2	AIM7

ARF2	ALD5
ARG80	ALG1
ARG82	ALG3
ASG1	ALG5
ASK1	ALG6
ASK10	ALK1
ASN2	ALK2
ASR1	ALR2
AST2	ALT1
ATG1	AMN1
ATG11	ANB1
ATG15	ANS1
ATG19	APC5
ATG20	APE1
ATG21	APL3
ATG26	APL6
ATG32	APM4
ATG34	AQR1
ATG36	AQY3
ATG39	ARC19
ATG4	ARC40
ATG5	ARF3
ATG8	ARG2
ATG9	ARG7
ATH1	ARH1
ATO2	ARI1
ATO3	ARL1
ATP25	ARO1
ATP6	ARO4
ATP8	ARO7
AVT3	ARO8
AZF1	ARP1
BAG7	ARP2
BAP2	ARP3
BCP1	ARP5
BDF1	ASH1
BDF2	ASN1
BDH2	AST1

BEM1	ATC1
BI2	ATG41
BI3	ATM1
BI4	ATP1
BIG1	ATP2
BLM10	ATP5
BOP2	ATX2
BPT1	AVT1
BRE1	AVT5
BRE4	AXL1
BSC2	AXL2
BSC4	AZR1
BUD23	BAP3
BXI1	BAR1
BYE1	BBP1
CAB2	BCH1
CAB4	BDH1
CAF120	BDS1
CAF40	BET5
CAR2	BFR1
CAT2	BIK1
CAT8	BIO5
CBF1	BIT61
CCC1	BNA1
CCL1	BNA3
CCS1	BNA6
CCT2	BNI4
CCT3	BOL3
CCT5	BRP1
CCT6	BSC5
CCT8	BTN2
CDC123	BUD14
CDC16	BUD3
CDC23	BUD4
CDC27	BUD7
CDC31	BUD9
CDC34	CAF16
CDC37	CAR1

CDC40	CBP2
CDC53	CBT1
CDD1	CCA1
CDH1	CCH1
CFD1	CCW12
CHK1	CCW14
CHZ1	CDA1
CIP1	CDC10
CIS1	CDC11
CIT2	CDC12
CIT3	CDC14
CKB2	CDC28
CLD1	CDC3
CMC4	CDC45
CMI8	CDC5
CMR3	CEX1
COA3	CGI121
COA4	CHA1
COB	CHC1
COQ21	CHL1
COQ4	CHS3
COS9	CHS7
COX1	CIR1
COX11	CIS3
COX16	CKI1
COX2	CLA4
COX23	CLB1
COX26	CLB2
COX3	CLB4
COX7	CLN1
CPR1	CLN2
CPS1	CLP1
CPT1	CMP2
CRC1	COG2
CRF1	COG3
CRP1	COG4
CSC1	COG8
CSG2	COM2

CSM4	COP1
CSR2	COQ2
CSS3	COQ3
CST6	COS2
CTA1	COS3
CTF19	COS5
CTK3	COS8
CTR2	COX10
CUP2	COX5B
CUZ1	CPD1
CWC23	CPR5
CWC24	CPR6
DAN1	CRG1
DAN4	CRH1
DAS1	CRN1
DAT1	CRS1
DBP1	CSI2
DBP2	CTF13
DCS2	CTF18
DDI1	CTH1
DDP1	CTR1
DDR2	CUP1-1
DEF1	CUP1-2
DIA1	CUP9
DIN7	CUR1
DIP5	CWH43
DLD3	CYB2
DOC1	CYB5
DPA10	CYC1
DPB4	CYC2
DPC25	CYC7
DPC7	CYC8
DPL1	CYK3
DSC2	CYS3
DSK2	DAL1
EAF3	DAL4
EAR1	DAL5
ECI1	DAL7

ECM10	DAS2
ECM2	DBF2
ECM21	DBP6
ECM27	DCD1
ECM4	DDI2
EDC2	DED81
ELA1	DEG1
ELP4	DIA2
EMP46	DIT2
ENA1	DOA1
ENA2	DOG1
ENA5	DOG2
ERC1	DOP1
ERV1	DOT1
ESA1	DOT5
ESL2	DPC29
FAA1	DPH2
FAA2	DPH6
FAA4	DPP1
FAP1	DRS1
FAT3	DRS2
FBP1	DSE4
FET5	DSF1
FEX2	DSF2
FHN1	DUG1
FIS1	DUN1
FKS3	DUT1
FLC2	EBP2
FMP10	ECM16
FMP16	ECM18
FMP23	ECM31
FMP40	ECM33
FMP45	EDS1
FMP46	EFR3
FMP48	EGD1
FMP49	EGD2
FMP52	EGH1
FMS1	EGO4

FOX2	EGT2
FPK1	EHT1
FRQ1	ELM1
FTH1	ELO1
FUB1	ELO3
FYV10	EMA19
FYV6	EMC1
FYV8	EMC5
FZF1	EMG1
FZO1	EMI2
GAC1	EMP24
GAL83	EMP70
GAP1	END3
GAT2	ENO2
GDH2	ENT1
GDH3	EPO1
GET3	EPT1
GFD1	ERD2
GID8	ERG1
GIM4	ERG11
GIP1	ERG12
GIP2	ERG13
GIS1	ERG20
GIS3	ERG26
GLC3	ERG27
GLC8	ERG28
GLN1	ERG4
GLO1	ERG5
GLO4	ERG6
GLT1	ERG8
GNP1	ERP2
GOS1	ERR1
GPA2	ERR2
GPG1	EXG1
GPI10	EXG2
GPI19	EXP1
GPI2	FAL1
GPN2	FAR1

GPT2	FCY1
GPX1	FDC1
GRE1	FDO1
GRE2	FET3
GRR1	FET4
GRX3	FIT2
GRX4	FKS1
GRX6	FLO5
GSC2	FPR2
GSH1	FPR4
GTO1	FRD1
GTO3	FRE1
GTT1	FRE3
GUT2	FRE7
GYP7	FRM2
HAA1	FRS1
HAL9	FSH1
HBT1	FSH3
HEF3	FTR1
HEL1	FUM1
HEM2	GAL10
HFD1	GAL80
HFL1	GAR1
HHF1	GAS1
HHO1	GAS3
HIT1	GAS5
HMS1	GAT4
HOT1	GCN1
HPA2	GCV1
HRT1	GCV2
HRT3	GCV3
HSF1	GDA1
HSM3	GDB1
HST1	GDE1
HSV2	GEA2
HTA2	GEF1
HTL1	GEP3
HTZ1	GFA1

HUA1	GFD2
HUB1	GGC1
HUL5	GIC1
HUR1	GIM3
HVG1	GIM5
HXT11	GIN4
HXT4	GLR1
HXT5	GNA1
HXT9	GND1
HYM1	GOR1
HYR1	GPA1
IBA57	GPH1
IBD2	GPI16
ICL1	GPM3
ICL2	GRE3
ICT1	GRS1
ICY1	GRX8
IDP3	GSH2
ILT1	GSP1
IMP2'	GSY2
INA17	GTT2
INH1	GUA1
INO1	GUK1
INO2	GUP2
INO4	GUS1
INP54	HAM1
IOC4	HAT2
IRA1	HCS1
IRA2	HDA1
IRC13	HED1
IRC15	HEM25
IRC20	HER2
ISA1	HGH1
ISF1	HIM1
ISU1	HIP1
ISY1	HIS2
ITT1	HIS5
IWR1	HIS6

IZH4	HOF1
JEN1	HOL1
JHD1	HOM2
JID1	HOM3
KEL1	HRI1
KGD2	HSP10
KHA1	HSP150
KIC1	HSP30
KNS1	HSP78
KRE1	HTC1
KTR1	HTS1
KTR4	HXK1
LAP3	HXK2
LCL1	HXT1
LDB7	HXT13
LDO16	HXT14
LEE1	HXT15
LEU3	HXT16
LGE1	HXT3
LPL1	HXT6
LPX1	HXT7
LRE1	HXT8
LSO1	IDP1
LSP1	IFA38
LUG1	IMA2
LYS20	IMA5
MAF1	IMD2
MAG1	IMD4
MAG2	INP53
MAM3	IPK1
MAP1	IPL1
MATALPHA2	IQG1
MCH1	IRC10
MCM1	IRC22
MCO10	IRC7
MCO32	IRC8
MCO76	ISM1
MCO8	JLP2

MDH1	KAE1
MDH2	KAP122
MDH3	KAR1
MDM34	KAR2
MDV1	KAR3
MED2	KCH1
MED7	KDX1
MEF2	KEL3
MEP1	KIN3
MEP2	KIN4
MET28	KIP2
MET30	KRE6
MET4	KRE9
MET8	KRS1
MGA2	KTI12
MGM1	KTR2
MGR3	KTR3
MHO1	KTR5
MIA40	LAP2
MIC26	LCB5
MIH1	LDB17
MIM1	LDS1
MIN7	LNP1
MIN8	LOA1
MIT1	LOT6
MIX17	LRO1
MKK1	LSB1
MKK2	LSC1
MLO1	LSC2
MLS1	LTP1
MNC1	LTV1
MND1	LYS1
MNE1	LYS12
MNN14	LYS4
MNN5	LYS9
MOH1	MAD1
MOT2	MAK10
MPC1	MAK11

MPC3	MAL12
MPD2	MAL32
MPE1	MAN2
MPM1	MAS2
MPO1	MCD1
MPS3	MCD4
MRK1	MCH5
MRP2	MCM16
MRPL10	MCM2
MRPL16	MCM5
MRPL28	MCO14
MRPL51	MDL1
MRPS18	MDL2
MRS6	MED1
MRX8	MEI4
MRX9	MES1
MSC1	MET10
MSC3	MEX67
MSG5	MGL2
MSN2	MIC10
MSN5	MIC19
MSP1	MIC60
MUB1	MID2
MUM2	MIN4
MVP1	MIN9
MXR1	MIS1
NAB3	MKC7
NAM9	MLC1
NAR1	MLO50
NAS6	MME1
NBL1	MMP1
NBP35	MMR1
NCA3	MNL2
NCB2	MNT2
NCR1	MOB2
NDE2	MON2
NDL1	MPC2
NFI1	MPD1

NFS1	MPH1
NGL3	MPH2
NGR1	MPH3
NHP6B	MRC1
NHX1	MRH1
NIS1	MRM1
NNF2	MRP51
NNK1	MRPL11
NPC2	MRPL25
NPR1	MRPL32
NQM1	MRPL7
NRD1	MRS1
NST1	MRS3
NTH2	MRT4
NTO1	MRX1
NUD1	MRX15
NUM1	MRX4
NUP2	MSF1
NUR1	MSH2
NYV1	MST1
OAF1	MST27
OAZ1	MSW1
OLE1	MSY1
OLI1	MTM1
OM14	MTO1
OM45	MTQ1
OPI1	MUK1
OPY1	MVB12
OPY2	MVD1
ORM1	MYO1
ORM2	MYO2
OTU1	MYO3
PAH1	MYO4
PAI3	MYO5
PAL2	NAT1
PAN2	NAT5
PAN6	NCL1
PAR32	NCP1

PAU19	NCW1
PBA1	NCW2
PBI2	NDC1
PCI8	NDJ1
PCK1	NDT80
PCL10	NGL2
PCL8	NIT3
PCP1	NMA1
PCT1	NMD4
PDC6	NMT1
PDH1	NOP53
PDR1	NOP56
PDR10	NOP58
PDR12	NOT5
PDR8	NPY1
PEP4	NSA1
PET100	NTE1
PET130	NTG1
PET20	NUC1
PEX11	NUG1
PEX15	NUP145
PEX18	NUP170
PEX19	NUP188
PEX22	NUP84
PEX28	NUP85
PEX3	NUT1
PEX30	ODC2
PEX31	OMS1
PEX5	OPI10
PEX9	ORC1
PFK26	ORT1
PFU1	OSH6
PGA2	OST4
PGA3	OSW1
PGD1	OTU2
PHB2	OYE2
PHM7	PAL1
PHO2	PAM1

PHO4	PAM18
PIB1	PAN5
PIG2	PAU23
PIL1	PAU6
PKH2	PBI1
PLB2	PCL5
PLB3	PCM1
PMC1	PDC1
PMP3	PDE1
PMR1	PDI1
POT1	PEP3
POX1	PER1
PPQ1	PER33
PRB1	PES4
PRC1	PET8
PRE1	PEX6
PRE10	PFK27
PRE2	PFY1
PRE3	PGM1
PRE4	PHD1
PRE5	PHO12
PRE7	PHO13
PRE9	PHO3
PRM3	PHO91
PRP18	PHR1
PRP31	PHS1
PRR2	PIC2
PSH1	PIF1
PSP1	PIN2
PTC1	PIR1
PTI1	PIR3
PTK1	PKR1
PTR2	PLP2
PUP1	PMA1
PUP2	PML39
PUT1	PMT1
PUT2	PMT2
PUT3	PMT5

PUT4	PMU1
PXA1	PNP1
PXA2	POF1
PYC1	POL1
QCR6	POL5
QDR1	POM33
QNS1	POP1
RAD1	PPN2
RAD14	PPS1
RAD16	PPZ1
RAD23	PRM10
RAD30	PRM5
RAD4	PRM8
RAD50	PRO1
RAD51	PRO3
RAD54	PRP19
RAD55	PRP42
RAM1	PRP5
RAP1	PRR1
RAS1	PRS4
RCF3	PSA1
RCI37	PSD1
RCI50	PSD2
RCN2	PST1
RCR1	PTC2
REF2	PTM1
REH1	PUF4
RFA1	PUL3
RFA2	PUS1
RFA3	PUS4
RGI2	PUS6
RGS2	PUS7
RHO1	PWP1
RIB1	PXL1
RIM101	PYC2
RIM4	QCR2
RIT1	QDR2
RKM5	RAD59

RMD1	RAI1
RMD5	RAM2
RNA14	RAX1
RNH70	RAX2
RNQ1	RBD2
RNR2	RBH2
RNR3	RBS1
ROG1	RCF1
ROX3	RCK1
ROY1	RCM1
RPB4	RCY1
RPC31	RDH54
RPI1	RDS1
RPM1	REE1
RPN1	RET3
RPN13	REX2
RPN3	RFC1
RPN6	RFC5
RPN7	RGA1
RPN8	RGI1
RPO31	RGT1
RPS27A	RHO2
RPS27B	RIA1
RPS30B	RIM21
RPT1	RKM2
RPT2	RKM4
RPT3	RME1
RPT4	RNH203
RPT6	ROD1
RQC1	ROM1
RRD1	RPA49
RRD2	RPC25
RRG1	RPC82
RRN3	RPG1
RRT1	RPL11A
RRT6	RPL11B
RRT8	RPL12A
RSA1	RPL12B

RSB1	RPL15A
RSC2	RPL16B
RSC4	RPL18A
RSF1	RPL1B
RSM22	RPL20B
RSN1	RPL22A
RTC6	RPL23B
RTG1	RPL26A
RTK1	RPL2A
RTN2	RPL2B
RTS2	RPL31A
RTS3	RPL34A
RUB1	RPL35A
RVS161	RPL42A
SAF1	RPL42B
SAN1	RPL6A
SAP155	RPL8A
SAW1	RPL8B
SCD5	RPL9A
SCH9	RPN14
SCS2	RPO26
SCS7	RPP0
SDD3	RPP1
SDH2	RPP2A
SDH6	RPS0A
SDH8	RPS11B
SDP1	RPS12
SEC17	RPS17B
SEF1	RPS18B
SEG1	RPS1B
SEM1	RPS20
SEN2	RPS21B
SEN54	RPS22A
SFA1	RPS23A
SFC1	RPS26B
SFL1	RPS28B
SGT1	RPS5
SHC1	RPS8B

SHE10	RRB1
SHH3	RRF1
SHH4	RRG7
SHP1	RRI2
SHR5	RRM3
SHU2	RRP1
SIP18	RRP3
SIP2	RRP45
SKN1	RRP5
SKN7	RRT12
SKP1	RRT5
SKS1	RSC3
SLG1	RSF2
SLM3	RSR1
SLZ1	RTC1
SNA3	RTC3
SNA4	RTC4
SNC1	RUD3
SNC2	SAC1
SND1	SAC6
SND3	SAE2
SNF1	SAE3
SNF11	SAH1
SNF3	SAL1
SNG1	SAM1
SNQ2	SAM2
SNT309	SAM4
SNX4	SAP185
SOK2	SAP4
SOL2	SCO2
SPC42	SCP1
SPF1	SCP160
SPG1	SCW10
SPG3	SCW4
SPG4	SDD1
SPG5	SDO1
SPO20	SEC1
SPO24	SEC10

SPP1	SEC11
SPP41	SEC16
SPS100	SEC21
SPS19	SEC23
SPT16	SEC27
SPT5	SEC28
SPT6	SEC3
SRC1	SEC31
SRL4	SEC59
SRP54	SEC61
SSD1	SEC62
SSH4	SEC63
SSL2	SEC65
SSM4	SEC7
SSU1	SED4
SSU72	SER2
STB3	SER3
STE11	SES1
STE24	SET4
STE7	SET5
STF1	SFB3
STP1	SGA1
STR3	SHE1
SUA7	SHE2
SUE1	SHE3
SUM1	SHM1
SUR1	SHM2
SUR2	SHS1
SUT2	SIA1
SWC5	SIL1
SWF1	SIM1
SWI1	SIN4
SYG1	SIP3
TAD2	SKG1
TAF12	SKG6
TAF4	SKI2
TBS1	SKI8
TCP1	SKM1

TDA11	SKT5
TFB4	SLD3
TGL4	SLD7
TIM13	SLT2
TMH11	SMC2
UBC6	SME1
UBR1	SMF1
UBX4	SMF2
UBX5	SMI1
UCC1	SMP1
UFD1	SNF8
UGA1	SNO1
UGA2	SNO2
UME1	SNO3
UME6	SNU13
UMP1	SNZ2
VHS2	SNZ3
VHS3	SOL3
VID27	SOP4
VID28	SOR1
VID30	SOR2
VPS24	SPB1
WTM2	SPC24
YAT2	SPC29
YSA1	SPE4
ZTA1	SPH1
	SPO74
	SPO75
	SPO77
	SPR28
	SPT14
	SPT4
	SRB2
	SRD1
	SRL1
	SRL3
	SRM1
	SRO9

	SRP101
	SRX1
	SRY1
	SSA1
	SSA2
	SSA4
	SSB1
	SSE1
	SSK22
	SSO2
	STD1
	STE4
	STE5
	STE50
	STE6
	STT3
	STU1
	STU2
	STV1
	SUC2
	SUN4
	SUR7
	SUT1
	SVS1
	SWE1
	SWI4
	SWI5
	SWI6
	SYM1
	TAE1
	TAF11
	TAF2
	TAL1
	TAN1
	TAT2
	TCB2
	TCD2
	TDH2

	TEF4
	THI12
	THR4
	THS1
	TMA20
	TOS4
	TPM2
	TTI1
	TUB2
	TUB3
	URA6
	URB2
	UTP10
	VTH2
	XKS1
	YAP7
	YEH1
	YMC1
	YPS6
	YRF1-2
	YRF1-3
	YRF1-4
	YRF1-5
	YRF1-6
	YRF1-7
	YRF1-8
	YSC84
	YSP3
	YVH1

Highlighted genes may function as transcription factors.
